# Tandem diazotization/cyclization approach for the synthesis of a fused 1,2,3-triazinone-furazan/furoxan heterocyclic system

**DOI:** 10.3762/bjoc.20.200

**Published:** 2024-09-16

**Authors:** Yuri A Sidunets, Valeriya G Melekhina, Leonid L Fershtat

**Affiliations:** 1 N.D. Zelinsky Institute of Organic Chemistry, Russian Academy of Sciences, 119991, Leninsky Prospect, 47, Moscow, Russian Federation; 2 National Research University Higher School of Economics 101000, Myasnitskaya str., 20, Moscow, Russian Federation

**Keywords:** molecular hybridization, nitric oxide, nitrogen heterocycles, 1,2,5-oxadiazoles, 1,2,3-triazin-4-one

## Abstract

A straightforward protocol for the synthesis of a previously unknown [1,2,5]oxadiazolo[3,4-*d*][1,2,3]triazin-7(6*H*)-one heterocyclic system was developed. The described approach is based on tandem diazotization/azo coupling reactions of (1,2,5-oxadiazolyl)carboxamide derivatives bearing both aromatic and aliphatic substituents. The NO-donor ability of the synthesized furoxano[3,4-*d*][1,2,3]triazin-7(6*H*)-ones was additionally evaluated. The elaborated method provides access to novel nitrogen heterocyclic compounds with potential applications as drug candidates or thermostable components of functional organic materials.

## Introduction

Nitrogen heterocycles are a significant and broad class of organic substances included in the structure of various natural products and pharmacologically active molecules. For example, nucleic acids, proteins and enzymes, hormones and vitamins, essential for the functioning of a living organism, also contain nitrogen frameworks [1–2]. Besides that, nitrogen-containing compounds are widely used in medicine as antibiotics, anticancer, non-steroidal anti-inflammatory, antihypertensive, antipsychotic, anxiolytic and in other pharmaceuticals [3–5]. Therefore, considering the diversity of biological properties, development of reliable approaches for the synthesis of new nitrogen heterocyclic systems is a highly urgent goal.

1,2,5-Oxadiazoles (furazans) and their *N*-oxides (furoxans) are important representatives of nitrogen heterocycles due to their wide applications in various fields of medicine, chemistry, and materials science [6–7]. For example, these heterocycles serve as valuable building blocks for the synthesis of high-energy materials [8–13]. Moreover, furazan derivatives possess antiproliferative, antibacterial, antiparasitic and antiviral activity [14–16]. On the other hand, furoxans referred to as unique heterocyclic compounds that exhibit NO-releasing properties under physiological conditions and do not demonstrate nitrate tolerance. Nitric oxide (NO) is a signaling molecule that plays a key role in numerous physiologic and pathologic processes. Thus, NO regulates blood flow and tissue oxygenation, so disruption of the production and transport of NO in the vascular system leads to various diseases [17–20]. Therefore, due to their NO-releasing abilities, furoxan derivatives also demonstrate anticancer, antiplatelet, antiviral and antiparasitic properties [21–32].

**Figure 1:**
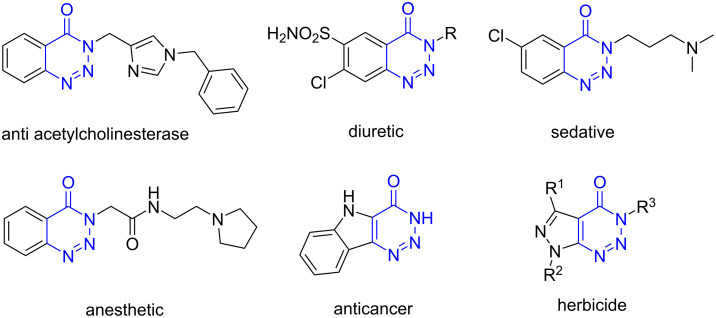
Examples of bioactive compounds containing the 1,2,3-triazin-4-one core.

Another valuable nitrogen heterocyclic scaffold in medicinal chemistry is 1,2,3-triazin-4-one. Such compounds exhibit a wide variety of biological activities including antitumor, anticonvulsant, diuretic, anesthetic and sedative effects [33–36]. Also, several commercially available pharmaceuticals used as herbicidal, antibacterial, fungicidal and insecticidal agents contain a 1,2,3-triazine ring [37–39]. The structures of some bioactive 1,2,3-triazin-4-one derivatives are shown in Figure 1. Hence, one can assume that molecular hybridization of the 1,2,3-triazin-4-one moiety with the 1,2,5-oxadiazole core can lead to a significant modification of the pharmacological properties and may find application in the design of new promising medications.

**Scheme 1:**
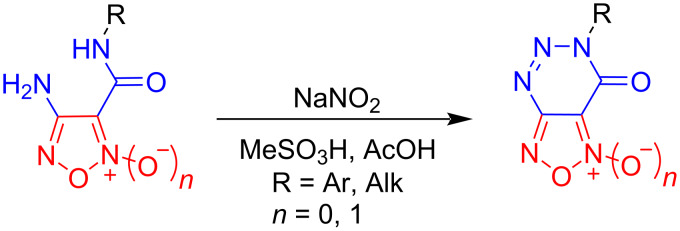
Tandem diazotization/azo coupling reactions of (1,2,5-oxadiazolyl)carboxamides containing an amino functionality.

Herein, we present a convenient synthetic approach for the preparation of previously unknown [1,2,5]oxadiazolo[3,4-*d*][1,2,3]triazin-7(6*H*)-one heterocyclic systems containing both a furoxan/furazan fragment condensed to a 1,2,3-triazin-4-one core. The proposed method is based on tandem diazotization/azo coupling reactions of the corresponding amides (Scheme 1). In addition, application perspectives of thus prepared heterocyclic entities as thermally stable components of functional organic materials or NO-donor drug candidates are also unveiled.

## Results and Discussion

We started our investigations toward the development of the desired synthetic approach to [1,2,5]oxadiazolo[3,4-*d*][1,2,3]triazin-7(6*H*)-one 2-oxides **1** using functionalized furoxans **2** and **3** as suitable substrates. The starting amide precursors **2** were synthesized via the reaction of the readily available 4-amino-3-(azidocarbonyl)-1,2,5-oxadiazole 2-oxide (**3**) with various amines, following a previously described procedure (see Supporting Information File 1 for details) [40–41]. Subsequently, we investigated the possibility of tandem diazotization/azo coupling reactions of the obtained compounds **2**. It should be emphasized that amino-1,2,5-oxadiazoles correspond to very weak nucleophiles due to the highly electron-withdrawing effect of the heterocycle. Our previous efforts achieved a certain result indicating that (1,2,5-oxadiazolyl)diazonium salts, whether isolated or generated in situ, may undergo various controlled transformations [42]. However, previously, we failed to introduce amino-1,2,5-oxadiazoles bearing an amide functionality into the diazotization protocol, arguably due to an increased electron-withdrawing effect and elevated instability of thus generated diazonium salts. In this regard, amide **2a** and mesitylene were selected as model objects to optimize the reaction conditions, since azo coupling of (1,2,5-oxadiazolyl)diazonium salts with electron-donating arenes is known to proceed quantitatively [42]. We varied the diazotization reagents, solvents and temperature, and the obtained results are summarized in Table 1.

**Table 1:** Optimization of the diazotization of amide **2a**^a^.

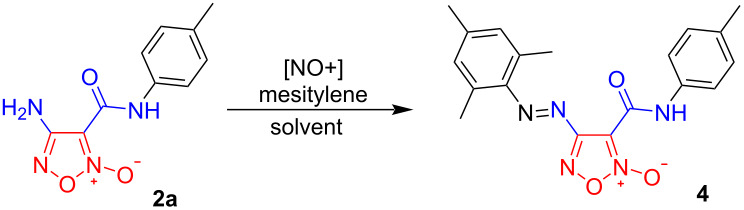				

№	[NO^+^]	Solvent	*T*^o^C	Yield, % ^b^

1	NaNO_2_	TFA	0–5	18
2	NOBF_4_	TFA	0–5	20
3	NaNO_2_	TFA	−10–0	30
4	NOBF_4_	TFA	−10–0	33
5	NaNO_2_	TFA + AcOH [1:1]	−10–0	35
6	NOBF_4_	TFA + AcOH [1:1]	−10–0	49
7	NaNO_2_	MeSO_3_H + AcOH [1:1]	−10–0	86
8	NOBF_4_	MeSO_3_H + AcOH [1:1]	−10–0	12

^a^Reaction conditions: **2a** (0.5 mmol, 0.12 g), nitrosating reagent (0.53 mmol), solvent (3 mL), stirring at the indicated temperature for 20 min, then mesitylene (0.5 mmol, 0.07 mL), stirring at 20 ^o^C for 10 min. ^b^Isolated yield.

Initially, NaNO_2_ and NOBF_4_ were chosen as nitrosating reagents in TFA solution. In all cases (Table 1, entries 1–4), the formation of the target product **4** was observed, but the yield did not exceed 33%. Apparently, such low yield of 4-(mesityldiazenyl)-3-(*p*-tolylcarbamoyl)-1,2,5-oxadiazole 2-oxide (**4**) is likely due to the moderate solubility of the starting amide **2a** in TFA. To improve the solubility of compound **2a**, we tested mixtures of acids as solvent (Table 1, entries 5–8). Thus, the best yield of product **4** was obtained using NaNO_2_ in an AcOH + MeSO_3_H [1:1] solution (Table 1, entry 7). Organic solvents (CH_2_Cl_2_, MeCN) were not applied due to a known rapid decomposition of the generated (1,2,5-oxadiazolyl)diazonium salts [42].

**Scheme 2:**
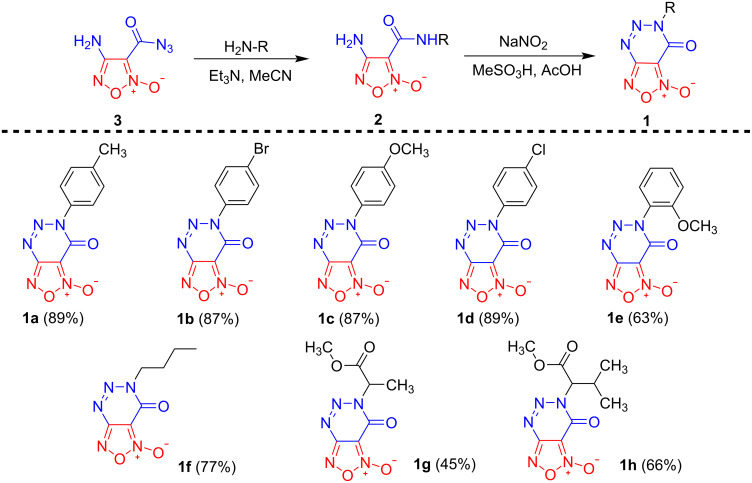
Synthesis of target furoxanotriazinones **1a**–**h**.

The optimized conditions were applied for the preparation of [1,2,5]oxadiazolo[3,4-*d*][1,2,3]triazin-7(6*H*)-one 2-oxides **1** (Scheme 2). Note, that triazinones **1a**–**d** bearing aryl substituents at position 6 were obtained in high yields, however, in the case of the 2-methoxyphenyl derivative the yield of target triazinone **1e** was somewhat lower arguably due to steric hindrance. To our delight, furoxancarboxamides **2f**–**h** bearing aliphatic substituents or amino acid residues also smoothly underwent the studied tandem protocol and the corresponding biheterocyclic compounds **1f**–**h** were obtained in yields of 45–77%.

**Scheme 3:**
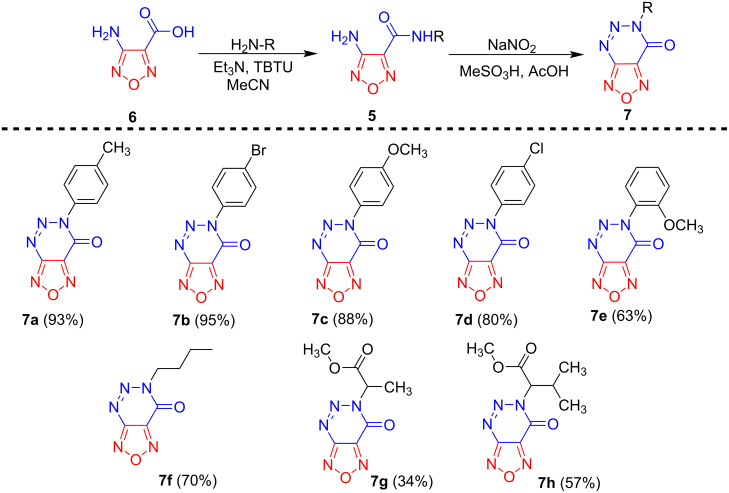
The synthesis of furazanotriazinones **7a**–**h**.

After having developed a general method for the synthesis of target furoxanotriazinones **1a**–**h**, we extended this approach to amides **5** containing a furazan ring that were obtained via the reaction of readily available 4-amino-3-furazancarboxylic acid **6** with various amines using 2-(1*H*-benzotriazole-1-yl)-1,1,3,3-tetramethylaminium tetrafluoroborate (TBTU) as a coupling reagent (Scheme 3; see Supporting Information File 1 for details) [43]. As expected, amides **5** also undergo tandem diazotization/azo coupling reaction to form the target [1,2,5]oxadiazolo[3,4-*d*][1,2,3]triazin-7(6*H*)-ones **7**. It should also be noted, that compounds **7** were obtained in similar yields as the corresponding furoxan analogues, indicating that the developed tandem protocol does not depend on the presence of the *N*-oxide moiety in the parent heterocycle.

**Figure 2:**
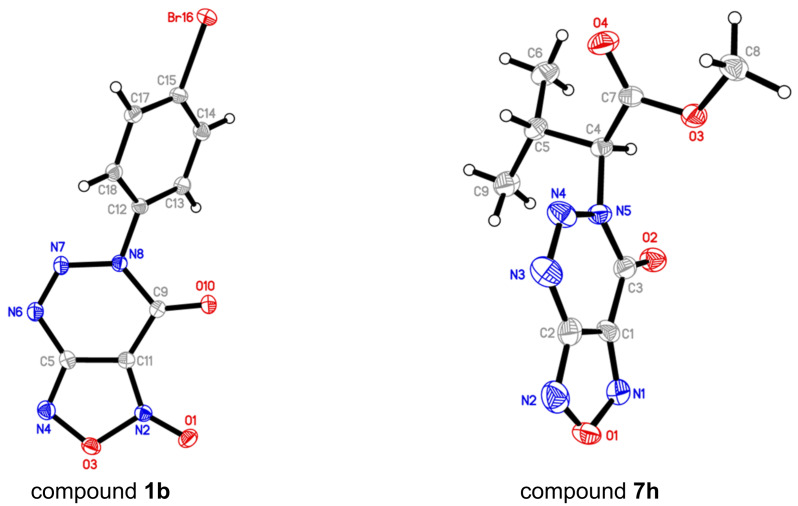
The X-ray structure of compound **1b** (CCDC 2363621) and **7h** (CCDC 2363622).

All synthesized triazinones **1** and **7** were fully characterized by IR, ^1^H and ^13^C NMR spectroscopy, and high-resolution mass spectrometry. The structure of compounds **1b** and **7h** was additionally confirmed by X-ray diffraction (see Supporting Information File 1 for details) (Figure 2).

**Scheme 4:**
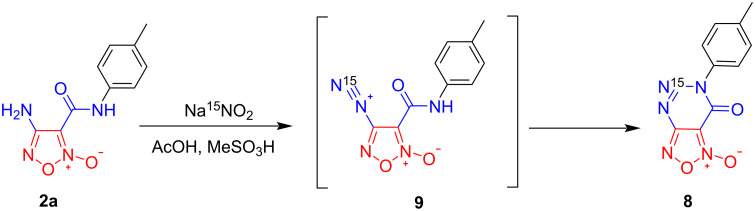
Control experiment with Na^15^NO_2_.

To confirm the reaction mechanism, we performed diazotization followed by azo coupling of amide **2a** using labeled Na^15^NO_2_ as the nitrosating reagent (Scheme 4). As a result, ^15^N-labeled triazinone **8** was obtained. Thus, we have demonstrated that the terminal nitrogen atom in the diazonium fragment of intermediate **9** becomes the N5 atom of compound **8** (corresponding ^15^N NMR spectra are provided in Supporting Information File 1).

**Figure 3:**
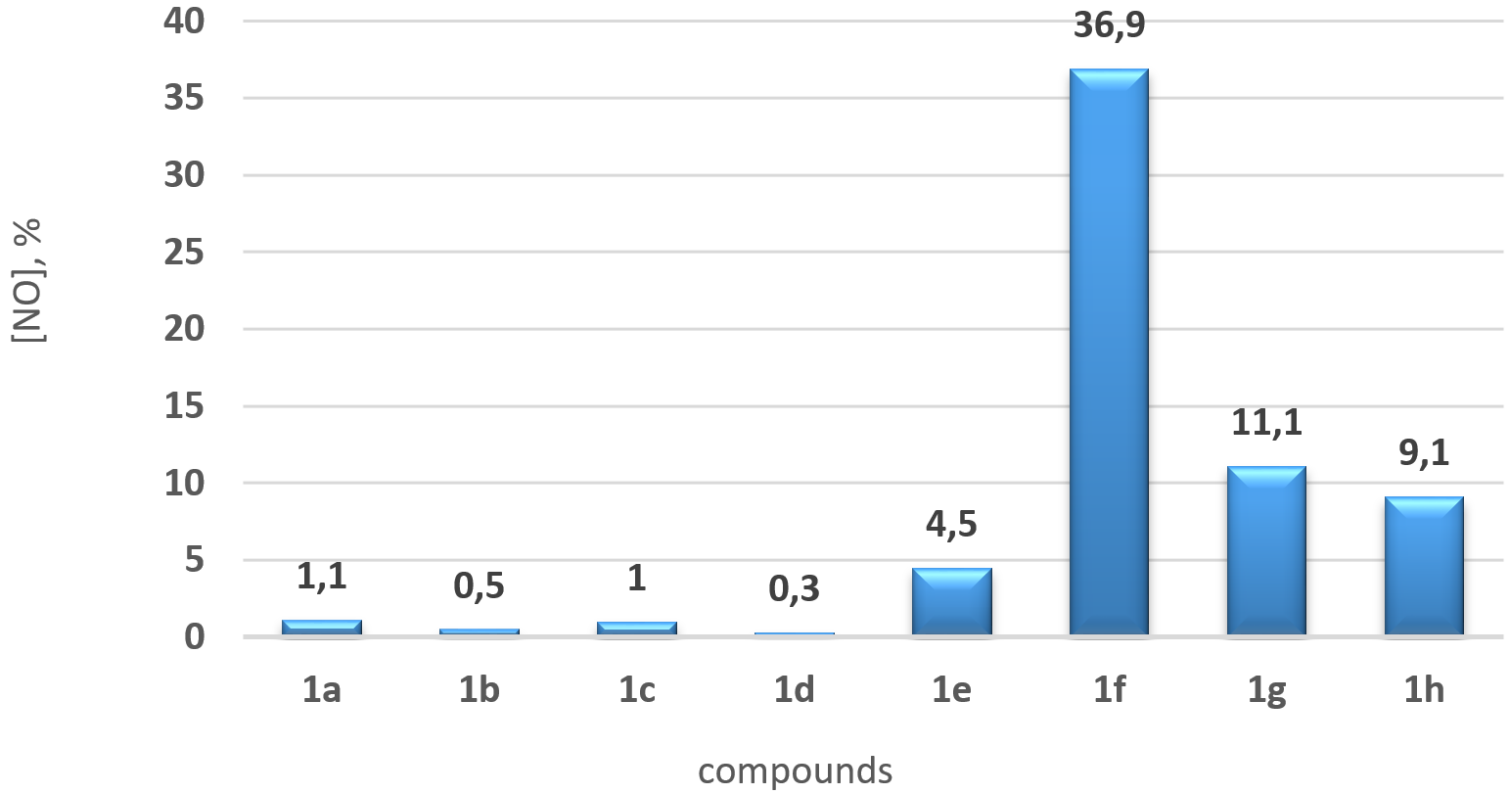
NO release data.

To explore the potential application of the obtained compounds **1** and **7**, we conducted a series of studies. Thus, due to the presence of a furoxan fragment, triazinones **1** can act as NO-donors. To assess their NO-release capability, compounds **1** were kept for 1 hour under physiological conditions (pH 7.4, 37 °C), then Griess reagent was added and studied by spectrophotometry (this reagent detects nitrite formed by the enzymatic oxidation of NO) [44–45]. As shown in Figure 3, compounds **1a**–**e** containing an aryl substituent at position 6 exhibited low NO-donor ability (0.3–4.5%). In contrast, compounds **1f**–**h** with an aliphatic fragment showed moderate activity, with the maximum value recorded for compound **1f** – 36.9%. Therefore, the synthesized triazinones **1** exhibit a wide range of NO-releasing properties and could be considered as potential drug candidates.

Additionally, thermal stability of the obtained triazinones **1** and **7** was evaluated by differential scanning calorimetry. The experiments demonstrated that derivatives **1a**–**f** and **7a**–**e** are thermally stable substances with a melting point range of 150–224 °C (DSC curves are provided in Supporting Information File 1), and could be used as components of functional organic materials.

## Conclusion

In summary, we have developed a convenient and straightforward approach for the synthesis of previously unknown [1,2,5]oxadiazolo[3,4-*d*][1,2,3]triazin-7(6*H*)-one derivatives based on tandem diazotization/azo coupling reactions of readily available (1,2,5-oxadiazolyl)carboxamides containing an amino functionality. The developed protocol was found to be suitable for the preparation of a library of new biheterocyclic molecules bearing aromatic and aliphatic substituents as well as incorporating amino acid residues. The obtained furoxanotriazinones have demonstrated a moderate NO-releasing ability across a wide range of concentrations under physiological conditions. Moreover, the target bicyclic compounds were shown to be thermostable substances and could be used in various fields of materials science.

## Supporting Information

File 1 Experimental procedures, characterization data of all products, copies of ^1^H, ^13^C NMR, ^15^N spectra of new compounds, DSC curves,X-ray crystallographic data and copies of IR spectra.

## Data Availability

All data that supports the findings of this study is available in the published article and/or the supporting information to this article.
