# Supplementary material for: Tandem diazotization/cyclization approach for the synthesis of a fused 1,2,3-triazinone-furazan/furoxan heterocyclic system
Source: Beilstein J Org Chem. 2024 Sep 16;20:2342–8. doi: 10.3762/bjoc.20.200 (PMC11420545; doi:10.3762/bjoc.20.200)

BEILSTEIN JOURNAL OF ORGANIC CHEMISTRY

Supporting Information

for

Tandem diazotization/cyclization approach for the synthesis of a fused 1,2,3-triazinone-furazan/furoxan heterocyclic system

Yuri A. Sidunets, Valeriya G. Melekhina and Leonid L. Fershtat

Beilstein J. Org. Chem. **2024,** *20,* 2342–2348. doi:10.3762/bjoc.20.200

Experimental procedures, characterization data of all products, copies of ^{1}H, ^{13}C NMR, ^{15}N spectra of new compounds, DSC curves,X-ray crystallographic data and copies of IR spectra

License and Terms: This is a supporting information file under the terms of the Creative Commons Attribution License (https://creativecommons.org/licenses/by/4.0). Please note that the reuse, redistribution and reproduction in particular requires that the author(s) and source are credited and that individual graphics may be subject to special legal provisions.
The license is subject to the *Beilstein Journal of Organic Chemistry* terms and conditions: (https://www.beilstein-journals.org/bjoc/terms)

Table of contents

1. General information........S3
2. Synthesis and characterization data of compounds 2........S3
3. Synthesis and characterization data of compounds 5........S6
4. Synthesis and characterization data of target compounds 1 and 7........S9
5. NO release assay........S13
6. Copies of ^{1}H and ^{13}C NMR spectra for all compounds........S14
6.1 Copies of ^{1}H and ^{13}C NMR spectra for amides 2........S14
6.2 Copies of ^{1}H and ^{13}C NMR spectra for amides 5........S22
6.3 Copies of ^{1}H and ^{13}C NMR spectra for target products 1 and 7........S30
7. ^{15}N NMR spectra for compounds 2a, 1a and 8........S46
8. X-ray crystallographic data and refinement details for compound 1b........S47
9. X-ray crystallographic data and refinement details for compound 7h........S53
10. DSC data........S60
11. Copies of IR spectra........S68

1. General information

All reactions were carried out in well-cleaned oven-dried glassware with magnetic stirring. All solvents were purified and dried using standard methods prior to use. All standard reagents were purchased from Aldrich or Acros Organics and used without further purification. NMR spectra were recorded with Bruker AM 300 (300 MHz) spectrometer in DMSO-d_6. Chemical shifts (ppm) are given relative to solvent signals (DMSO-d_6: 2.50 ppm (^{1}H NMR) and 39.52 ppm (^{13}C NMR)). High-resolution mass spectra (HRMS) were obtained on a Bruker micrOTOF II instrument using electrospray ionization (ESI). The IR spectra were recorded on a Bruker “Alpha” spectrometer in the range 400–4000 cm^{-1} (resolution 2 cm^{-1}). Thermal stability was determined using Netzsch STA F3 apparatus at 5 K min^{-1} heating rate. The extrapolated onset temperatures are given with the indication whether it is melting or thermal decomposition.

2. Synthesis and characterization data of compounds 2

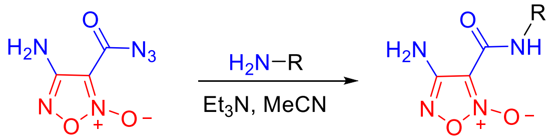

3 **2**

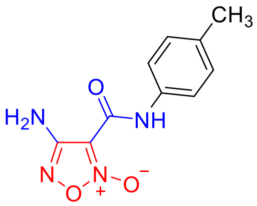

2a (68%)

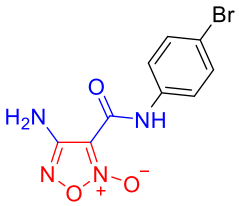

2b (71%)

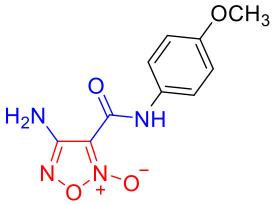

2c (93%)

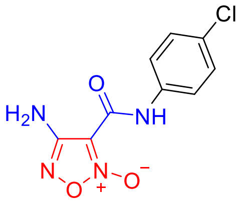

2d (64%)

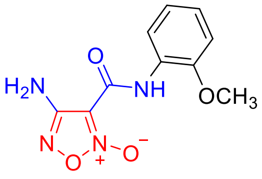

2e (63%)

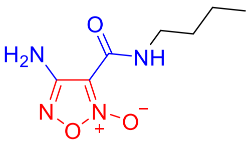

2f (75%)

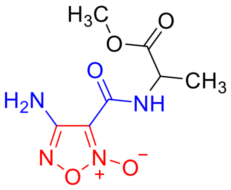

2g (64%)

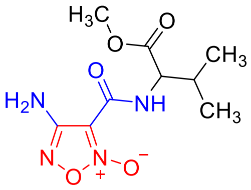

2h (70%)

Experimental procedure for the synthesis of amides 2a–e

A mixture of 4-amino-3-(azidocarbonyl)furoxan **3a-e** (1 mmol), corresponding amine (1.1 mmol), Et_3N (1.1 mmol, 0.11 g) in MeCN (7 mL) was stirred for 3 h. Then reaction mixture poured into 3.5% hydrochloric acid solution (45 mL) and the formed precipitate was filtered off.

4-Amino-3-(p-tolylcarbamoyl)-1,2,5-oxadiazole 2-oxide (2a)

Yield 0.16 g (68%), beige solid, Mp. 210 °C (dec.). IR (KBr): 3441, 3325, 1685, 1624, 1603, 1583, 1547, 1510, 1497, 1201, 996, 816, 754 cm^{-1}. ^{1}H NMR (300 MHz, DMSO-d_6) δ 9.93 (s, 1H), 7.54 (d, *J* = 8.4 Hz, 2H), 7.19 (d, *J* = 8.1 Hz, 2H), 6.62 (s, 2H), 2.28 (s, 3H). ^{13}C NMR (76 MHz, DMSO-d_6) δ 156.9, 153.7, 134.3, 134.2, 129.3, 120.6, 105.6, 20.5. ^{15}N NMR (61 MHz, DMSO-d_6) δ = -34.4, -53.4, -251.5, -330.2. HRMS (ESI-TOF): *m/z*: $[M+Na]^+$ calcd for $C_{10}H_{10}N_4O_3Na$: 257.0645; found: 257.0637.

4-Amino-3-((4-bromophenyl)carbamoyl)-1,2,5-oxadiazole 2-oxide (2b)

Yield 0.21 g (71%), pale pink solid, Mp.214 °C (dec.). IR (KBr): 3456, 3337, 3296, 1675, 1621, 1605, 1578, 1548, 1490, 1205, 1073, 994, 829, 757 cm^{-1}. ^{1}H NMR (300 MHz, DMSO-d_6) δ 10.15 (s, 1H), 7.65 (d, *J* = 9 Hz, 2H), 7.58 (d, *J* = 9 Hz, 2H), 6.62 (s, 2H). ^{13}C NMR (76 MHz, DMSO-d_6) δ 156.8, 154.0, 136.2, 131.7, 122.8, 117.0, 105.6. HRMS (ESI-TOF) *m/z*: $[M+Na]^+$ calcd for $C_9H_7{}^{79}BrN_4O_3Na$: 320.9594; found: 320.9597.

4-Amino-3-((4-methoxyphenyl)carbamoyl)-1,2,5-oxadiazole 2-oxide (2c)

Yield 0.24 g (95%), pale green solid, Mp.186 °C (dec.). IR (KBr): 3412, 3315, 1685, 1617, 1582, 1559, 1507, 1494, 1248, 1235, 1030, 829, 756 cm^{-1}. ^{1}H NMR (300 MHz, DMSO-d_6) δ 9.92 (s, 1H), 7.58 (d, *J* = 9 Hz, 2H), 6.96 (d, *J* = 9 Hz, 2H), 6.61 (s, 2H), 3.75 (s, 3H). ^{13}C NMR (76 MHz, DMSO-d_6) δ 156.9, 156.5, 153.6, 129.7, 122.4, 114.0, 105.5, 55.3. HRMS (ESI-TOF) *m/z*: $[M+Na]^+$ calcd for $C_{10}H_{10}N_4O_4Na$: 273.0594; found: 273.0599.

4-Amino-3-((4-chlorophenyl)carbamoyl)-1,2,5-oxadiazole 2-oxide (2d)

Yield 0.16 g (64%), light orange solid, Mp. 206 °C (dec.). IR (KBr): 3462, 3340, 3296, 1674, 1624, 1607, 1582, 1551, 1492, 1206, 1091, 995, 836, 757 cm^{-1}. ^{1}H NMR (300 MHz, DMSO-d_6) δ 10.16 (s, 1H), 7.69 (d, *J* = 8.9 Hz), 7.45 (d, *J* = 8.8 Hz), 6.61 (s, 2H). ^{13}C NMR (76 MHz, DMSO-d_6) δ 156.8, 154.0, 135.8, 128.8, 122.5, 105.6. HRMS (ESI-TOF) *m/z*: $[M+Na]^+$ calcd for $C_9H_7{}^{35}ClN_4O_3Na$: 277.0099; found: 277.0107.

4-amino-3-((2-methoxyphenyl)carbamoyl)-1,2,5-oxadiazole 2-oxide (2e)

Yield 0.16 g (63%), pink solid, Mp.160 °C. IR (KBr): 3434, 3324, 3298, 1683, 1610, 1582, 1551, 1505, 1256, 1028, 1110, 999, 849, 754 cm^{-1}. ^{1}H NMR (300 MHz, DMSO-d_6) δ 10.12 (s, 1H), 8.28 (d, *J* = 7.4 Hz, 1H), 7.20 – 7.12 (m, 2H), 7.03 – 6.96 (m, 1H), 6.68 (s, 2H), 3.91 (s, 3H). ^{13}C NMR (76 MHz, DMSO-d_6) δ 157.1, 153.6, 148.2, 125.9, 125.2, 120.7, 119.5, 111.2, 105.9, 56.2. HRMS (ESI-TOF) *m/z*: $[M+Na]^+$ calcd for $C_{10}H_{10}N_4O_4Na$: 273.0594; found: 273.0589.

Experimental procedure for the synthesis of amide 2f

A mixture of 4-amino-3-(azidocarbonyl)furoxan **3f** (1 mmol, 0.17 g) and *n*-butylamine (2 mmol, 0.15 g) in MeCN (7 mL) was stirred for 30 min. Then reaction mixture poured into 3.5% hydrochloric acid solution (10 mL) and the formed precipitate was filtered off.

4-Amino-3-(butylcarbamoyl)-1,2,5-oxadiazole 2-oxide (2f)

Yield 0.15 g (75%), white solid, Mp.98 °C. IR (KBr): 3440, 3347, 3318, 3220, 2958, 2928, 2873, 2861, 1679, 1614, 1583, 1568, 1228, 1177, 992, 832, 746 cm^{-1}. ^{1}H NMR (300 MHz, DMSO-d_6) δ 8.29 (t, *J* = 6.0 Hz, 1H), 6.55 (s, 2H), 3.31 - 3.25 (m, 2H), 1.48 (p, *J* = 7.2 Hz, 2H), 1.29 (h, *J* = 7.3 Hz, 2H), 0.87 (t, *J* = 7.3 Hz, 3H). ^{13}C NMR (76 MHz, DMSO-d_6) δ 157.1, 155.6, 104.5, 38.4, 30.8, 19.5, 13.6. HRMS (ESI-TOF) *m/z*: $[M+Na]^+$ calcd for $C_7H_{12}N_4O_3Na$: 223.0802; found: 223.0800.

Experimental procedure for the synthesis of amides 2g,h

A mixture of 4-amino-3-(azidocarbonyl)furoxan **3g,h** (1 mmol), corresponding amine hydrochloride (1.1 mmol), Et_3N (2.2 mmol, 0.22 g) in MeCN (7 mL) was stirred for 4 h. Then reaction mixture poured into H_2O (12 mL) and extracted with EtOAc (3 × 10 mL). The organic layer washed with H_2O and dried over anhydrous $MgSO_4$. The obtained solution was evaporated in *vacuo* and purified by column chromatography on silica gel (*n*-hexane/EtOAc 14:3 → 1:2).

4-Amino-3-((1-methoxy-1-oxopropan-2-yl)carbamoyl)-1,2,5-oxadiazole 2-oxide (2g)

Yield 0.15 g (64%), yellowish solid, Mp. 109 °C. IR (KBr): 3440, 3330, 1740, 1654, 1611, 1574, 1554, 1498, 1451, 1380, 1361, 1322, 1308, 1227, 1210, 1188, 1124, 1006, 859, 759, 750 cm^{-1}. ^{1}H NMR (300 MHz, DMSO-d_6) δ 8.56 (d, *J* = 7.1 Hz, 1H), 6.58 (s, 2H), 4.60-4.51 (m, 1H), 3.68 (s, 3H), 1.41 (d, *J* = 7.1 Hz, 3H). ^{13}C NMR (76 MHz, DMSO-d_6) δ 171.7, 156.9, 155.3, 104.5, 52.3, 47.8, 16.8. HRMS (ESI-TOF) *m/z*: $[M+Na]^+$ calcd for $C_7H_{10}N_4O_5$: 253.0543; found: 253.0550.

4-amino-3-((1-methoxy-3-methyl-1-oxobutan-2-yl)carbamoyl)-1,2,5-oxadiazole 2-oxide (2h)

Yield 0.18 g (70%), yellow-green oil, Mp. 158 °C (dec.). IR (KBr): 3460, 3350, 2966, 2938, 2878, 1743, 1711, 1677, 1616, 1587, 1552, 1372, 1268, 1214, 1185, 1002, 922, 769, 752 cm^{-1}. ^{1}H NMR (300 MHz, DMSO-d_6) δ 8.27 (d, *J* = 8.1 Hz, 1H), 6.58 (s, 2H), 4.45 (dd, *J* = 8.1, 5.3 Hz, 1H), 3.70 (s, 3H), 2.27 – 2.13 (m, 1H), 0.92 (d, *J* = 3.5 Hz, 3H), 0.90 (d, *J* = 3.5 Hz, 3H). ^{13}C NMR (76 MHz, DMSO-d_6) δ 170.7, 156.9, 155.5, 105.0, 56.8, 52.3, 30.3, 18.7, 17.7. HRMS (ESI-TOF) *m/z*: $[M+Na]^+$ calcd for $C_9H_{14}N_4O_5Na$: 281.0856; found: 281.0864.

3. Synthesis and characterization data of compounds 5

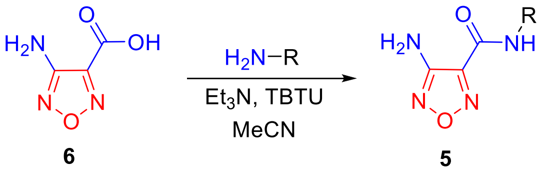

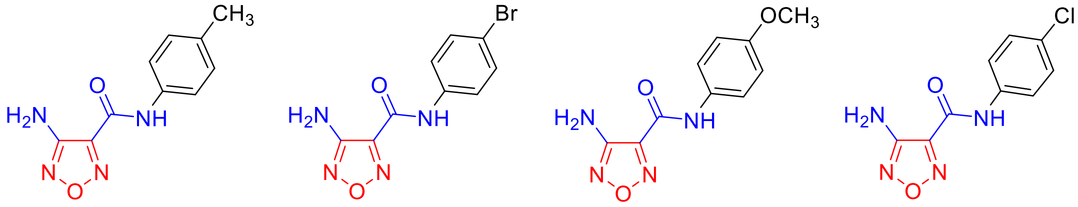

5a (62%) **5b** (65%) **5c** (61%) **5d** (72%)

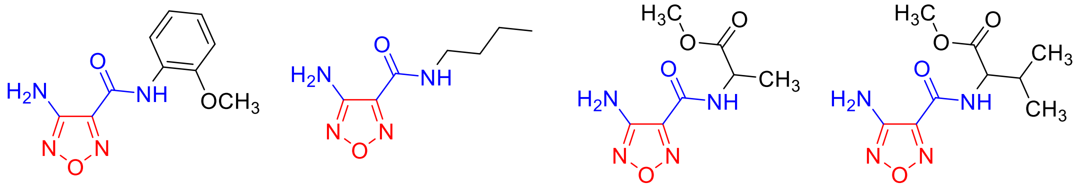

5e (71%) **5f** (35%) **5g** (44%) **5h** (63%)

Experimental procedure for the synthesis of amides 5:

A mixture of 4-amino-3-furazancarboxylic acid **6 (**1 mmol) and 2-(1*H*-benzotriazole-1-yl)-1,1,3,3-tetramethylaminium tetrafluoroborate (TBTU) (1.1. mmol, 0.35 g) in MeCN (6 mL) was stirred 10 min. Then corresponding amine (1.1 mmol) and Et_3N (1.1 mmol, 0.11 g) were added and the reaction mixture was stirred for 2–7 h. The resulting solution was evaporated in vacuo, 3.5% hydrochloric acid solution (10 mL) was added and formed precipitate was filtered off to give the compound **4a–e**. In cases of compounds **4f–h**, solution was extracted with EtOAc (3 × 10 mL), washed with saturated $NaHCO_3$ solution and dried over anhydrous $MgSO_4$. Then solvent was evaporated in vacuo and obtained residue purified by column chromatography on silica gel (*n*-hexane/EtOAc 14:1 → 1:2).

4-Amino-N-(p-tolyl)-1,2,5-oxadiazole-3-carboxamide (5a)

Yield 0.14 g (62%), beige solid, Mp. 181 °C. IR (KBr): 3449, 3394, 3325, 1690, 1618, 1602, 1543, 1512, 1407, 1131, 1008, 816, 601 cm^{-1}. ^{1}H NMR (300 MHz, DMSO-d_6) δ 10.87 (br.s, 1H), 7.64 (d, *J* = 8.5 Hz, 2H), 7.17 (d, *J* = 8.2 Hz, 2H), 6.43 (br.s, 2H), 2.28 (s, 3H). ^{13}C NMR (76 MHz, DMSO-d_6) δ 156.3, 156.2, 141.1, 135.2, 133.8, 129.2, 120.7, 20.5. HRMS (ESI-TOF) *m/z*: $[M+Na]^+$ calcd for $C_{10}H_{10}N_4O_2Na$: 241.0696; found: 241.0704.

4-Amino-N-(4-bromophenyl)-1,2,5-oxadiazole-3-carboxamide (5b)

Yield 0.18 g (65%), yellowish solid, Mp. 193 °C. IR (KBr): 3463, 3358, 3282, 1682, 1621, 1590, 1560, 1530, 1490, 1399, 1239, 1138, 1070, 1001, 897, 809 cm^{-1}. ^{1}H NMR (300 MHz, DMSO-d_6) δ 11.09 (s, 1H), 7.75 (d, *J* = 8.9 Hz, 2H), 7.57 (d, *J* = 8.8 Hz, 2H), 6.43 (s, 2H). ^{13}C NMR (76 MHz, DMSO-d_6) δ 156.6, 156.2, 141.0, 137.1, 131.6, 122.6, 116.5. HRMS (ESI-TOF) *m/z*: $[M+Na]^+$ calcd for $C_9H_7{}^{79}BrN_4O_2Na$: 304.9645; found: 304.9651.

4-Amino-N-(4-methoxyphenyl)-1,2,5-oxadiazole-3-carboxamide (5c)

Yield 0.14 g (61%), purple solid, Mp. 152 °C. IR (KBr): 3468, 3331, 1666, 1642, 1618, 1559, 1507, 1413, 1304, 1252, 1034, 889, 827, 719 cm^{-1}. ^{1}H NMR (300 MHz, DMSO-d_6) δ 10.85 (s, 1H), 7.67 (d, *J* = 9.0 Hz, 2H), 6.94 (d, *J* = 9.0 Hz, 2H), 6.42 (s, 2H), 3.75 (s, 3H). ^{13}C NMR (76 MHz, DMSO-d_6) δ 156.2, 156.2, 156.1, 141.1, 130.7, 122.3, 113.9, 55.2. HRMS (ESI-TOF) *m/z*: $[M+Na]^+$ calcd for $C_{10}H_{10}N_4O_3Na$: 257.0645; found: 257.0653.

4-Amino-N-(4-chlorophenyl)-1,2,5-oxadiazole-3-carboxamide (5d)

Yield 0.17 g (72%), yellowish solid, Mp. 191 °C. IR (KBr): 3470, 3367, 3284, 1683, 1623, 1596, 1560, 1539, 1494, 1404, 1240, 1140, 1090, 1001, 897, 813 cm^{-1}. ^{1}H NMR (300 MHz, DMSO-d_6) δ 11.10 (s, 1H), 7.80 (d, *J* = 8.8 Hz, 2H), 7.43 (d, *J* = 8.9 Hz, 2H), 6.45 (s, 2H). ^{13}C NMR (76 MHz, DMSO-d_6) δ 156.6, 156.2, 140.9, 136.7, 128.7, 128.4, 122.3. HRMS (ESI-TOF) *m/z*: $[M+Na]^+$ calcd for $C_9H_7{}^{35}ClN_4O_2Na$: 261.0150; found: 261.0146.

4-Amino-N-(2-methoxyphenyl)-1,2,5-oxadiazole-3-carboxamide (5e)

Yield 0.17 g (71%), dark beige solid, Mp. 141 °C. IR (KBr): 3446, 3387, 3323, 1687, 1625, 1606, 1542, 1513, 1462, 1250, 1138, 1019, 888, 744 cm^{-1}. ^{1}H NMR (300 MHz, DMSO-d_6) δ 9.76 (s, 1H), 7.87 (d, *J* = 7.8 Hz, 1H), 7.22 (td, *J* = 7.9, 7.4, 1.7 Hz, 1H), 7.13 (dd, *J* = 8.3, 1.4 Hz, 1H), 6.99 (td, *J* = 7.6, 1.4 Hz, 1H), 6.42 (s, 2H), 3.86 (s, 3H). ^{13}C NMR (76 MHz, DMSO-d_6) δ 156.2, 156.1, 150.7, 140.7, 126.3, 125.4, 123.0, 120.4, 111.5, 55.9. HRMS (ESI-TOF) *m/z*: $[M+Na]^+$ calcd for $C_{10}H_{10}N_4O_3Na$: 257.0645; found: 257.0651.

4-amino-N-butyl-1,2,5-oxadiazole-3-carboxamide (5f)

Yield 0.06 g (35%), yellowish solid, Mp. 69 °C. IR (KBr): 3459, 3342, 2956, 2928, 2862, 1650, 1623, 1572, 1508, 1427, 1337, 1197, 1149, 1005, 839, 787 cm^{-1}.^{1}H NMR (300 MHz, DMSO-d_6) δ 9.03 (t, *J* = 5.0 Hz, 1H), 6.32 (s, 2H), 3.28 – 3.20 (m, 2H), 1.50 (p, *J* = 7.4 Hz, 2H), 1.38 – 1.23

(m, 2H), 0.89 (t, *J* = 7.3 Hz, 3H). ^{13}C NMR (76 MHz, DMSO-d_6) δ 157.8, 156.1, 140.5, 38.5, 30.8, 19.5, 13.6. HRMS (ESI-TOF) *m/z*: $[M+Na]^+$ calcd for $C_7H_{12}N_4O_2Na$: 207.0852; found: 207.0858.

Methyl (4-amino-1,2,5-oxadiazole-3-carbonyl)alaninate (5g)

Yield 0.09 g (44%), yellowish solid, Mp. 91 °C. IR (KBr): 3436, 3340, 3236, 2961, 1735, 1677, 1632, 1545, 1505, 1460, 1435, 1360, 1229, 1196, 1181, 1009, 976, 861, 840 cm^{-1}. ^{1}H NMR (300 MHz, DMSO-d_6) δ 9.48 (d, *J* = 7.1 Hz, 1H), 6.36 (s, 2H), 4.50 (p, *J* = 7.2 Hz, 1H), 3.66 (s, 3H), 1.40 (d, *J* = 7.3 Hz, 3H). ^{13}C NMR (76 MHz, DMSO-d_6) δ 172.2, 157.9, 156.1, 140.0, 52.1, 47.9, 16.4. HRMS (ESI-TOF) *m/z*: $[M+Na]^+$ calcd for $C_7H_{10}N_4O_4Na$: 237.0594; found: 237.0599.

Methyl (4-amino-1,2,5-oxadiazole-3-carbonyl)valinate (5h)

Yield 0.15 g (63%), white solid, Mp. 85 °C. IR (KBr): 3451, 3434, 3349, 3324, 2963, 2930, 1727, 1669, 1629, 1619, 1563, 1464, 1431, 1296, 1278, 1251, 1198, 1176, 1008, 905, 805 cm^{-1}. ^{1}H NMR (300 MHz, DMSO-d_6) δ 9.30 (d, *J* = 7.7 Hz, 1H), 6.33 (s, 2H), 4.28 (t, *J* = 7.5 Hz, 1H), 3.67 (s, 3H), 2.27-2.15 (m, 1H), 0.95 (d, *J* = 6.7 Hz, 3H), 0.92 (d, *J* = 6.8 Hz, 3H). ^{13}C NMR (76 MHz, DMSO-d_6) δ 171.2, 158.4, 156.0, 140.2, 58.2, 51.9, 29.3, 19.0, 18.9. HRMS (ESI-TOF) *m/z*: $[M+Na]^+$ calcd for $C_9H_{14}N_4O_4Na$: 265.0907; found: 265.0911.

4. Synthesis and characterization data of target compounds 1 and 7

General experimental procedure:

Amide **2** or **5** (1 mmol) was dissolved in mixture of AcOH/$MeSO_3H$ [1:1] (3 mL), cooled to −10–0 °C and $NaNO_2$ (1.05 mmol, 0.072 g) was added. The reaction mixture was kept for 1 h, then a cooling bath was removed and the mixture was stirred for 40–150 min.

Purifications:

Compounds **1a,b,d,f** and **7c**: water (10 mL) was added to the mixture, the formed precipitate was filtered off, and obtained residue was recrystallized from MeCN/H_2O [1:1].

Compounds **1c** and **7a,b,d**: water (10 mL) was added to the mixture, the formed precipitate was filtered off and washed with water.

Compounds **1e** and **7e**: water (10 mL) was added to the mixture, the formed precipitate was filtered off and washed with water. Then resulting residue was purified by column chromatography on silica gel (*n*-hexane/EtOAc).

Compounds **1g,h** and **7f,g,h**: water (20 mL) was added to the mixture and the solution was extracted with EtOAc (3 × 10 mL) and dried over anhydrous $MgSO_4$. Then solvent was evaporated in vacuo and obtained residue purified by column chromatography on silica gel (*n*-hexane/EtOAc).

7-Oxo-6-(p-tolyl)-6,7-dihydro-[1,2,5]oxadiazolo[3,4-d][1,2,3]triazine 1-oxide (1a)

Yield 0.22 g (89%), yellow solid, Mp. 224 °C (dec.). IR (KBr): 1731, 1641, 1508, 1465, 1285, 1167, 1101, 1036, 1019, 1004, 940, 814, 754 cm^{-1}. ^1H NMR (300 MHz, DMSO-d_6) δ 7.41 (s, 4H), 2.41 (s, 3H). ^{13}C NMR (76 MHz, DMSO-d_6) δ 155.6, 147.2, 139.7, 134.5, 129.9, 126.3, 100.4, 40.4, 40.1, 39.8, 39.5, 39.2, 39.0, 38.7, 20.8. ^{15}N NMR (51 MHz, DMSO-d_6) δ = 51.0, -5.1, -37.1, -138.4, -232.6.HRMS (ESI-TOF) *m/z*: $[M+CH_3OH+H]^+$ calcd for $C_{10}H_7N_5O_3CH_3OH_2$: 278.0884; found: 278.0882.

6-(4-Bromophenyl)-7-oxo-6,7-dihydro-[1,2,5]oxadiazolo[3,4-d][1,2,3]triazine 1-oxide (1b)

Yield 0.27 g (87%), yellow solid, Mp. 189 °C (dec.). IR (KBr): 1744, 1721, 1651, 1487, 1470, 1284, 1106, 1024, 1016, 998, 939, 819, 755 cm^{-1}. ^1H NMR (300 MHz, DMSO-d_6) δ 7.85 (d, *J* = 8.7 Hz, 2H), 7.50 (d, *J* = 8.7 Hz, 2H). ^{13}C NMR (76 MHz, DMSO-d_6) δ 155.4, 147.1, 136.1, 132.5, 128.6, 123.1, 100.3. HRMS (ESI-TOF) *m/z*: $[M+CH_3OH+Na]^+$ calcd for $C_9H_4{}^{79}BrN_5O_3CH_3OHNa$: 363.9652; found: 363.9645.

6-(4-Methoxyphenyl)-7-oxo-6,7-dihydro-[1,2,5]oxadiazolo[3,4-d][1,2,3]triazine 1-oxide (1c)

Yield 0.23 g (87%), green-yellow solid, Mp. 211 °C (dec.). IR (KBr): 1731, 1641, 1610, 1514, 1460, 1285, 1260, 1170, 1106, 1026, 1009, 940, 820, 754 cm^{-1}. ^1H NMR (300 MHz, DMSO-d_6) δ 7.44 (d, *J* = 9.0 Hz, 2H), 7.15 (d, *J* = 8.9 Hz, 2H), 3.84 (s, 3H). ^{13}C NMR (76 MHz, DMSO-d_6) δ 160.0, 155.6, 147.2, 129.6, 127.9, 114.6, 100.4, 55.6. HRMS (ESI-TOF) *m/z*: $[M+CH_3OH+Na]^+$ calcd for $C_{10}H_7N_5O_4CH_3OHNa$: 316.0652; found: 316.0656.

6-(4-Chlorophenyl)-7-oxo-6,7-dihydro-[1,2,5]oxadiazolo[3,4-d][1,2,3]triazine 1-oxide (1d)

Yield 0.24 g (89%), yellow solid, Mp. 196 °C (dec.). IR (KBr): 1723, 1654, 1488, 1464, 1288, 1107, 1027, 1000, 940, 824, 756 cm^{-1}. ^{1}H NMR (300 MHz, DMSO-d_6) δ 7.71 (d, *J* = 8.7 Hz, 2H), 7.56 (d, *J* = 8.8 Hz, 2H). ^{13}C NMR (76 MHz, DMSO-d_6) δ 155.4, 147.1, 135.7, 134.5, 129.6, 128.4, 100.3. HRMS (ESI-TOF) *m/z*: $[M+CH_3OH+Na]^+$ calcd for $C_9H_4{}^{35}ClN_5O_3CH_3OHNa$: 320.0157; found: 320.0164.

6-(2-Methoxyphenyl)-7-oxo-6,7-dihydro-[1,2,5]oxadiazolo[3,4-d][1,2,3]triazine 1-oxide (1e)

Yield 0.16 g (63%), yellow solid, Mp. 174 °C (dec.). IR (KBr): 1750, 1652, 1638, 1600, 1499, 1462, 1301, 1282, 1246, 1094, 1040, 1024, 998, 938, 757 cm^{-1}. ^{1}H NMR (300 MHz, DMSO-d_6) δ 7.64 – 7.55 (m, 1H), 7.42 (dd, *J* = 7.8, 1.7 Hz, 1H), 7.32 (dd, *J* = 8.5, 1.2 Hz, 1H), 7.17 (td, *J* = 7.6, 1.2 Hz, 1H), 3.81 (s, 3H). ^{13}C NMR (76 MHz, DMSO-d_6) δ 155.4, 154.6, 146.5, 132.1, 129.0, 125.2, 120.9, 112.9, 100.4, 56.0. HRMS (ESI-TOF) *m/z*: $[M+Na]^+$ calcd for $C_{10}H_7N_5O_4Na$: 284.0390; found: 284.0394.

6-Butyl-7-oxo-6,7-dihydro-[1,2,5]oxadiazolo[3,4-d][1,2,3]triazine 1-oxide (1f)

Yield 0.16 g (77%), yellowish solid, Mp. 150 °C. IR (KBr): 2961, 1727, 1653, 1471, 1318, 1254, 1234, 1040, 1012, 979, 920, 758 cm^{-1}. ^{1}H NMR (300 MHz, DMSO-d_6) δ 4.30 (t, *J* = 7.0 Hz, 2H), 1.73 (p, *J* = 7.3 Hz, 2H), 1.37 (h, *J* = 7.3 Hz, 2H), 0.92 (t, *J* = 7.3 Hz, 3H). ^{13}C NMR (76 MHz, DMSO-d_6) δ 155.9, 147.2, 100.3, 48.9, 30.2, 19.0, 13.4. HRMS (ESI-TOF) *m/z*: $[M+CH_3OH+Na]^+$ calcd for $C_7H_9N_5O_3CH_3OHNa$: 266.0860; found: 266.0871.

6-(1-Methoxy-1-oxopropan-2-yl)-7-oxo-6,7-dihydro-[1,2,5]oxadiazolo[3,4-d][1,2,3]triazine 1-oxide (1g)

Yield 0.11 g (45%), yellowish solid, Mp. 119 °C. IR (KBr): 2966, 1749, 1734, 1668, 1640, 1473, 1459, 1301, 1244, 1102, 1060, 981, 963, 758 cm^{-1}. ^{1}H NMR (300 MHz, DMSO-d_6) δ 5.78 (q, *J* = 7.1 Hz, 1H), 3.71 (s, 3H), 1.65 (d, *J* = 7.2 Hz, 3H). ^{13}C NMR (76 MHz, DMSO-d_6) δ 169.1, 155.4, 146.7, 100.1, 56.4, 52.8, 15.6. HRMS (ESI-TOF) *m/z*: $[M+CH_3OH+Na]^+$ calcd for $C_7H_7N_5O_5CH_3OHNa$: 296.0602; found: 296.0592.

6-(1-Methoxy-3-methyl-1-oxobutan-2-yl)-7-oxo-6,7-dihydro-[1,2,5]oxadiazolo[3,4-d][1,2,3]triazine 1-oxide (1h)

Yield 0.18 g (66%), yellow solid, Mp. 103 °C. IR (KBr): 2975, 2958, 1744, 1659, 1639, 1468, 1293, 1215, 1148, 1042, 1011, 984, 775, 754 cm^{-1}. ^{1}H NMR (300 MHz, DMSO-d_6) δ 5.37 (d, *J* = 8.2 Hz, 1H), 3.69 (s, 3H), 2.72 – 2.58 (m, 1H), 1.07 (d, *J* = 6.7 Hz, 3H), 0.92 (d, *J* = 6.7 Hz, 3H). ^{13}C NMR (76 MHz, DMSO-d_6) δ 168.0, 155.1, 147.3, 100.2, 64.2, 52.6, 29.4, 19.3, 18.6.HRMS (ESI-TOF) *m/z*: $[M+Na]^+$ calcd for $C_9H_{11}N_5O_5Na$: 292.0652; found: 292.0639.

6-(p-Tolyl)-[1,2,5]oxadiazolo[3,4-d][1,2,3]triazin-7(6H)-one (7a)

Yield 0.23 g (93%), light orange solid, Mp. 211 °C (dec.). IR (KBr): 1770, 1735, 1697, 1511, 1464, 1296, 1232, 1113, 1099, 1016, 954, 814, 601 cm^{-1}. ^{1}H NMR (300 MHz, DMSO-d_6) δ 7.45 (s, 4H), 2.43 (s, 3H). ^{13}C NMR (76 MHz, DMSO-d_6) δ 157.7, 148.9, 141.0, 139.6, 135.0, 129.8, 126.5, 20.8. HRMS (ESI-TOF) *m/z*: $[M+CH_3OH+Na]^+$ calcd for $C_{10}H_7N_5O_2CH_3OHNa$: 284.0754; found: 284.0741.

6-(4-Bromophenyl)-[1,2,5]oxadiazolo[3,4-d][1,2,3]triazin-7(6H)-one (7b)

Yield 0.29 g (95%), beige solid, Mp. 221 °C (dec.). IR (KBr): 1762, 1741, 1489, 1468, 1301, 1236, 1101, 1029, 1011, 953, 826, 780 cm^{-1}. ^{1}H NMR (300 MHz, DMSO-d_6) δ 7.87 (d, *J* = 8.7 Hz, 2H), 7.54 (d, *J* = 8.7 Hz, 2H). ^{13}C NMR (76 MHz, DMSO-d_6) δ 157.6, 148.7, 140.9, 136.6, 132.5, 128.8, 123.0. HRMS (ESI-TOF) *m/z*: $[M+CH_3OH+H]^+$ calcd for $C_9H_4{}^{79}BrN_5O_2CH_3OH_2$: 325.9883; found: 325.9875.

6-(4-Methoxyphenyl)-[1,2,5]oxadiazolo[3,4-d][1,2,3]triazin-7(6H)-one (7c)

Yield 0.22 g (88%), green-yellow solid, Mp. 206 °C (dec.). IR (KBr): 1735, 1610, 1514, 1461, 1313, 1295, 1263, 1172, 1101, 1033, 954, 837, 822 cm^{-1}. ^{1}H NMR (300 MHz, DMSO-d_6) δ 7.49 (d, *J* = 8.6 Hz, 2H), 7.18 (d, *J* = 8.6 Hz, 2H), 3.86 (s, 3H). ^{13}C NMR (76 MHz, DMSO-d_6) δ 160.0, 157.6, 149.0, 141.0, 130.1, 128.1, 114.5, 55.6. HRMS (ESI-TOF) *m/z*: $[M+Na]^+$ calcd for $C_{10}H_7N_5O_3Na$: 268.0441; found: 268.0446.

6-(4-Chlorophenyl)-[1,2,5]oxadiazolo[3,4-d][1,2,3]triazin-7(6H)-one (7d)

Yield 0.2g (80%), beige solid, Mp. 212 °C.IR (KBr): 1764, 1734, 1697, 1491, 1470, 1301, 1236, 1103, 1032, 1014, 955, 833, 783 cm^{-1}. ^{1}H NMR (300 MHz, DMSO-d_6) δ 7.73 (d, *J* = 8.6 Hz, 2H), 7.60 (d, *J* = 8.6 Hz, 2H). ^{13}C NMR (76 MHz, DMSO-d_6) δ 157.6, 148.8, 140.9, 136.2, 134.5, 129.5, 128.6. HRMS (ESI-TOF) *m/z*: $[M+CH_3OH+Na]^+$ calcd for $C_9H_4{}^{35}ClN_5O_2CH_3OHNa$: 304.0208; found: 304.0215.

6-(2-Methoxyphenyl)-[1,2,5]oxadiazolo[3,4-d][1,2,3]triazin-7(6H)-one (7e)

Yield 0.15 g (63%), orange solid, Mp. 156 °C. IR (KBr): 1746, 1599, 1499, 1456, 1304, 1284, 1255, 1088, 1043, 1022, 952, 754 cm^{-1}. ^{1}H NMR (300 MHz, DMSO-d_6) δ 7.61 (td, *J* = 8.1, 7.6, 1.8 Hz, 1H), 7.47 (dd, *J* = 7.8, 1.7 Hz, 1H), 7.33 (d, *J* = 8.4 Hz, 1H), 7.19 (td, *J* = 7.6, 1.2 Hz, 1H), 3.82 (s, 3H). ^{13}C NMR (76 MHz, DMSO-d_6) δ 157.7, 154.5, 148.1, 140.5, 132.0, 129.0, 125.7, 120.8, 112.9, 56.0. HRMS (ESI-TOF) *m/z*: $[M+CH_3OH+Na]^+$ calcd for $C_{10}H_7N_5O_3CH_3OHNa$: 300.0703; found: 300.0699.

6-Butyl-[1,2,5]oxadiazolo[3,4-d][1,2,3]triazin-7(6H)-one (7f)

Yield 0.14 g (70%), yellowish solid, Mp. 104 °C. IR (KBr): 2960, 2934, 2875, 1734, 1575, 1467, 1277, 1172, 1060, 1018, 922, 826 cm^{-1}. ^{1}H NMR (300 MHz, DMSO-d_6) δ 4.38 (t, *J* = 7.1 Hz, 2H), 1.79 (p, *J* = 7.3 Hz, 2H), 1.41 (h, *J* = 7.3 Hz, 2H), 0.93 (t, *J* = 7.3 Hz, 3H). ^{13}C NMR (76 MHz,

DMSO-d_6) δ 157.8, 149.0, 140.2, 49.1, 30.2, 19.1, 13.5. HRMS (ESI-TOF) *m/z*: $[M+CH_3OH+Na]^+$ calcd for $C_7H_9N_5O_2CH_3OHNa$: 250.0911; found: 250.0911.

Methyl 2-(7-oxo-[1,2,5]oxadiazolo[3,4-d][1,2,3]triazin-6(7H)-yl)propanoate (7g)

Yield 0.08 g (34%), yellow-green oil, Mp. >150 °C (evap.). IR (KBr): 3005, 2958, 1730, 1575, 1461, 1439, 1345, 1304, 1275, 1241, 1205, 1148, 1098, 1068, 994, 964, 811, 785 cm^{-1}. ^1H NMR (300 MHz, DMSO-d_6) δ 5.88 (q, J = 7.1 Hz, 1H), 3.71 (s, 3H), 1.68 (d, J = 7.1 Hz, 3H). ^{13}C NMR (76 MHz, DMSO-d_6) δ 169.2, 157.6, 148.4, 140.0, 56.6, 52.8, 15.7. HRMS (ESI-TOF) *m/z*: $[M+CH_3OH+Na]^+$ calcd for $C_7H_7N_5O_4CH_3OHNa$: 280.0652; found: 296.0660.

Methyl 3-methyl-2-(7-oxo-[1,2,5]oxadiazolo[3,4-d][1,2,3]triazin-6(7H)-yl)butanoate (7h)

Yield 0.14 g (57%), white solid, Mp. 123 °C. IR (KBr): 2988, 2974, 2962, 2925, 1741, 1575, 1469, 1438, 1338, 1275, 1217, 1140, 1066, 1039, 1006, 989, 935, 899, 788 cm^{-1}. ^1H NMR (300 MHz, DMSO-d_6) δ 5.46 (d, J = 8.3 Hz, 1H), 3.69 (s, 3H), 2.80 – 2.61 (m, 1H), 1.09 (d, J = 6.7 Hz, 3H), 0.95 (d, J = 6.7 Hz, 3H). ^{13}C NMR (76 MHz, DMSO-d_6) δ 168.0, 157.3, 149.0, 139.9, 64.4, 52.5, 29.5, 19.2, 18.7. HRMS (ESI-TOF) *m/z*: $[M+CH_3OH+Na]^+$ calcd for $C_9H_{11}N_5O_4CH_3OHNa$: 308.0965; found: 308.0970.

5. NO release assay

The test molecules **1** were dissolved in DMSO to obtain a solution with a resulting concentration 2 mM. 20 µL aliquots of the resulted solutions were diluted with phosphate buffer solution (165 µL, pH 7.4, containing 4.6 mM L-cysteine). The final concentration of the furoxan derivatives **1** was 0.2 mM. The mixtures were incubated at 37 °C for 1 h. 5 µL aliquot of the Griess reagent (prepared by mixing sulfanilamide (0.12 g), *N*-naphthylethylenediamine dihydrochloride (0.006 g) and 85% H_3PO_4 (2.7 mL) in distilled and deionized water (final volume 3 mL)) was added to the incubated furoxan solutions and incubated for another 10 min at 37 °C. UV absorbance at 540 nm was measured using a Multiskan GO Microplate Photometer and calibrated using a standard curve prepared from standard solution of $NaNO_2$ to give the nitrite concentration. All measurements were made in triplicate. No significant NO release was detected at the absence of L-cysteine.

6. Copies of ^1H and ^{13}C NMR spectra for all compounds

6.1 Copies of ^1H and ^{13}C NMR spectra for amides 2

^1H NMR spectrum (300 MHz) of **2a** in DMSO-d_6

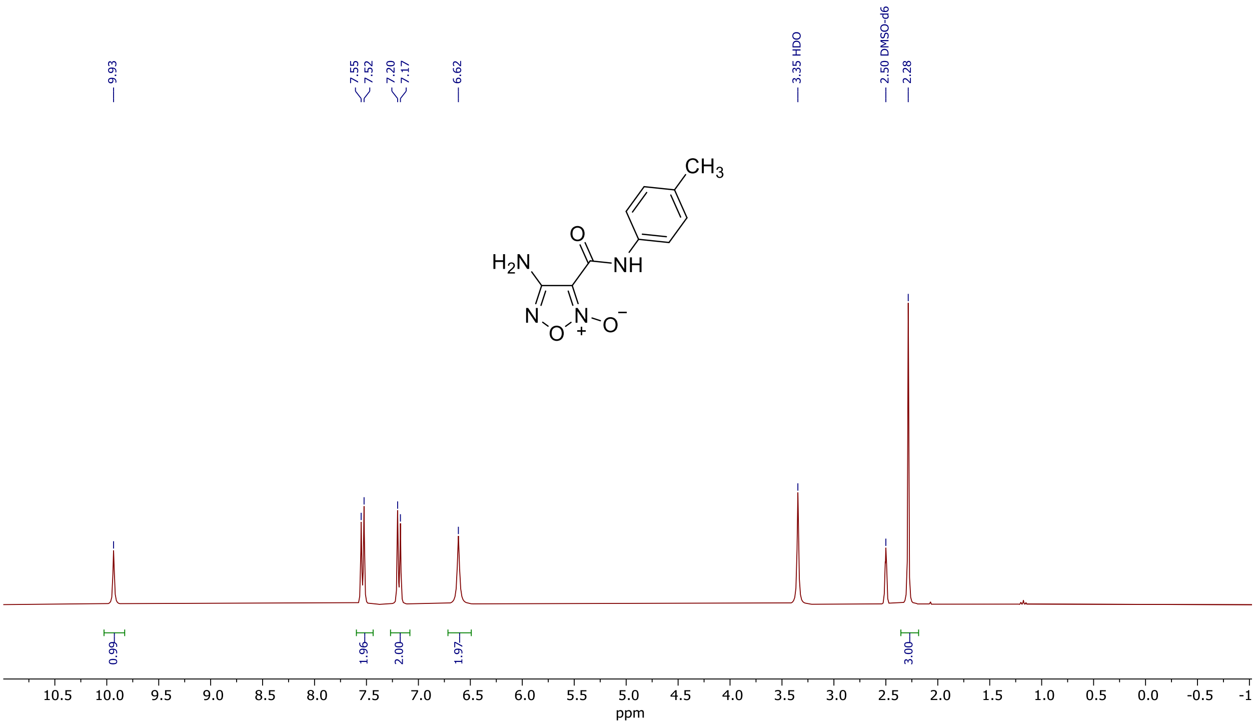

^{13}C {^1H} NMR spectrum (76 MHz) of **2a** in DMSO-d_6

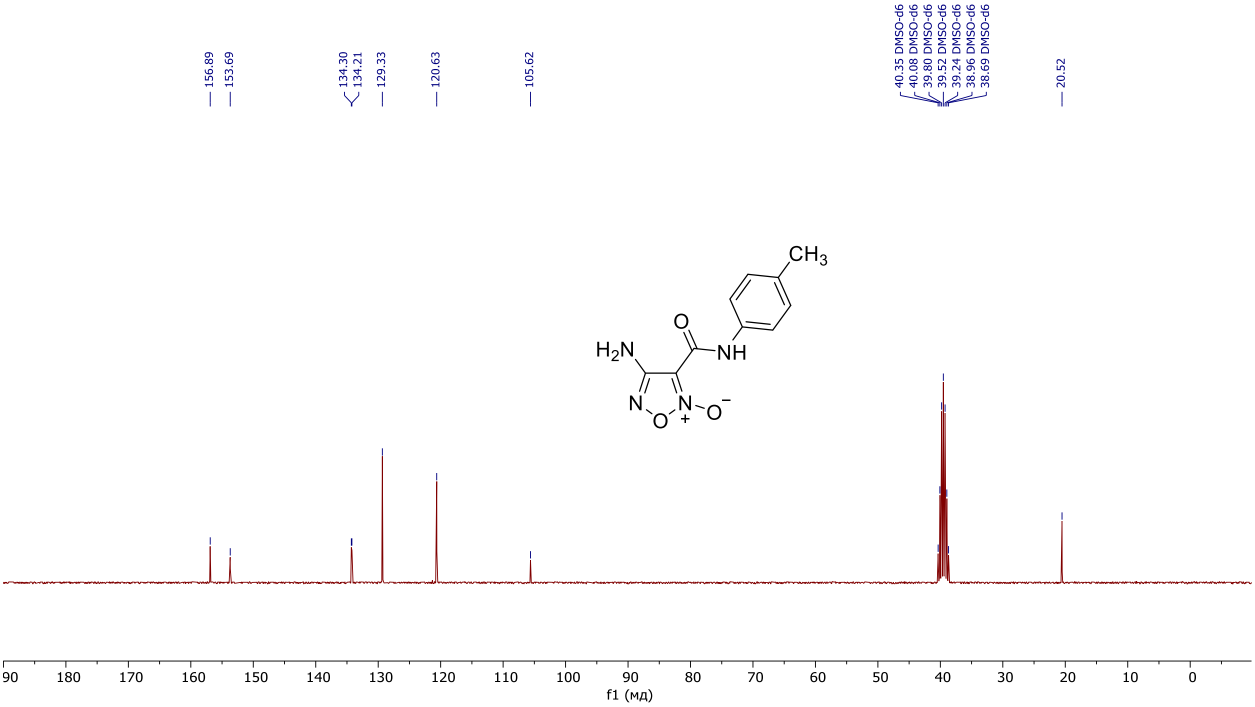

^{1}H NMR spectrum (300 MHz) of **2b** in DMSO-d_6

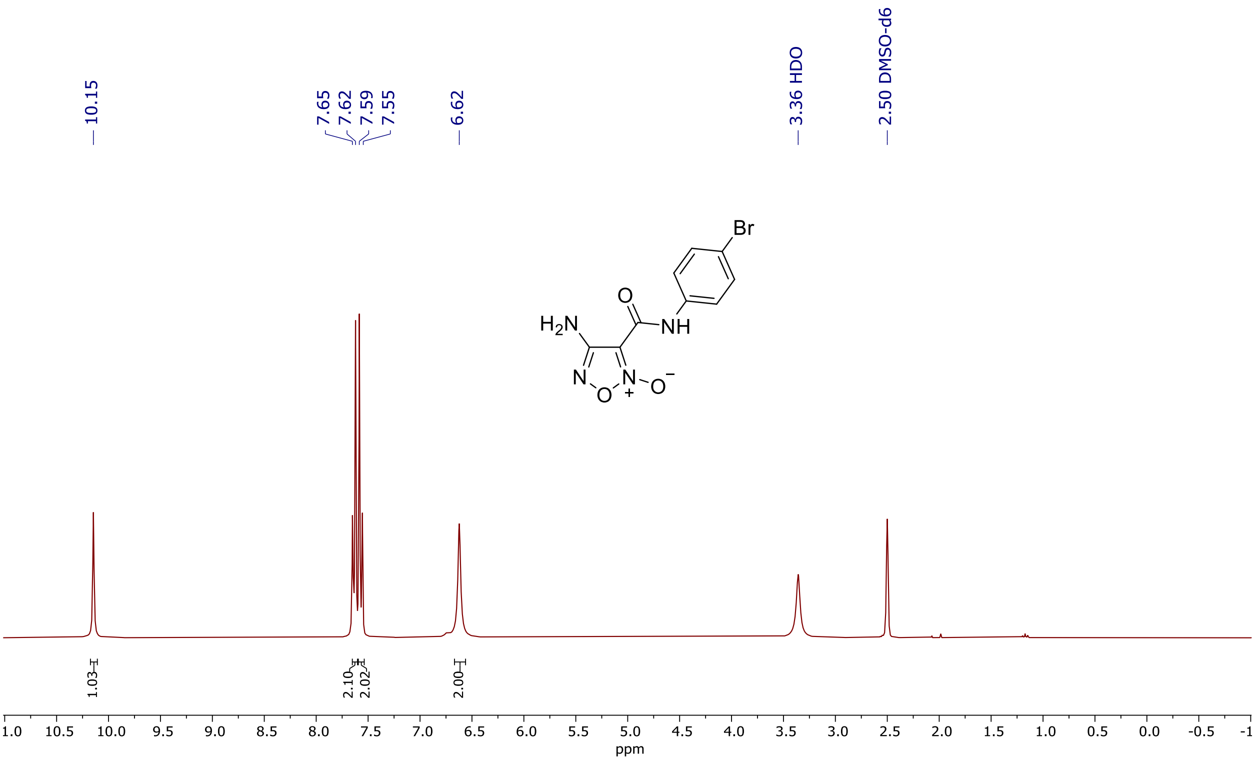

^{13}C {^{1}H} NMR spectrum (76 MHz) of **2b** in DMSO-d_6

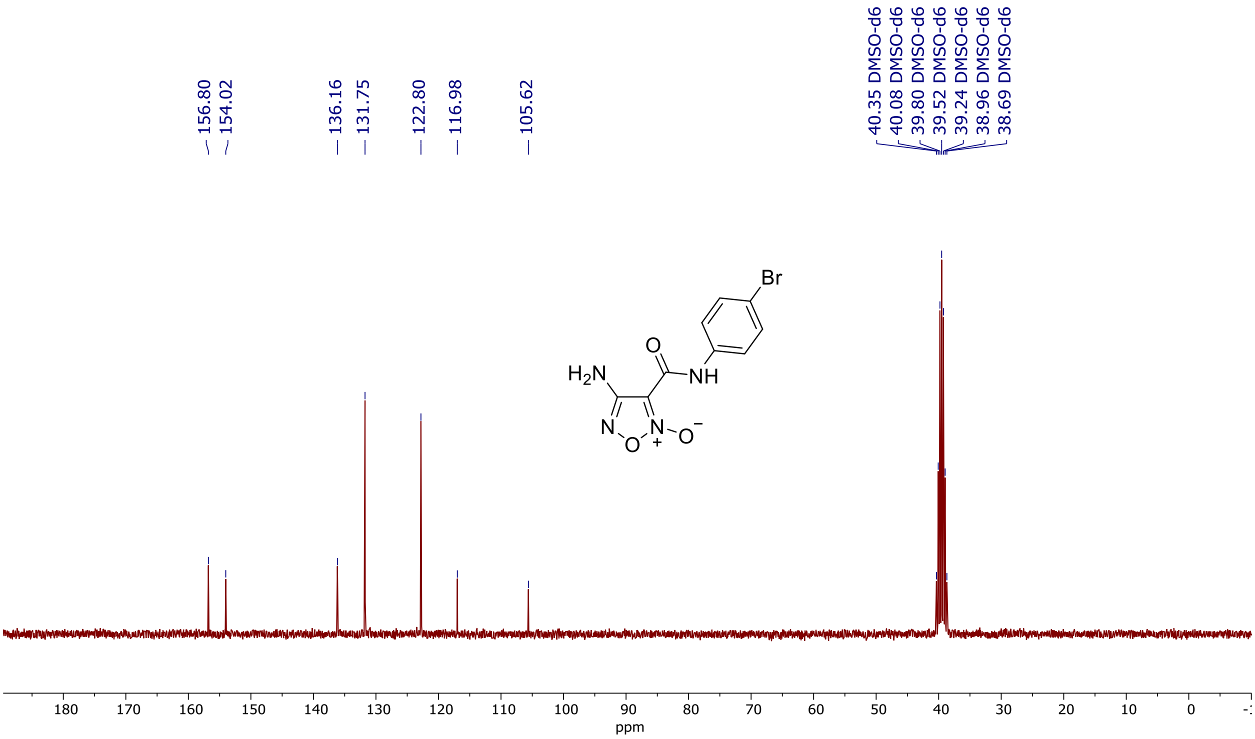

^{1}H NMR spectrum (300 MHz) of **2c** in DMSO-d_6

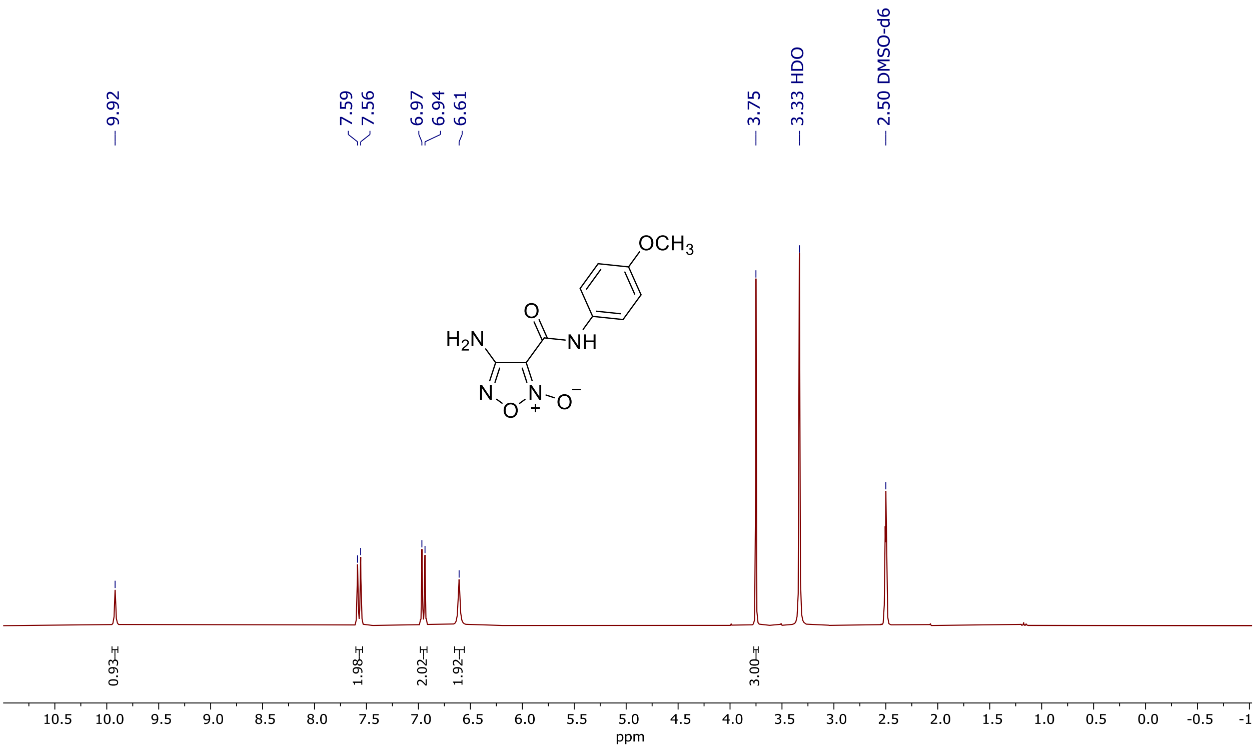

^{13}C {^{1}H} NMR spectrum (76 MHz) of **2c** in DMSO-d_6

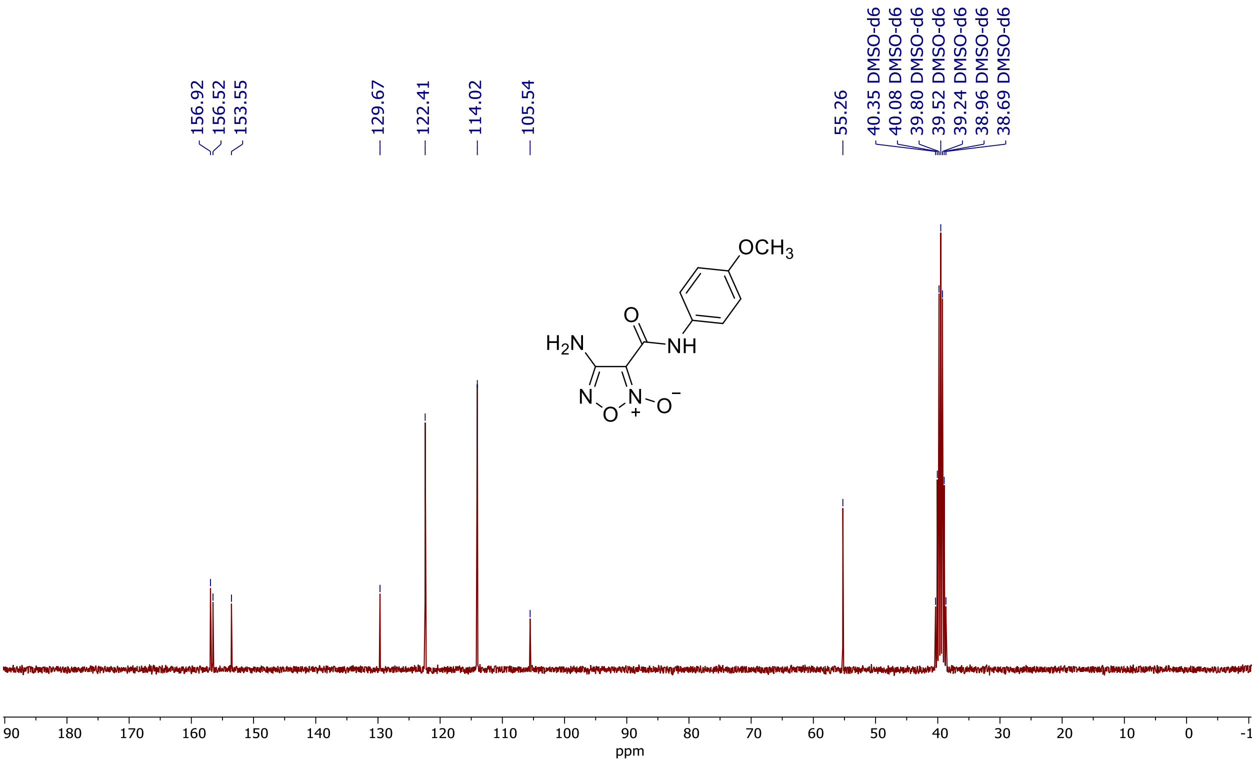

^{1}H NMR spectrum (300 MHz) of **2d** in DMSO-d_6

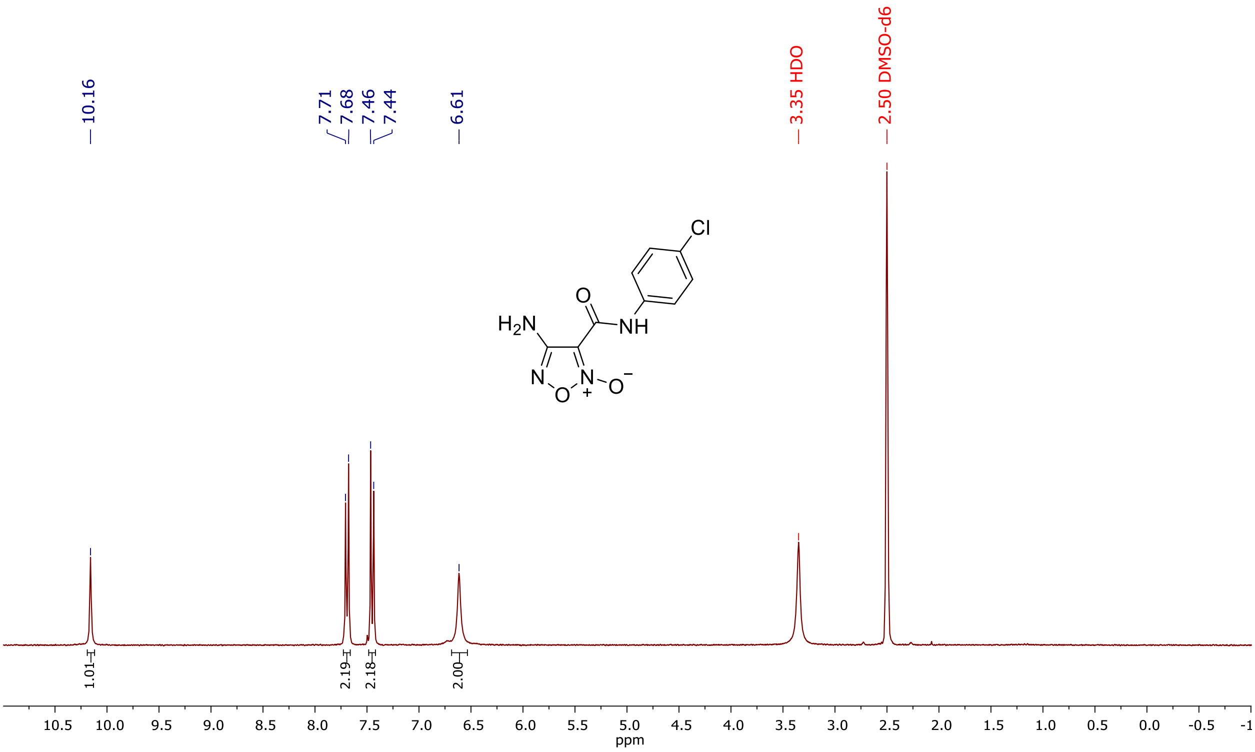

^{13}C {^{1}H} NMR spectrum (76 MHz) of **2d** in DMSO-d_6

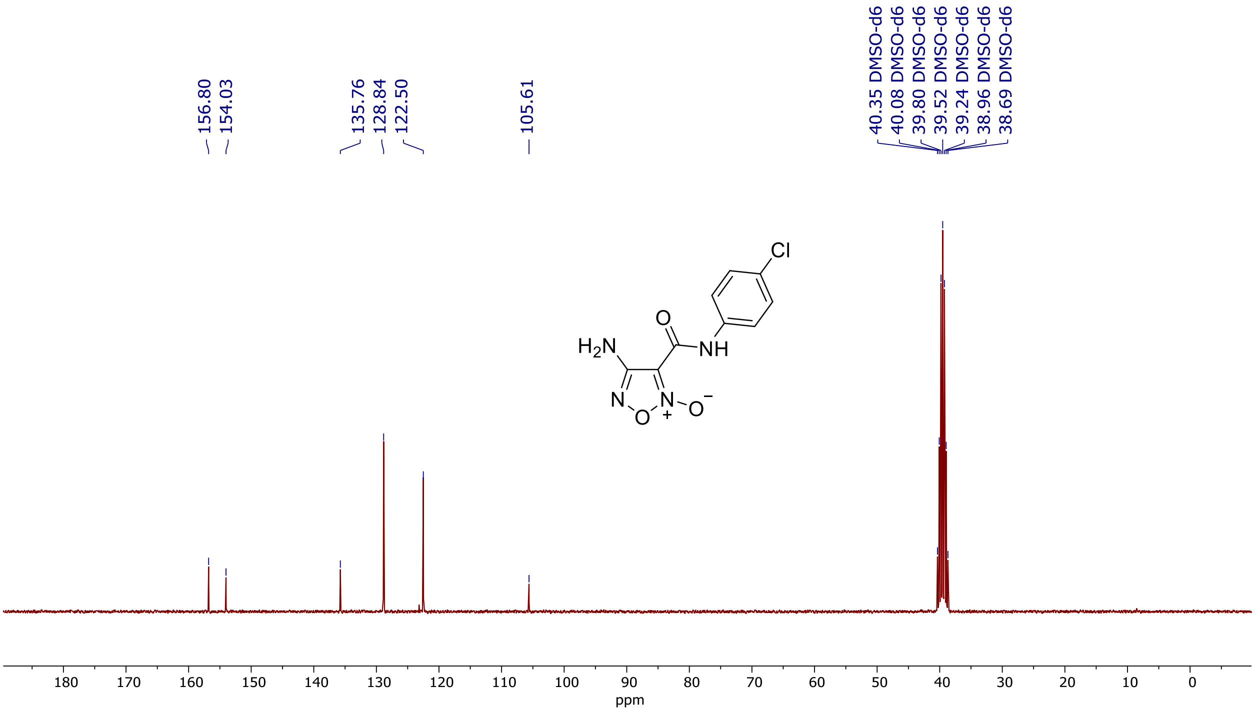

^{1}H NMR spectrum (300 MHz) of **2e** in DMSO-d_6

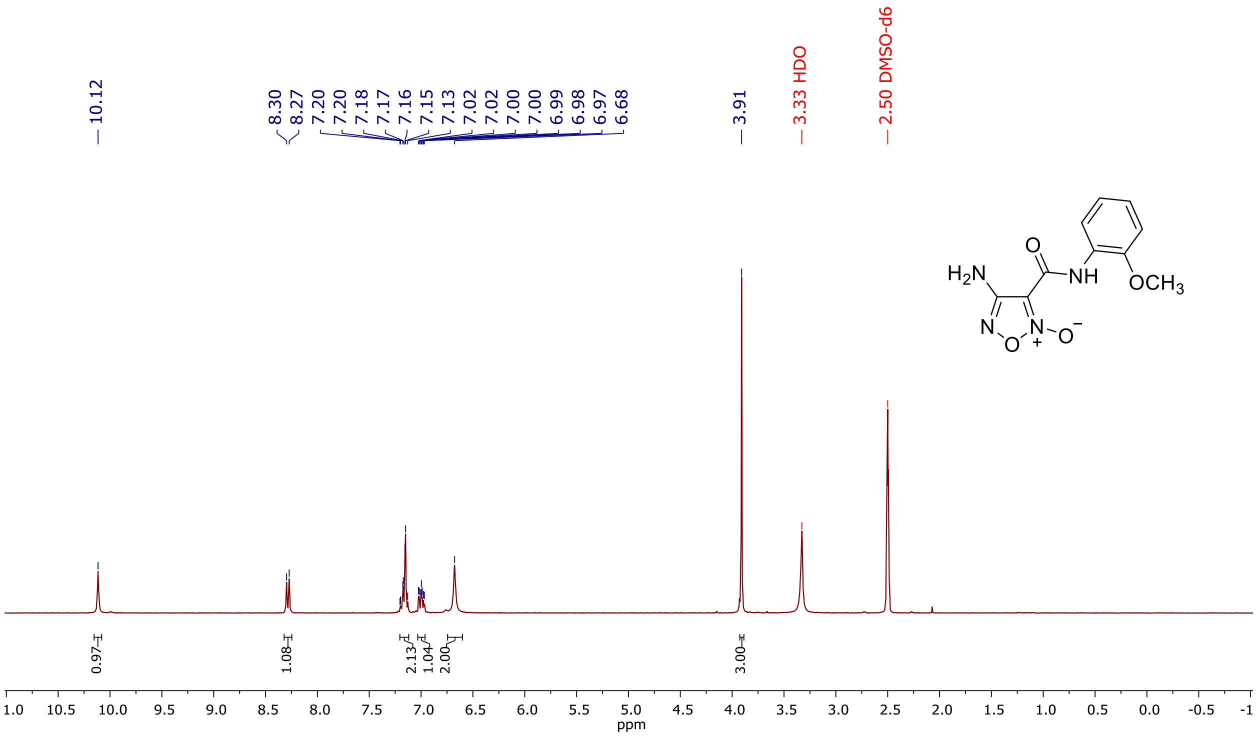

^{13}C {^{1}H} NMR spectrum (76 MHz) of **2e** in DMSO-d_6

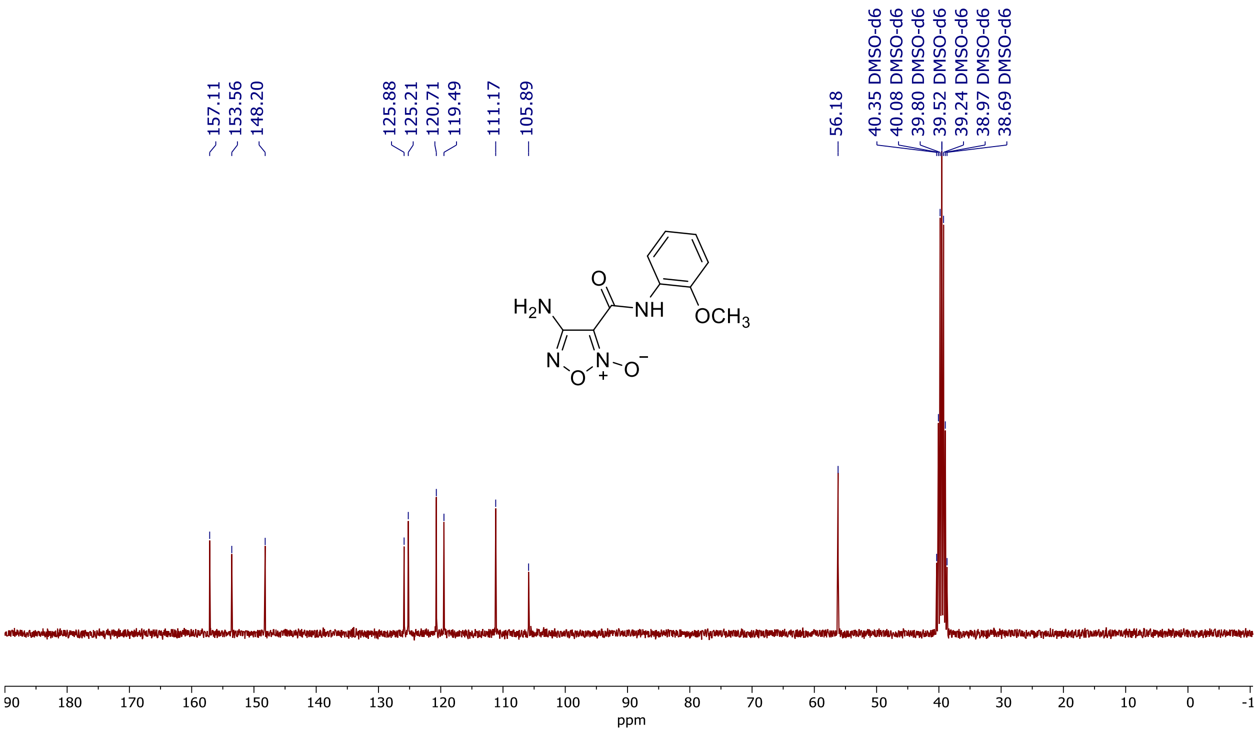

^{1}H NMR spectrum (300 MHz) of **2f** in DMSO-d_6

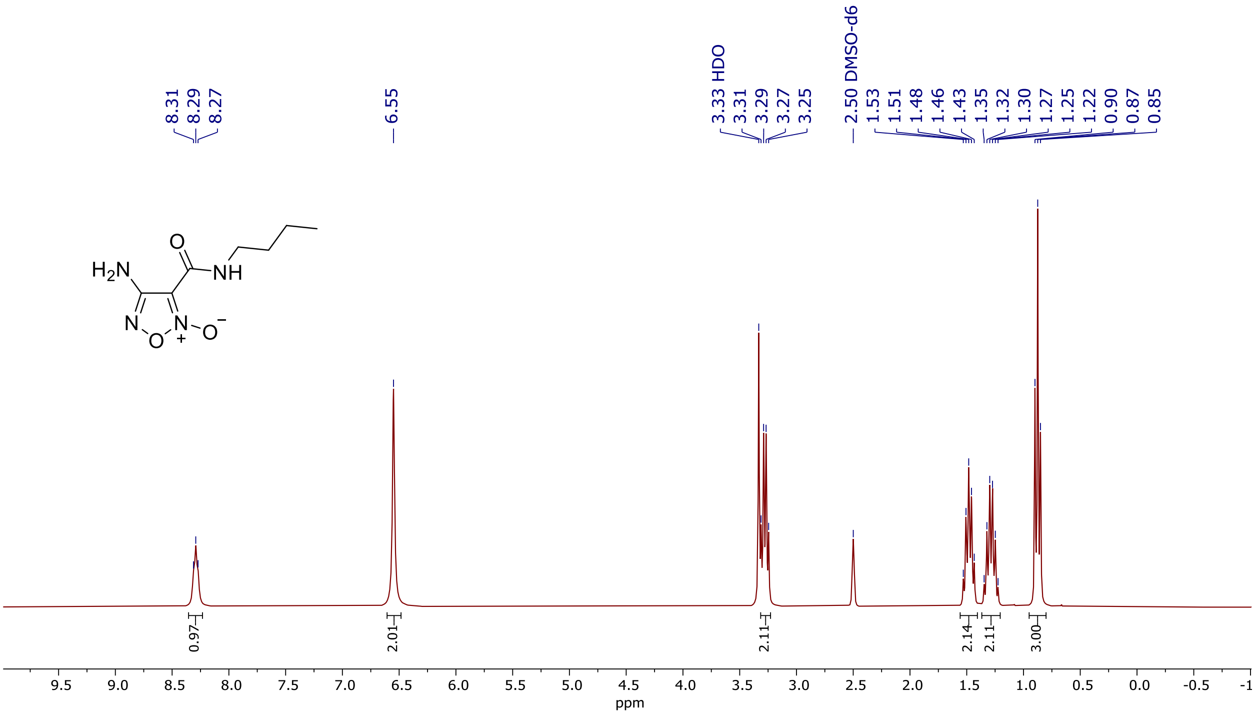

^{13}C {^{1}H} NMR spectrum (76 MHz) of **2f** in DMSO-d_6

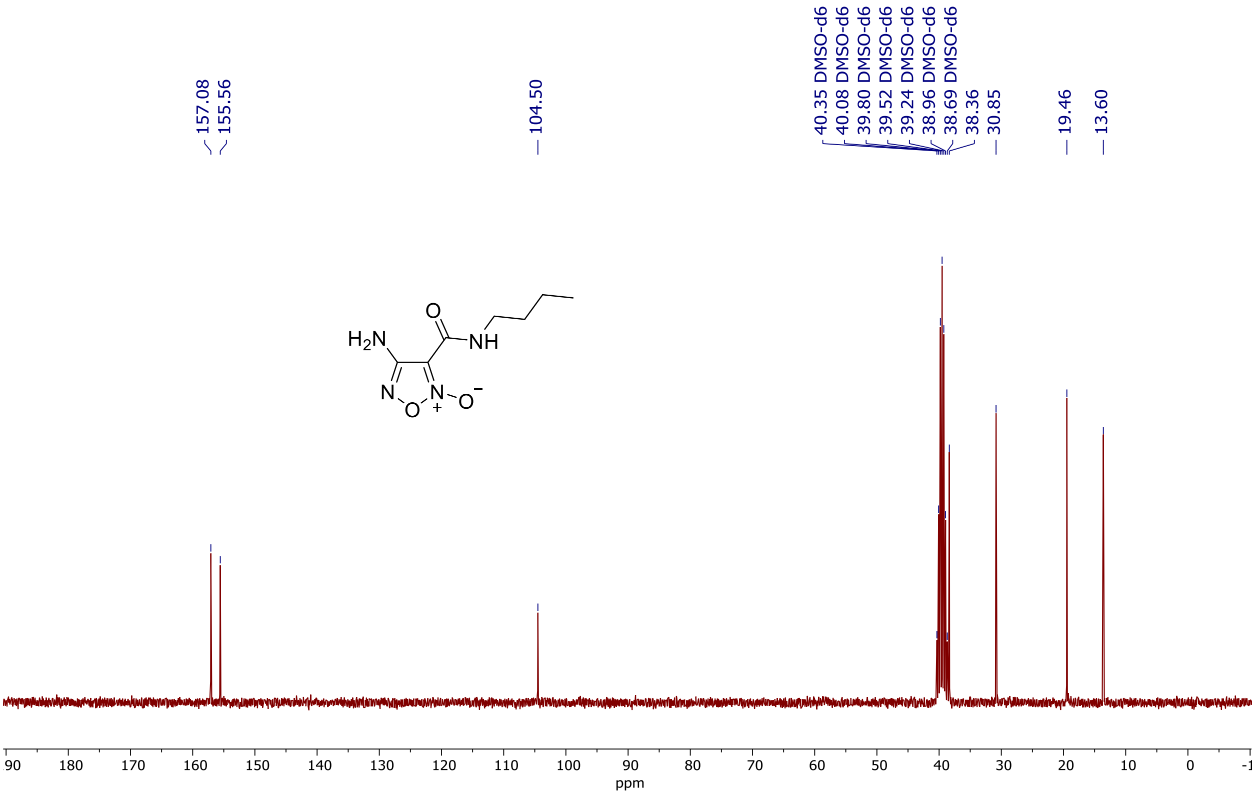

^{1}H NMR spectrum (300 MHz) of **2g** in DMSO-d_6

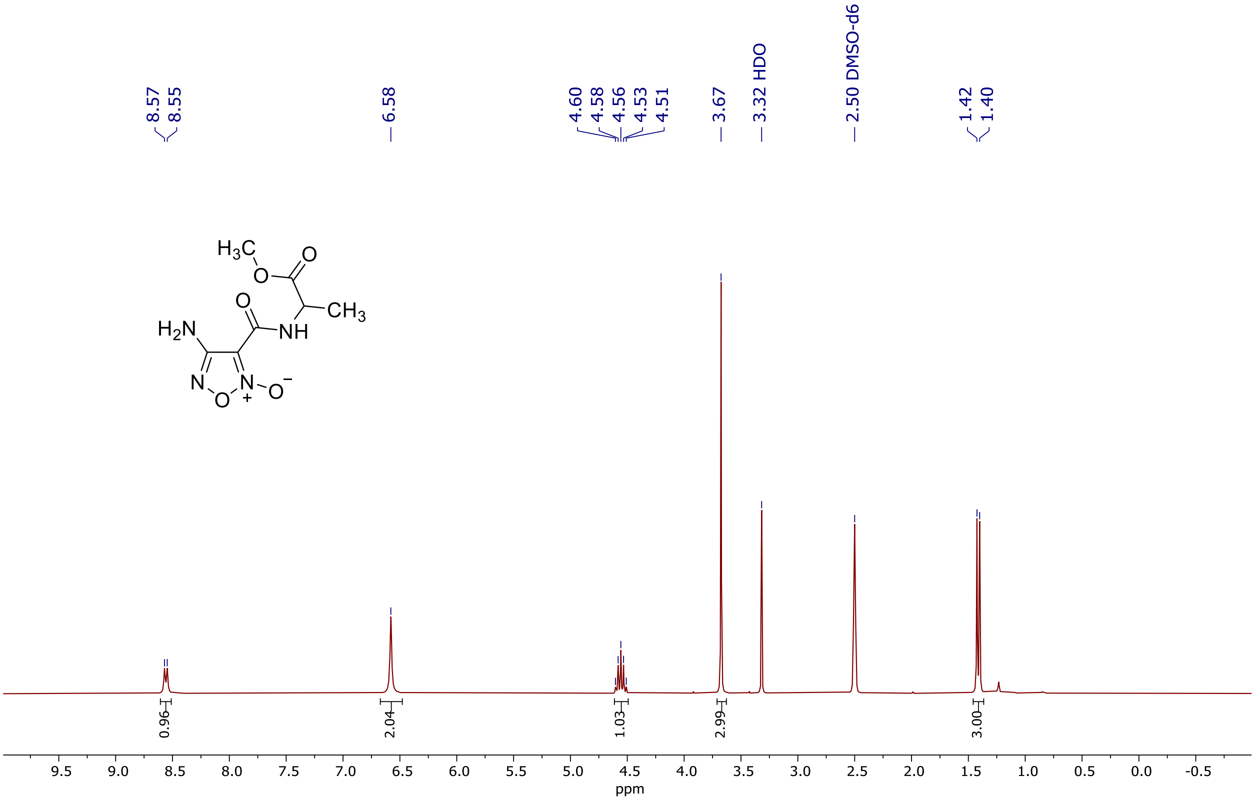

^{13}C {^{1}H} NMR spectrum (76 MHz) of **2g** in DMSO-d_6

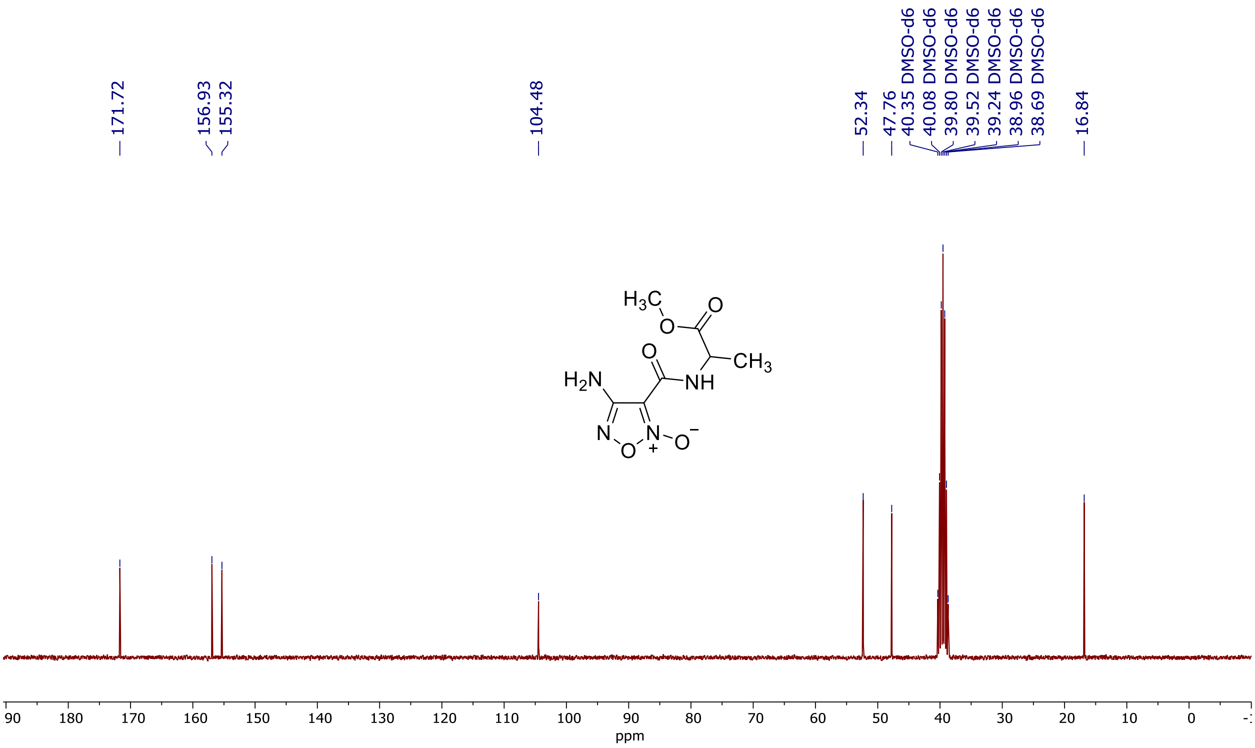

^{1}H NMR spectrum (300 MHz) of **2h** in DMSO-d_6

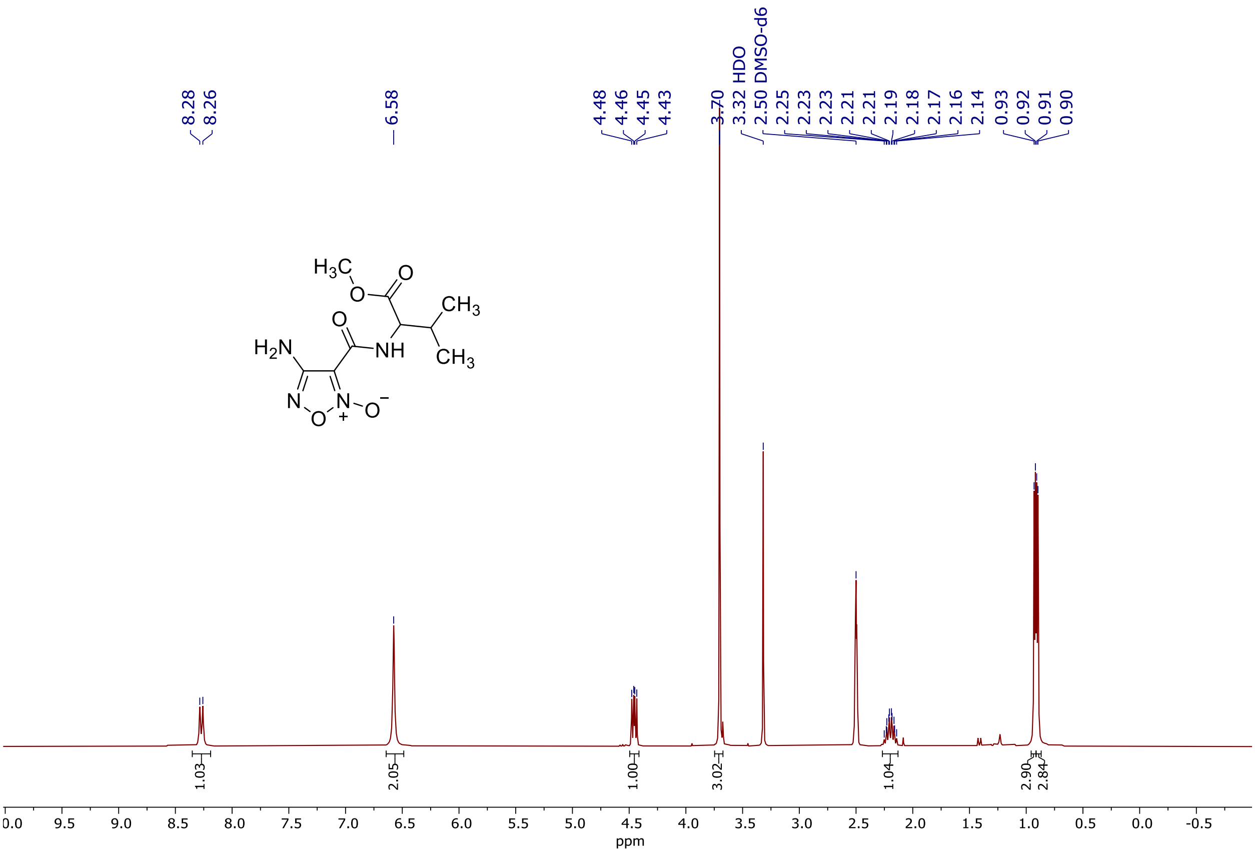

^{13}C {^{1}H} NMR spectrum (76 MHz) of **2h** in DMSO-d_6

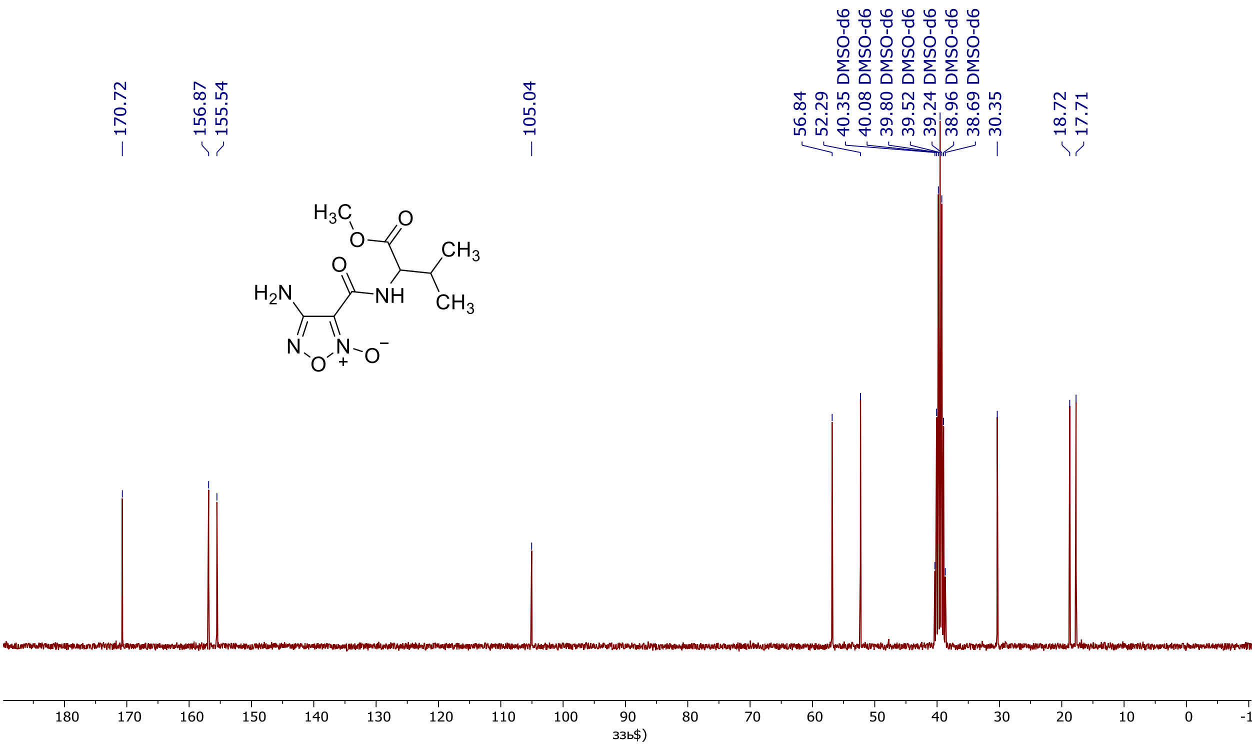

6.2 Copies of ^{1}H and ^{13}C NMR spectra for amides 5

^{1}H NMR spectrum (300 MHz) of **5a** in DMSO-d_6

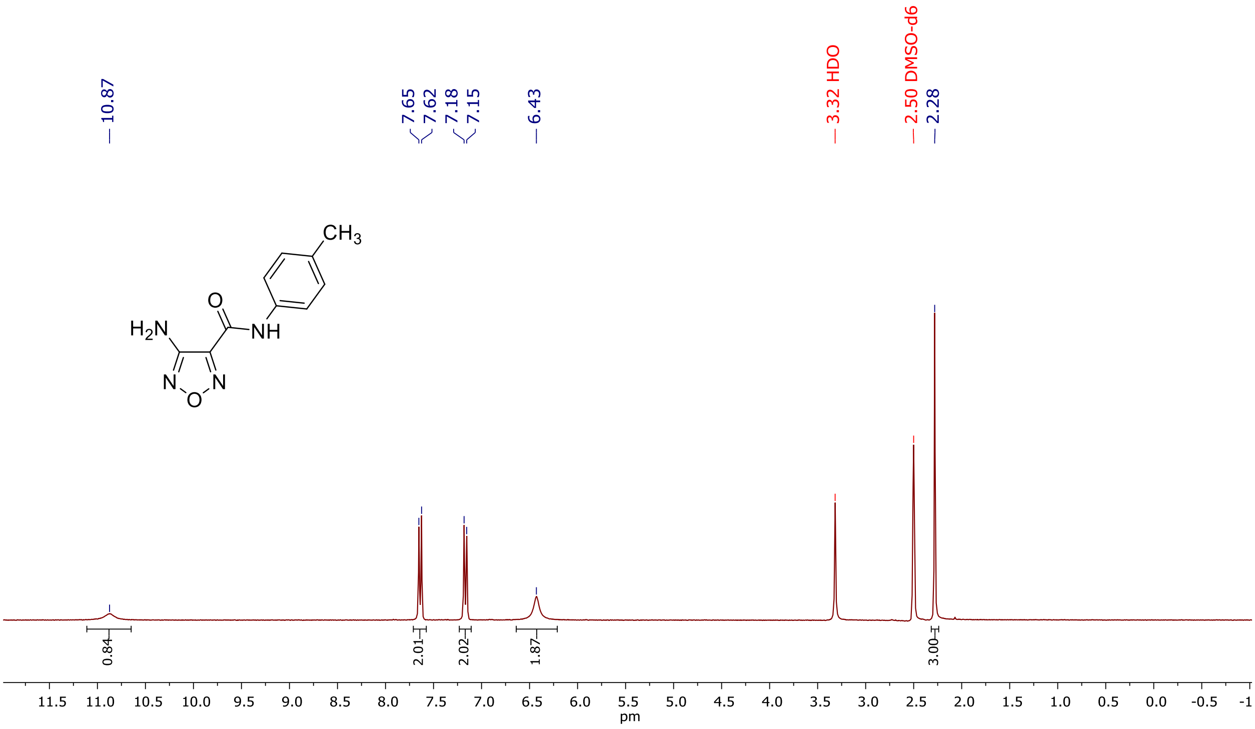

^{13}C {^{1}H} NMR spectrum (76 MHz) of **5a** in DMSO-d_6

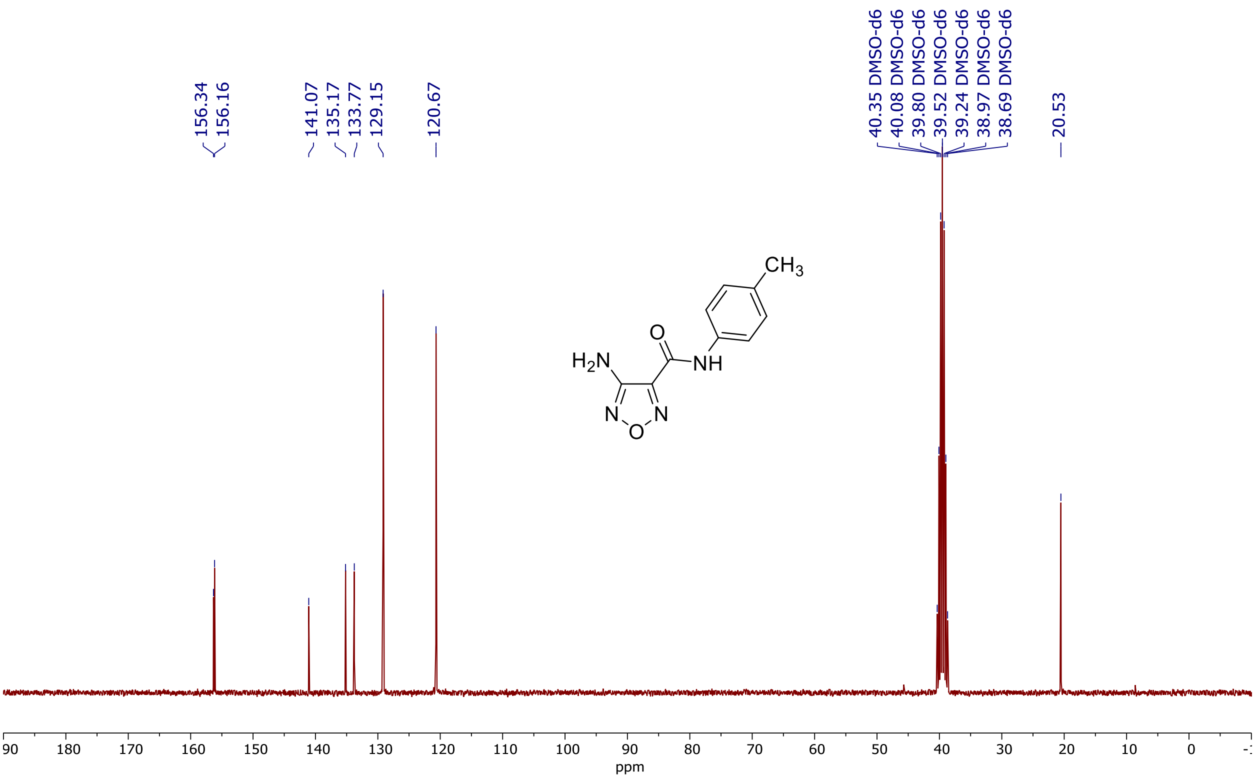

^{1}H NMR spectrum (300 MHz) of **5b** in DMSO-d_6

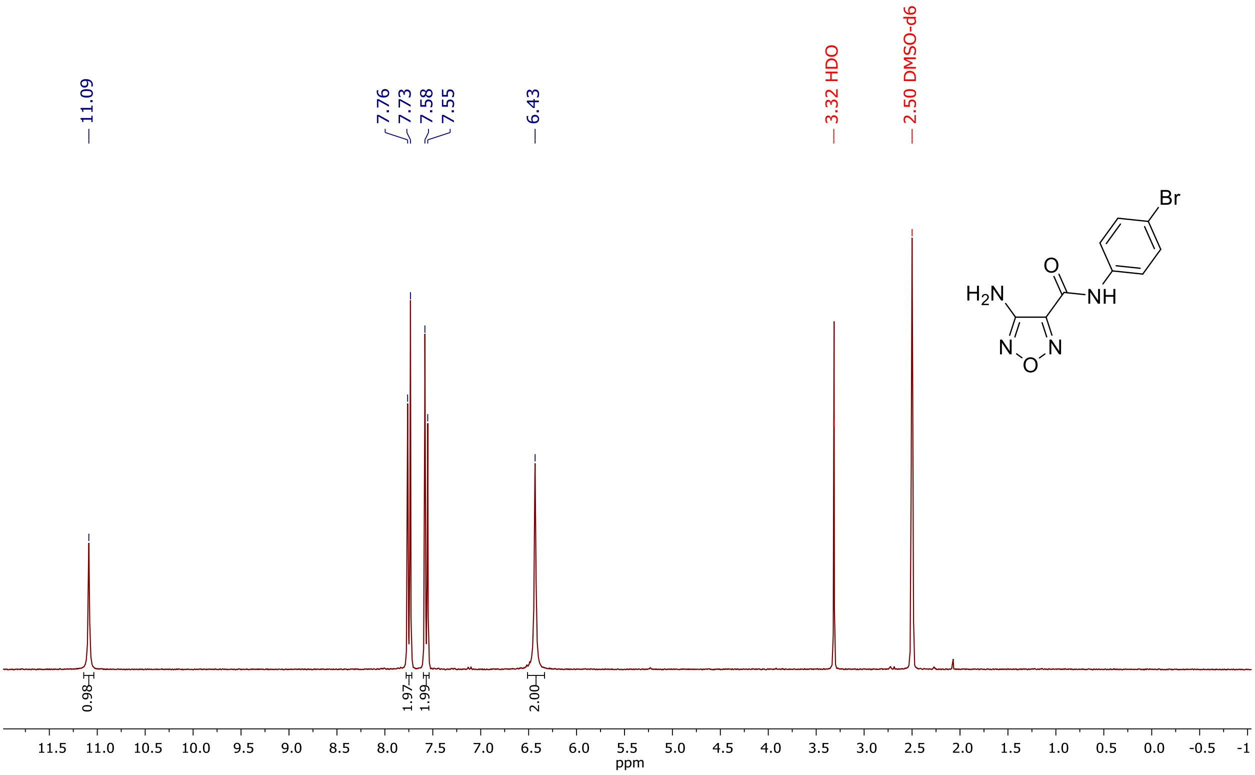

^{13}C {^{1}H} NMR spectrum (76 MHz) of **5b** in DMSO-d_6

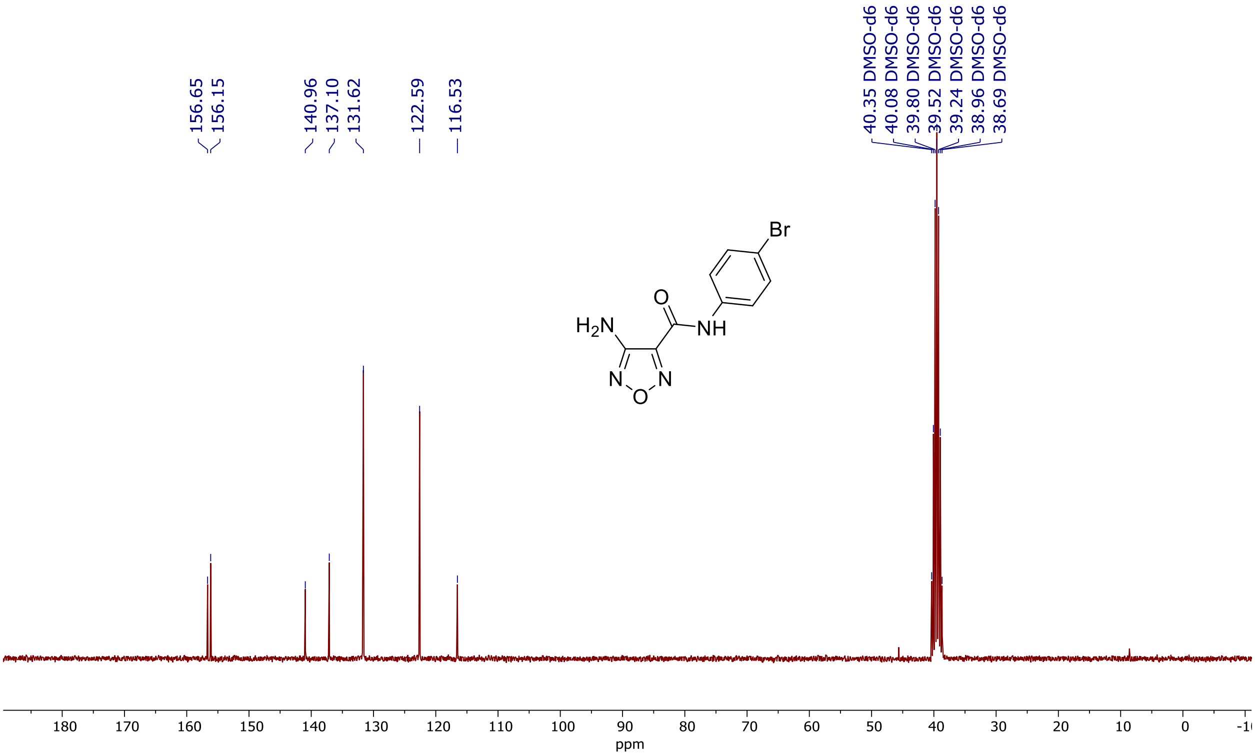

^{1}H NMR spectrum (300 MHz) of **5c** in DMSO-d_6

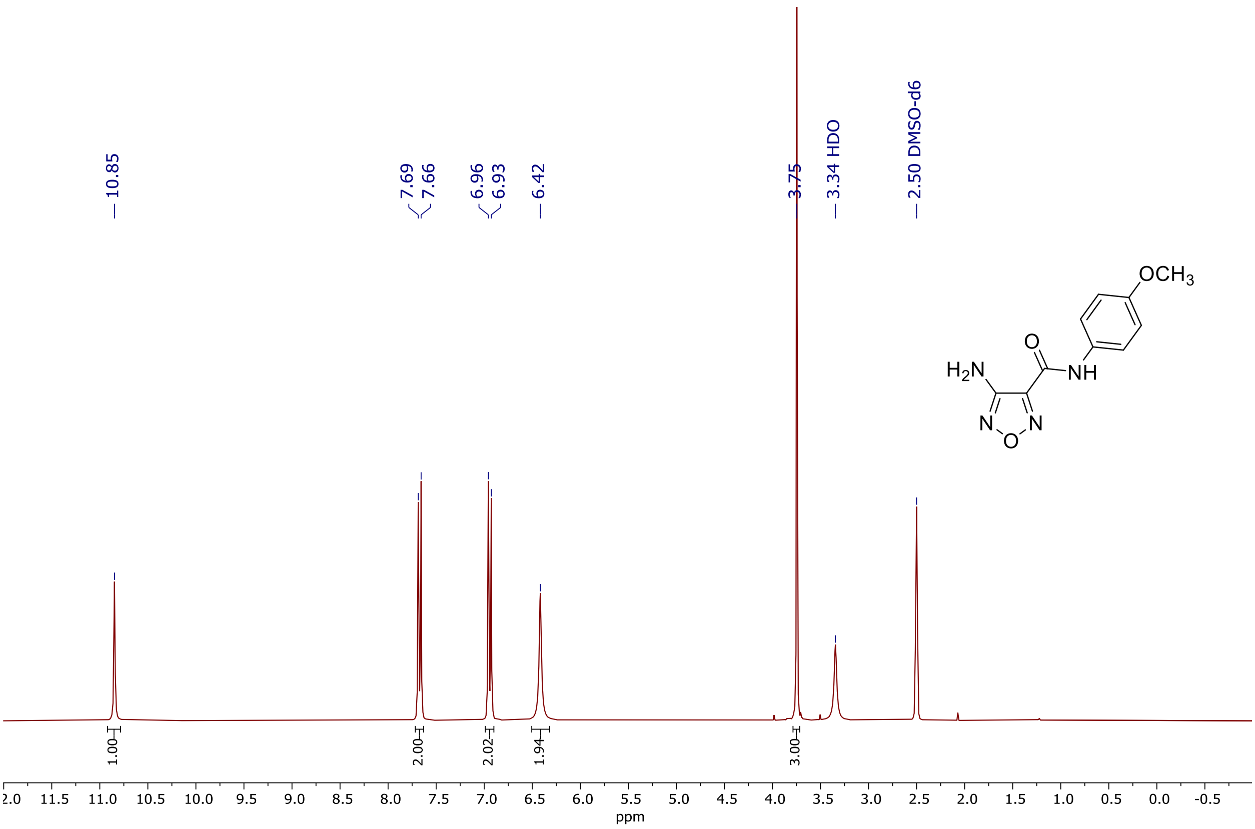

^{13}C {^{1}H} NMR spectrum (76 MHz) of **5c** in DMSO-d_6

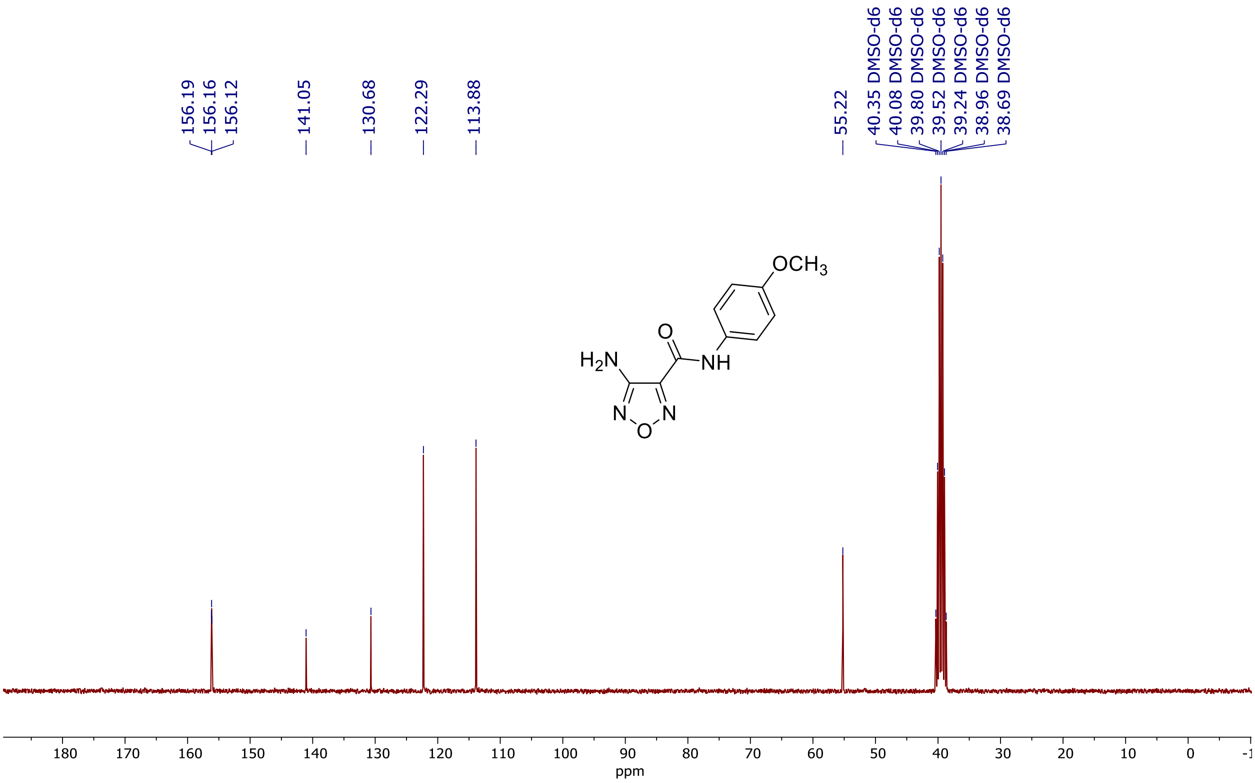

^{1}H NMR spectrum (300 MHz) of **5d** in DMSO-d_6

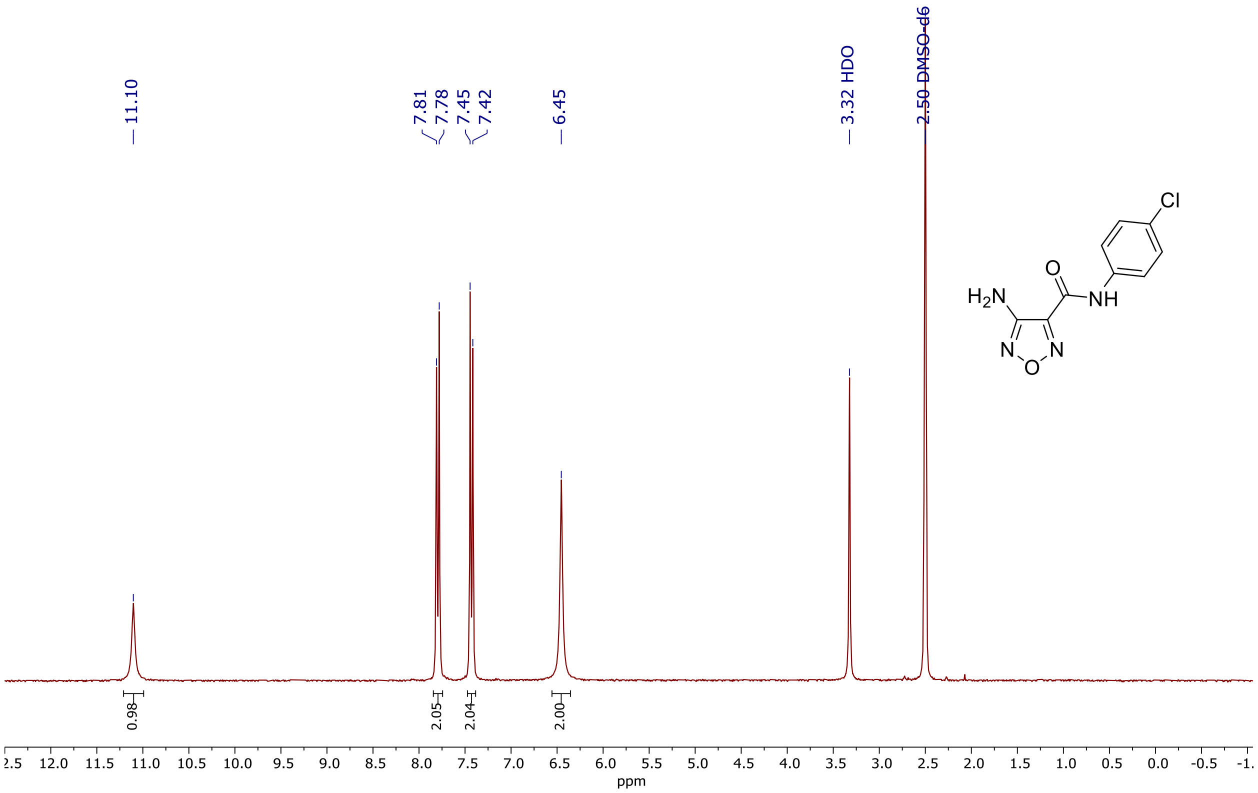

^{13}C {^{1}H} NMR spectrum (76 MHz) of **5d** in DMSO-d_6

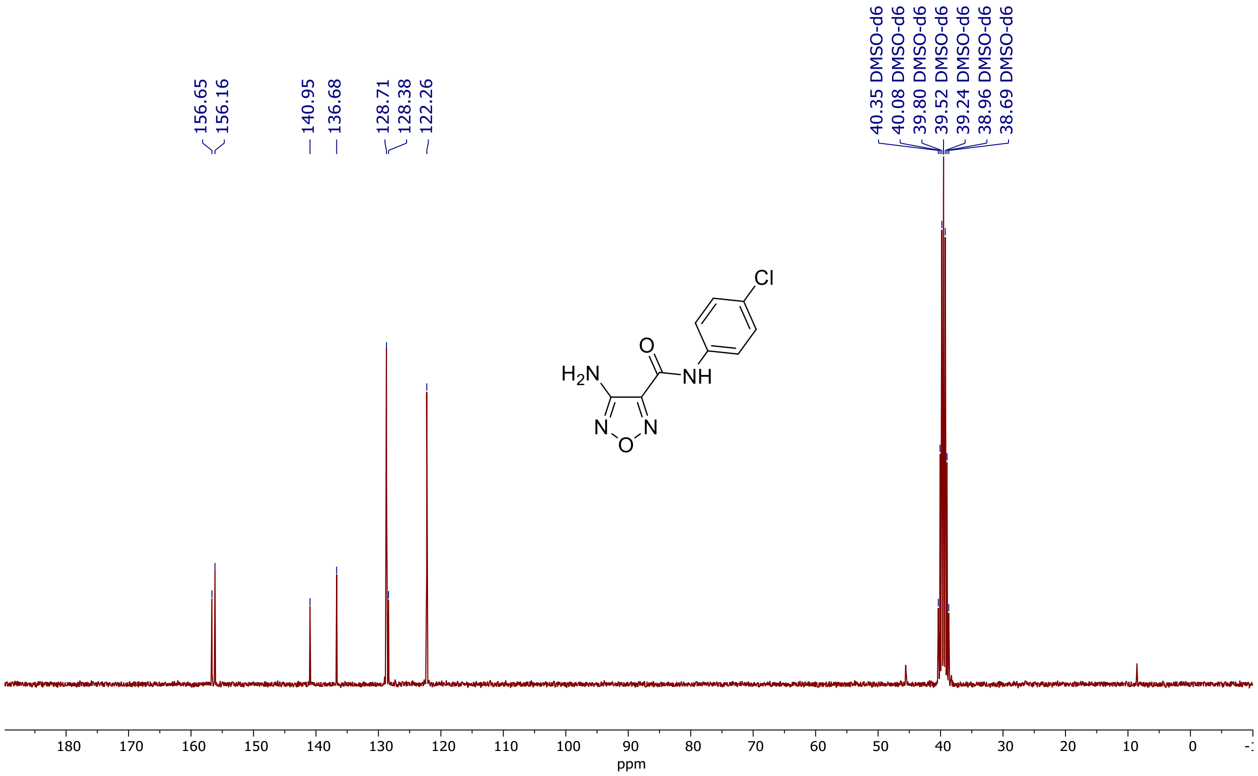

^{1}H NMR spectrum (300 MHz) of **5e** in DMSO-d_6

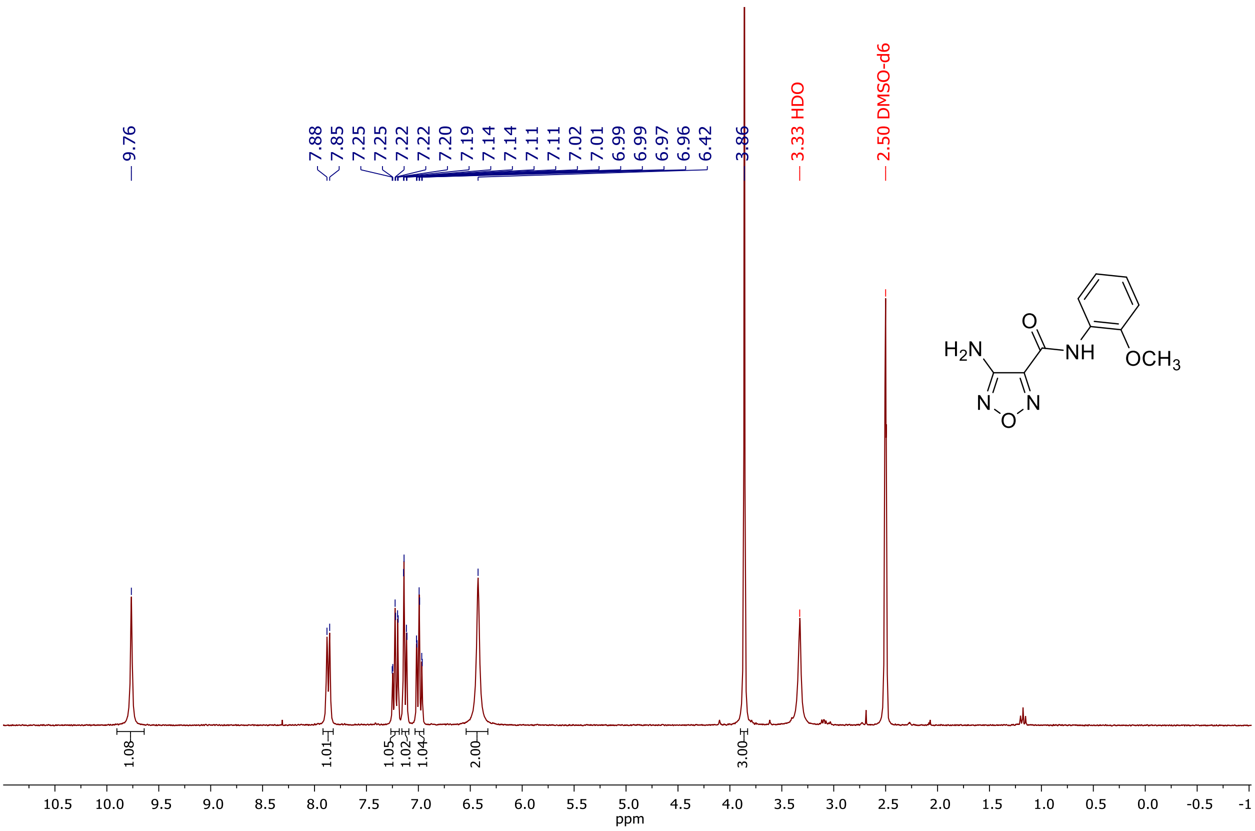

^{13}C {^{1}H} NMR spectrum (76 MHz) of **5e** in DMSO-d_6

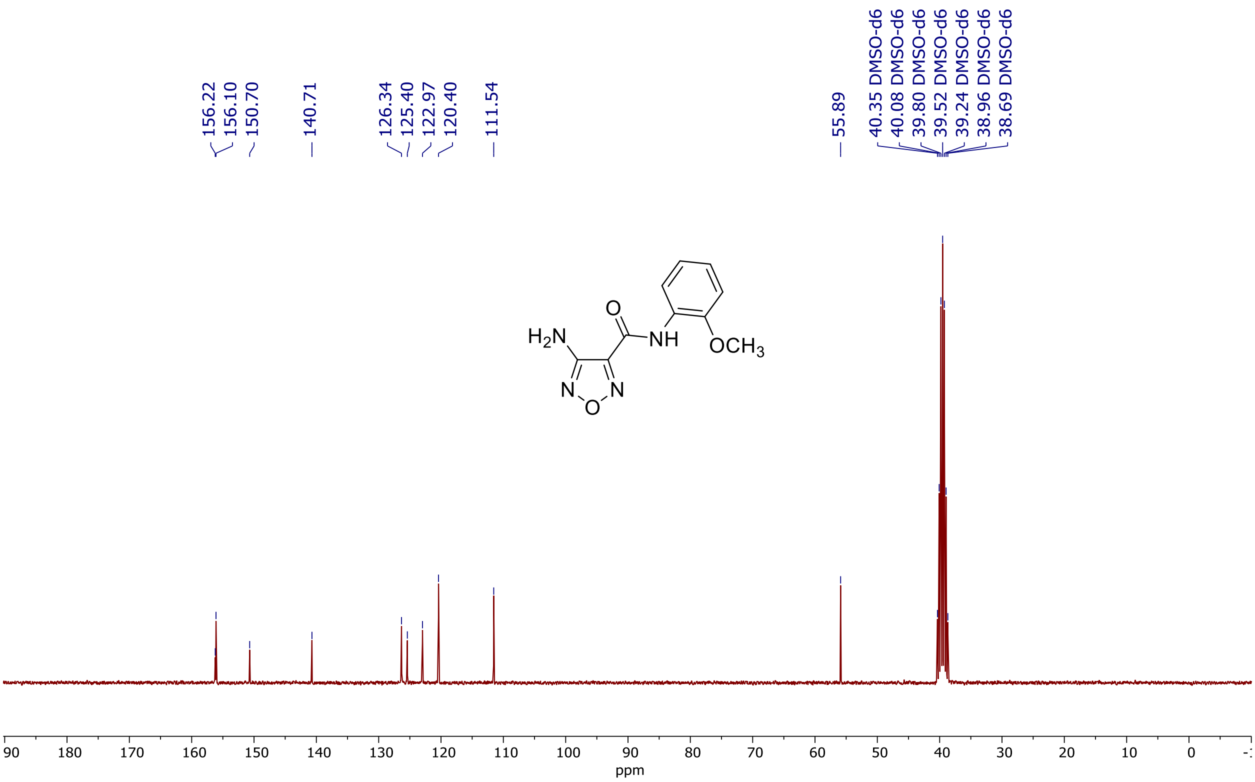

^{1}H NMR spectrum (300 MHz) of **5f** in DMSO-d_6

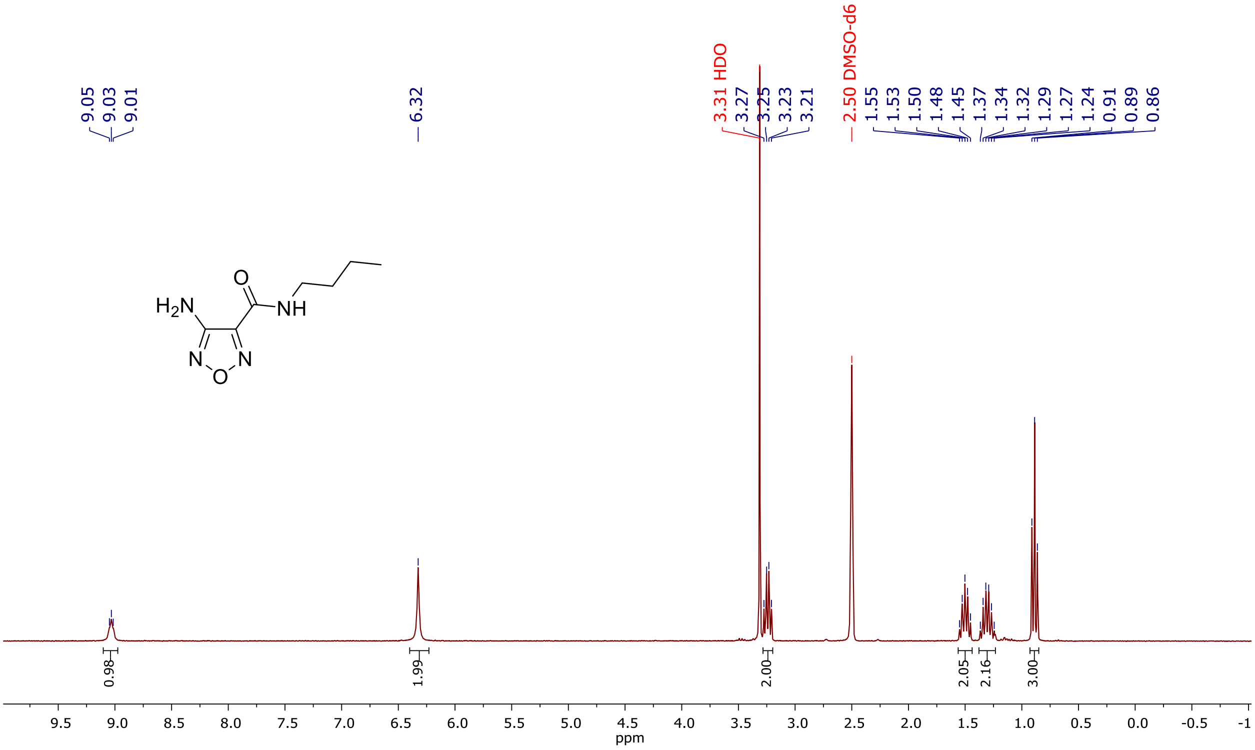

^{13}C {^{1}H} NMR spectrum (76 MHz) of **5f** in DMSO-d_6

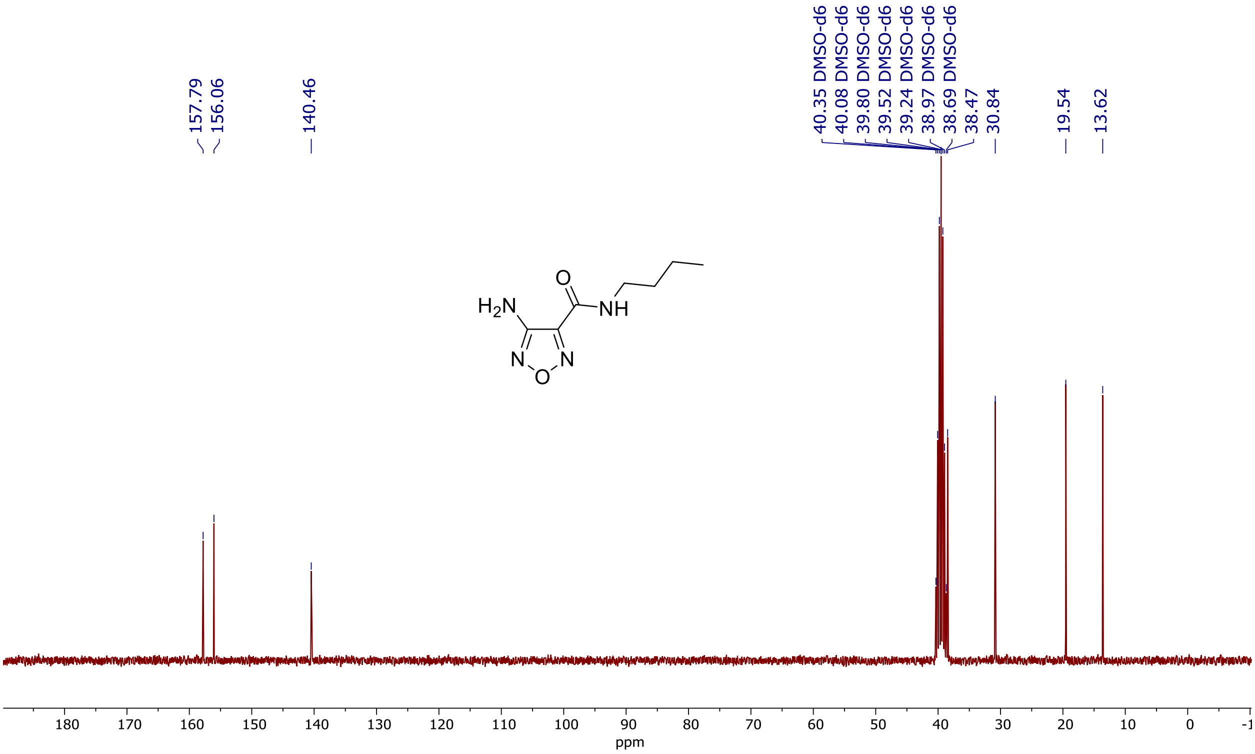

^{1}H NMR spectrum (300 MHz) of **5g** in DMSO-d_6

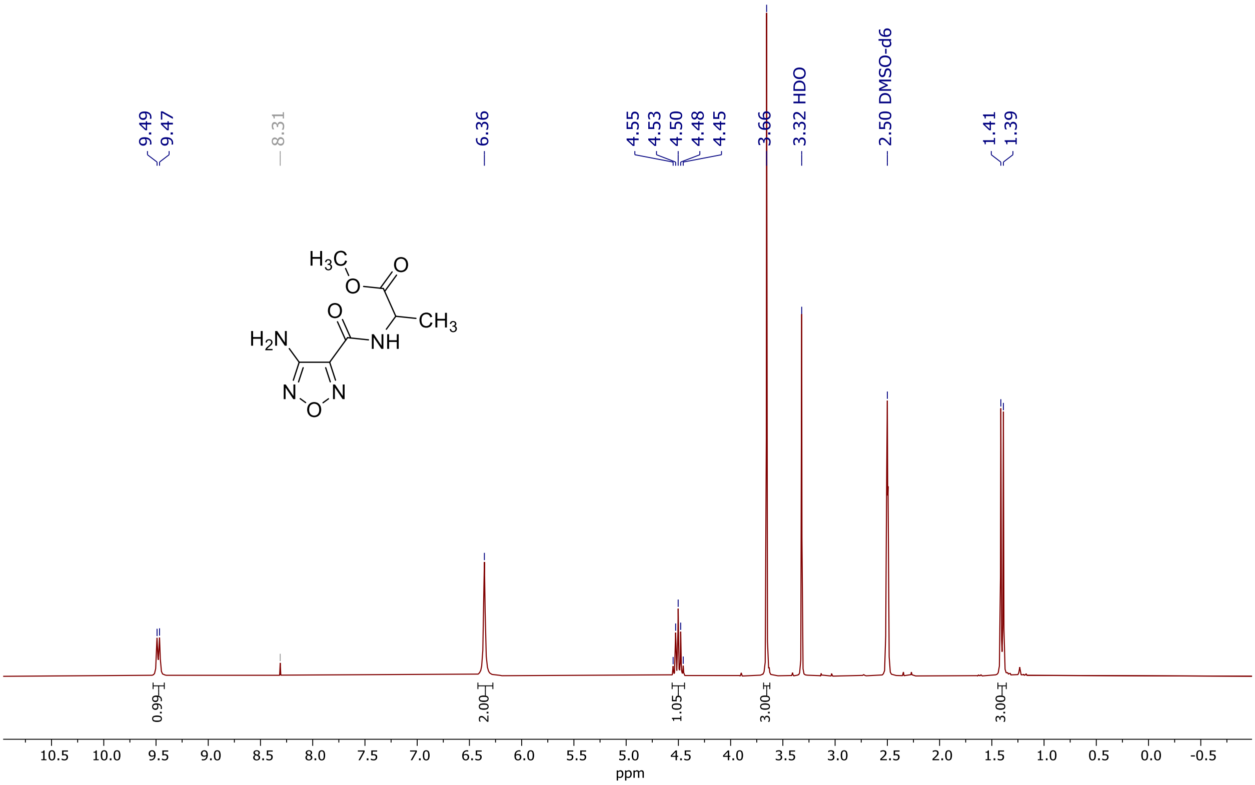

$^{13}C\ \{^{1}H\}$ NMR spectrum (76 MHz) of **5g** in DMSO-d_6

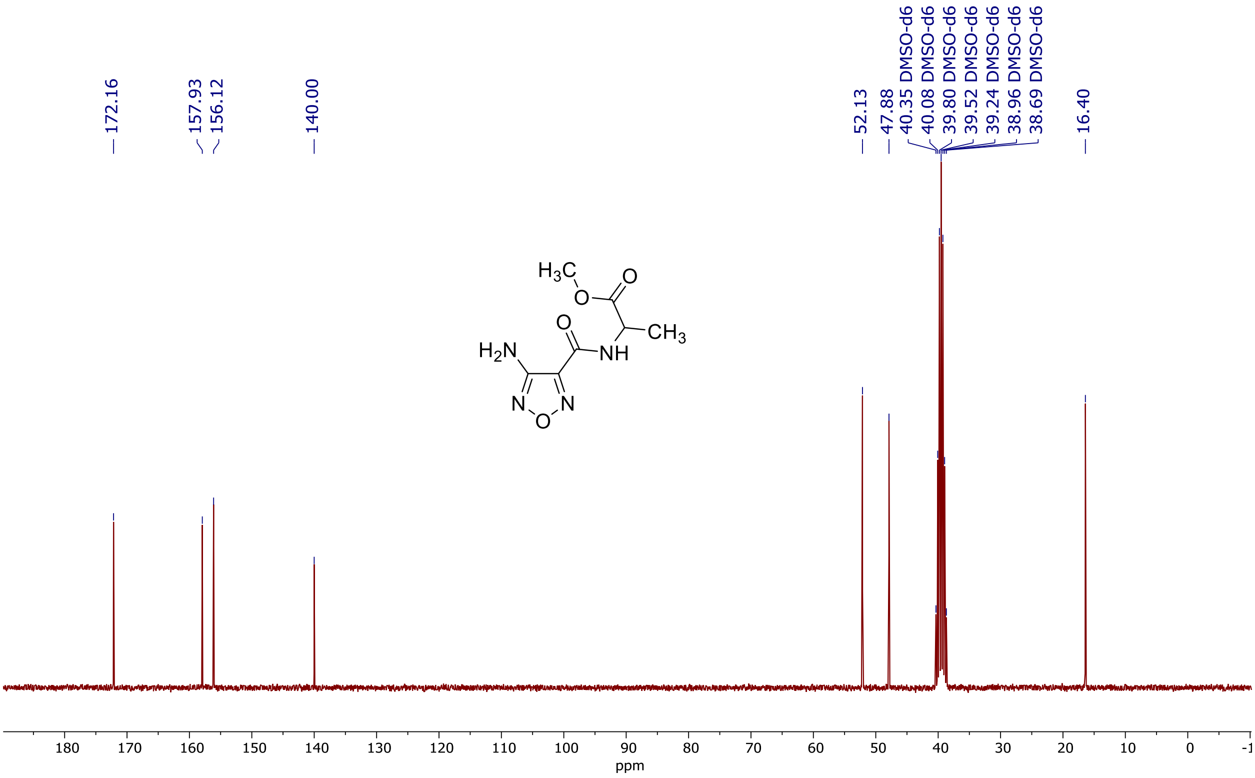

^{1}H NMR spectrum (300 MHz) of **5h** in DMSO-d_6

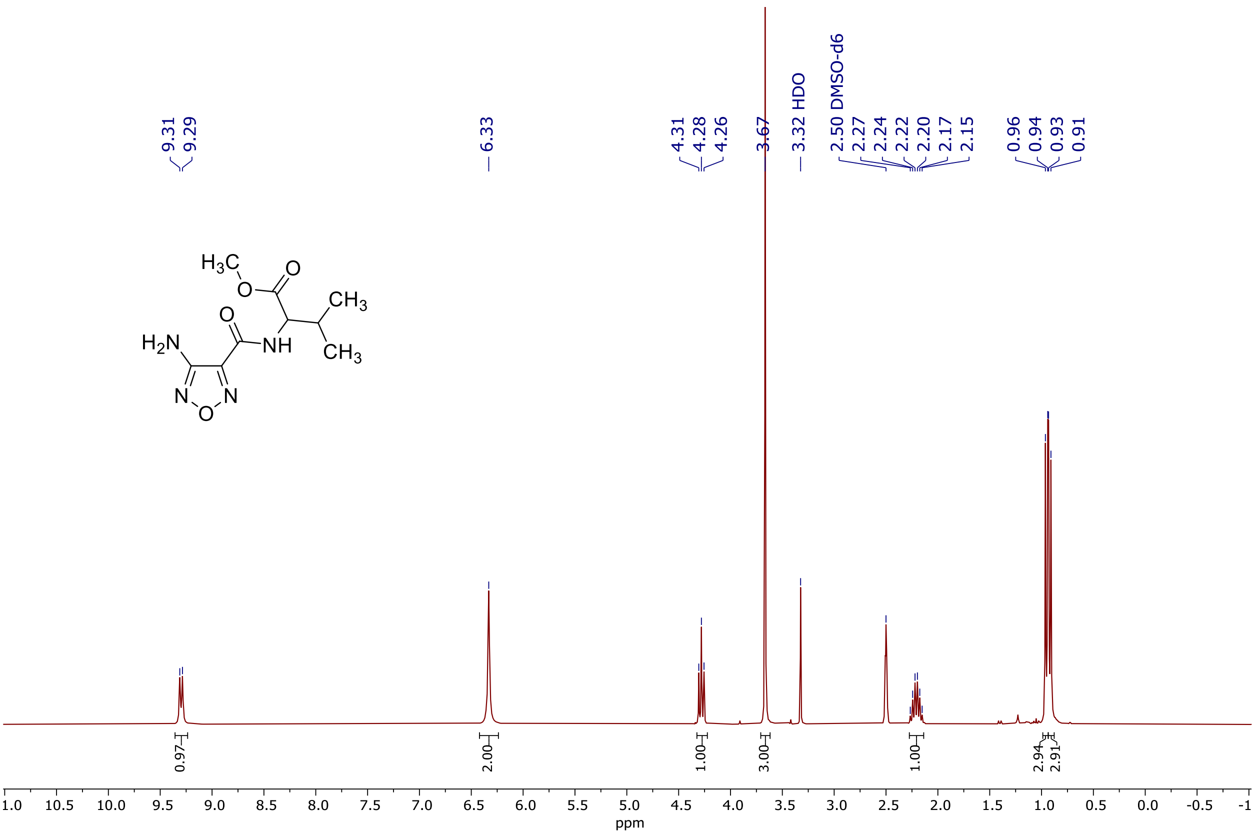

^{13}C {^{1}H} NMR spectrum (76 MHz) of **5h** in DMSO-d_6

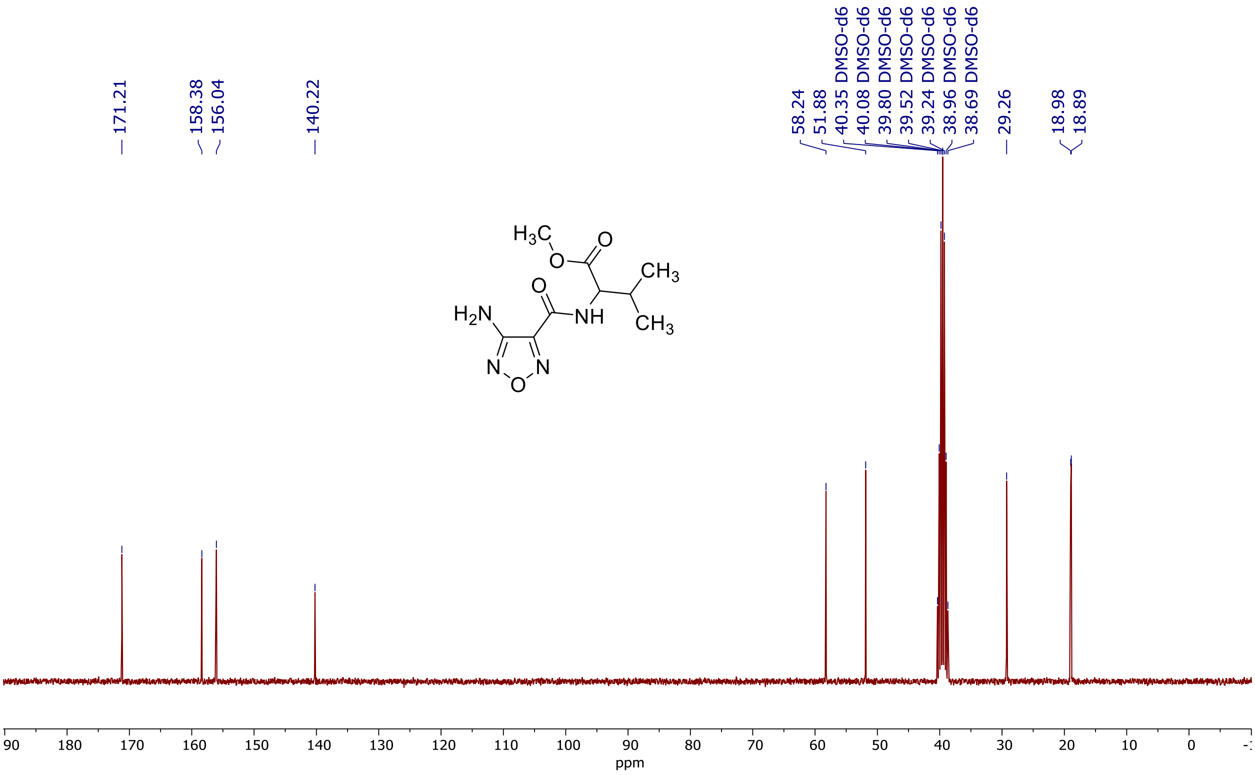

6.3 Copies of ^{1}H and ^{13}C NMR spectra for target products 1 and 7

^{1}H NMR spectrum (300 MHz) of **1a** in DMSO-d_6

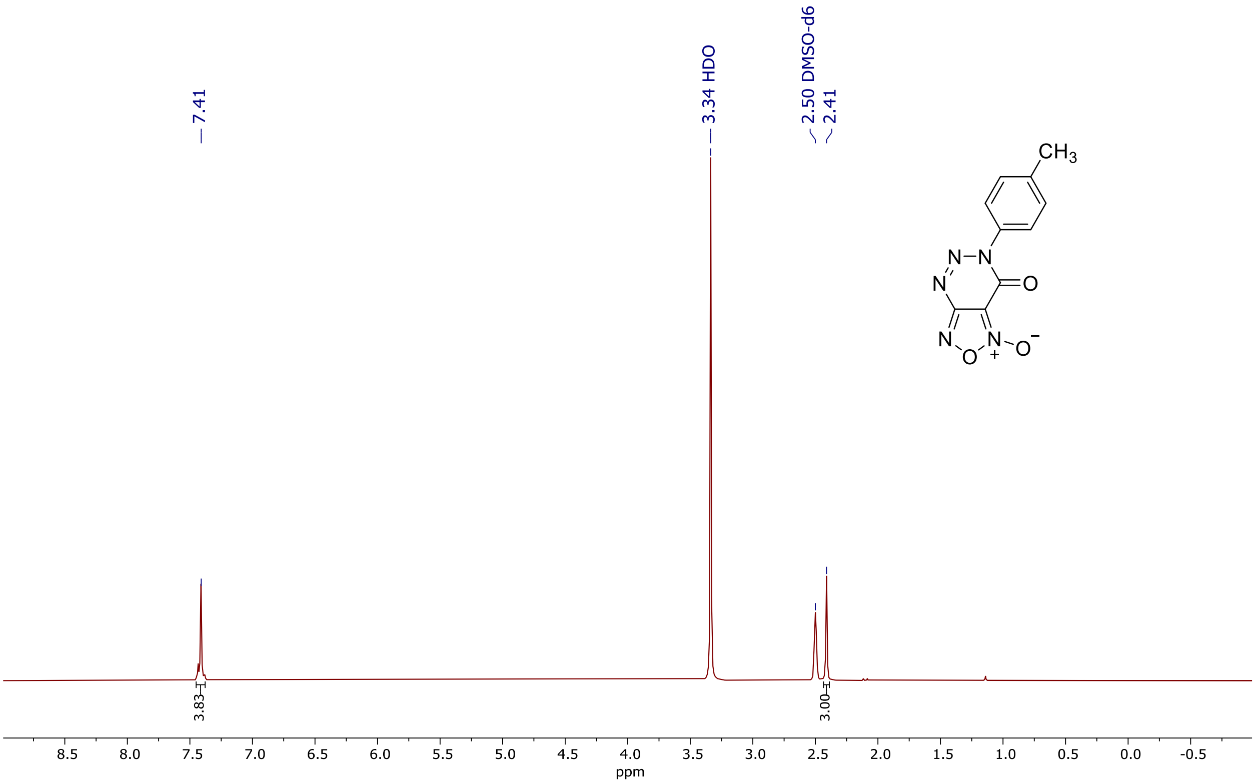

^{13}C {^{1}H} NMR spectrum (76 MHz) of **1a** in DMSO-d_6

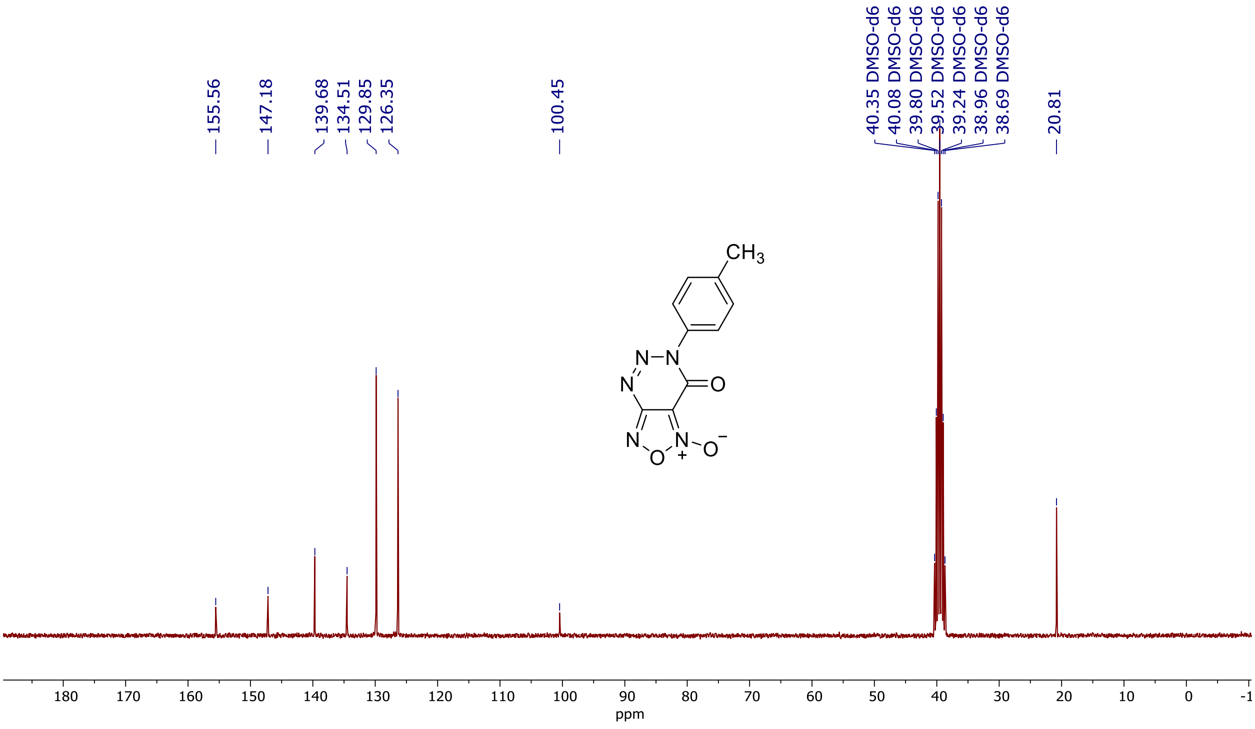

^{1}H NMR spectrum (300 MHz) of **1b** in DMSO-d_6

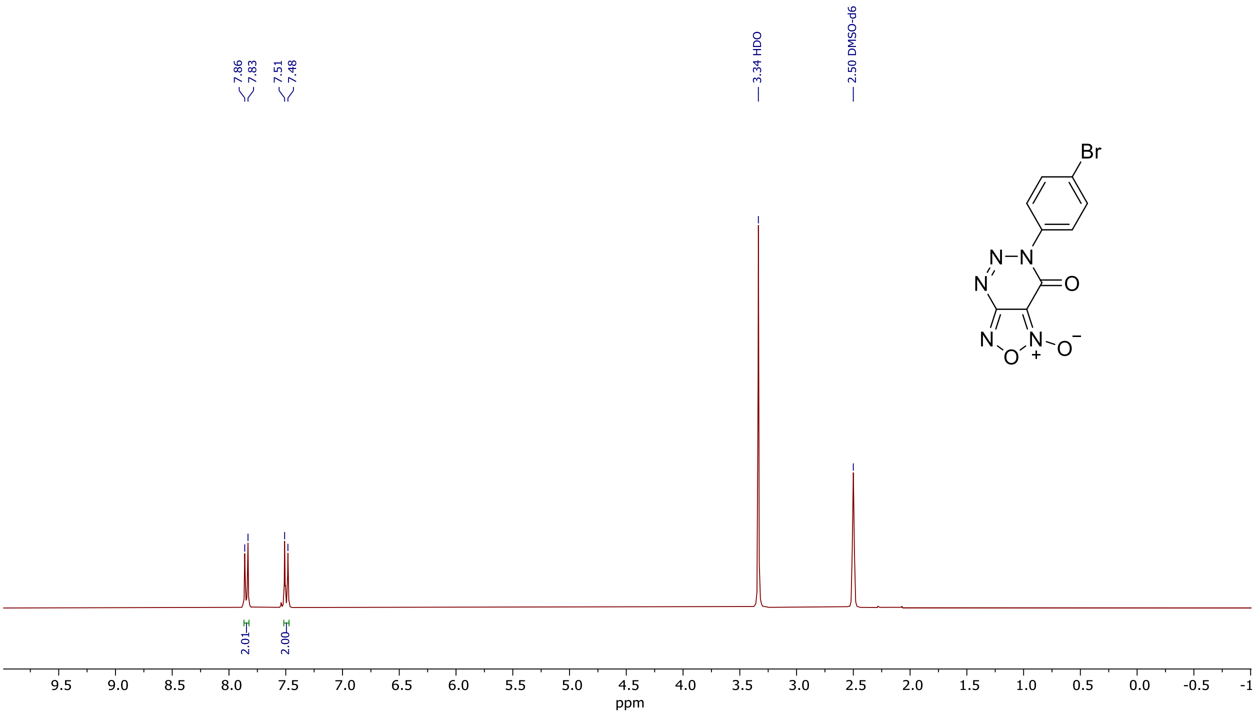

^{13}C {^{1}H} NMR spectrum (76 MHz) of **1b** in DMSO-d_6

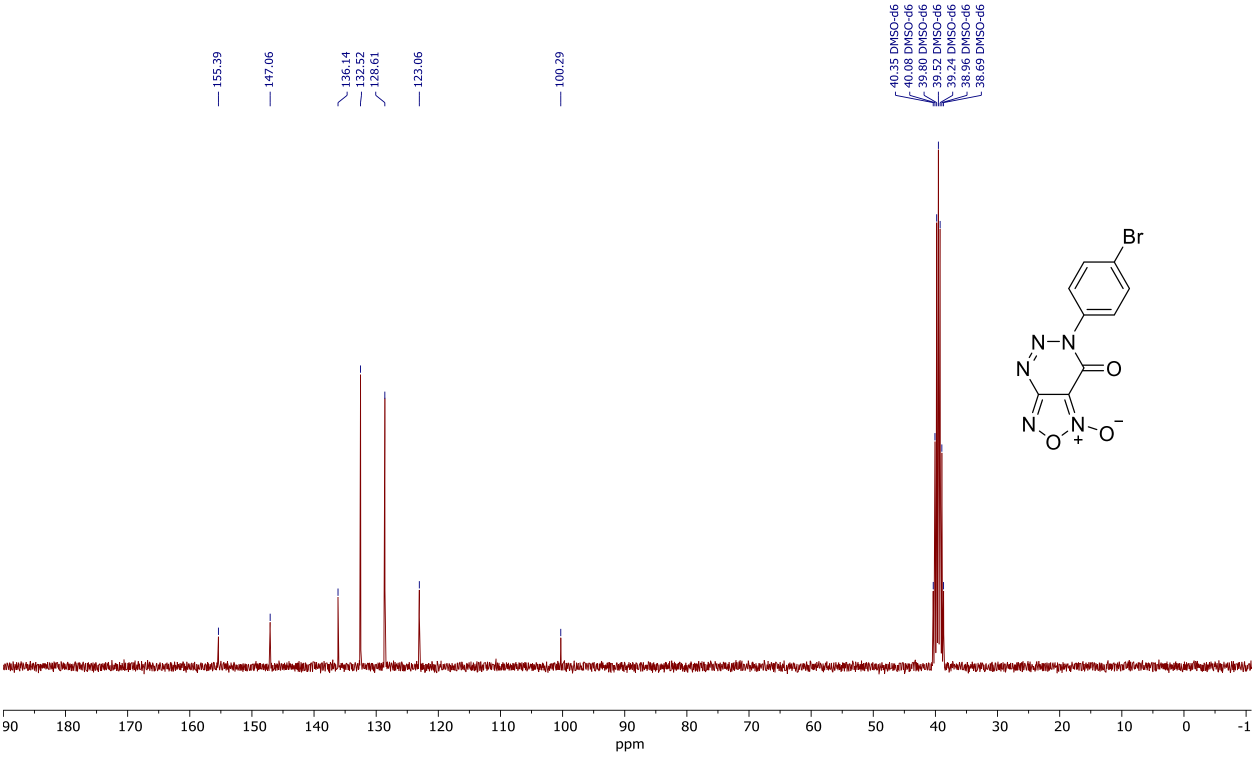

^{1}H NMR spectrum (300 MHz) of **1c** in DMSO-d_6

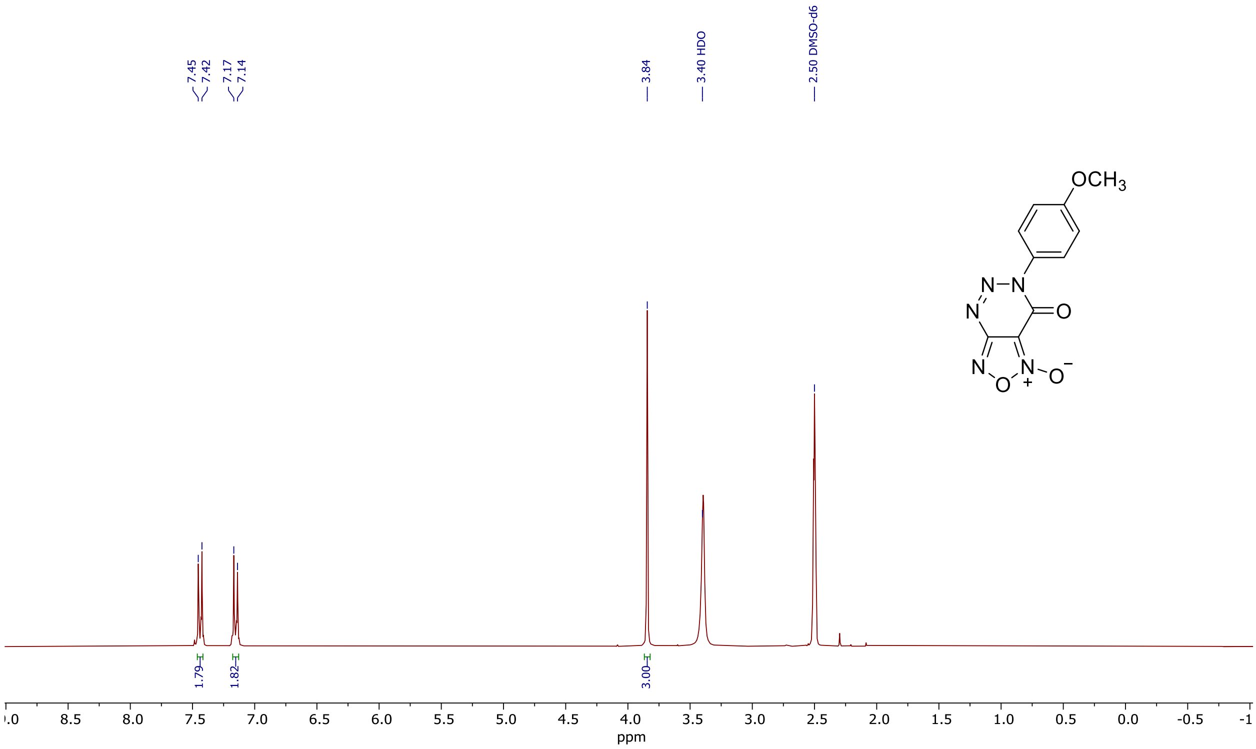

^{13}C {^{1}H} NMR spectrum (76 MHz) of **1c** in DMSO-d_6

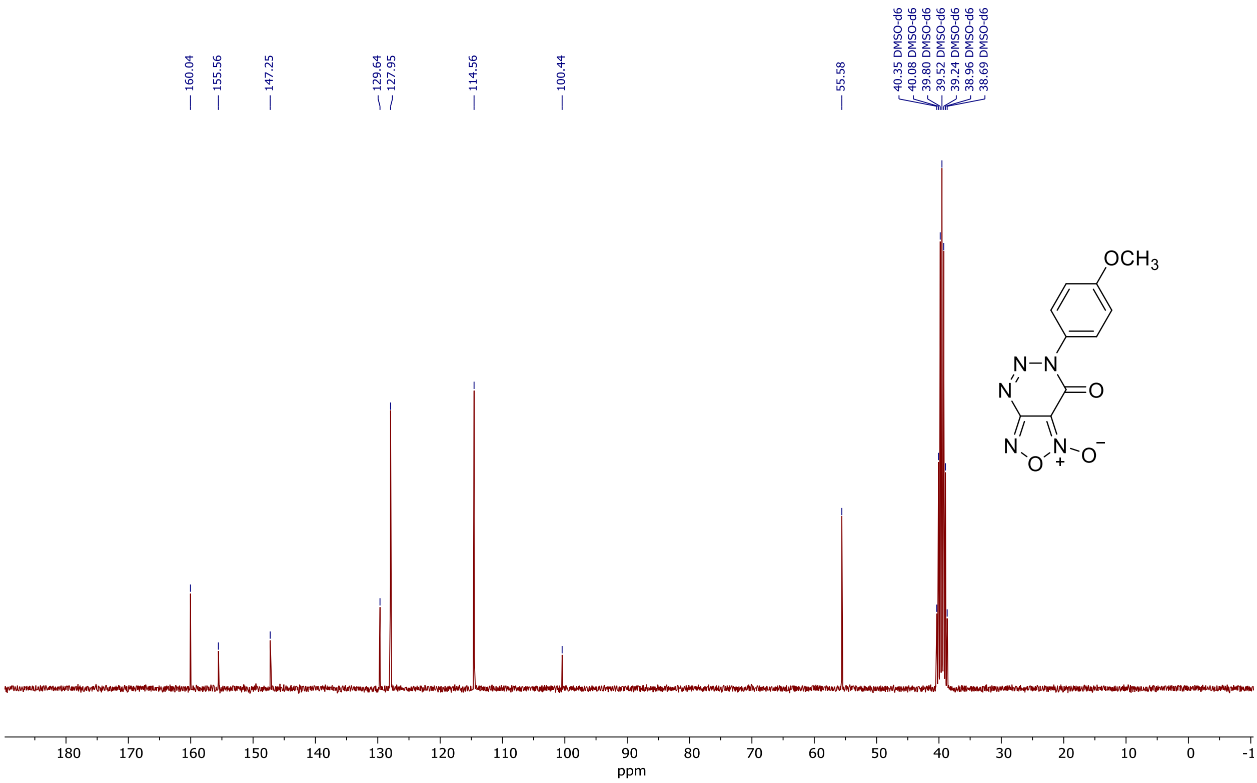

^{1}H NMR spectrum (300 MHz) of **1d** in DMSO-d_6

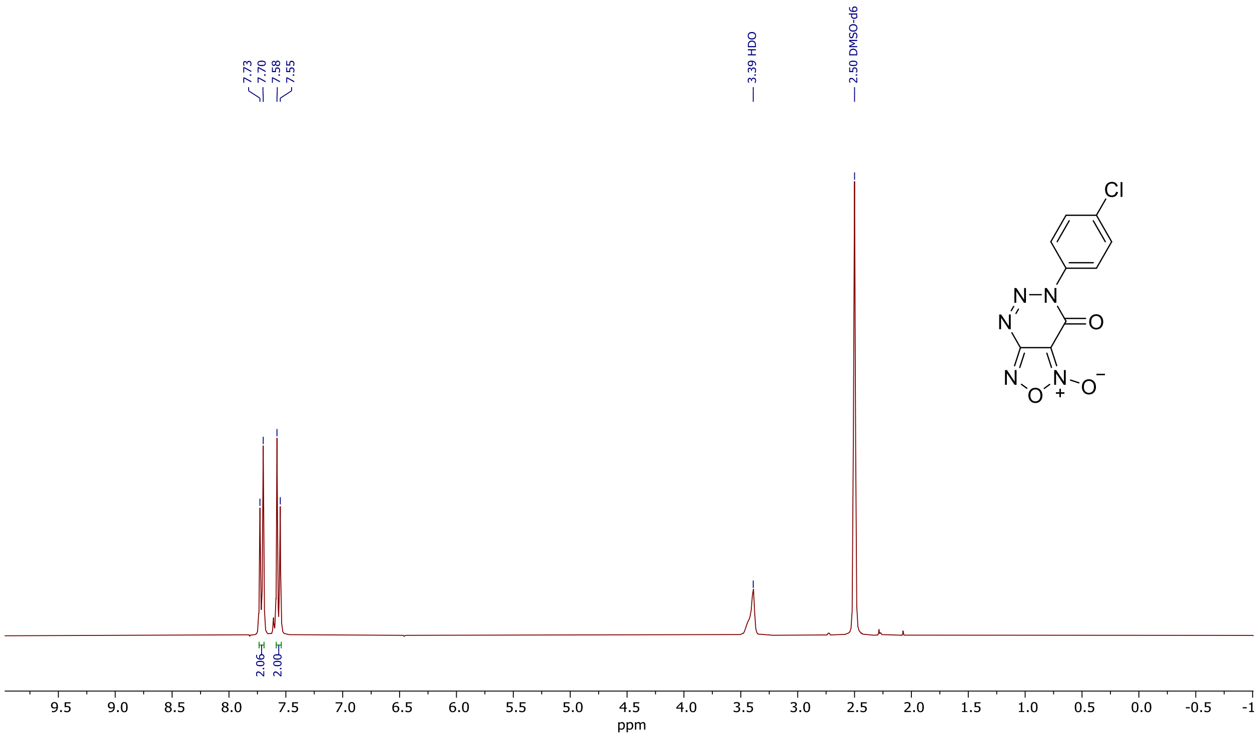

^{13}C {^{1}H} NMR spectrum (76 MHz) of **1d** in DMSO-d_6

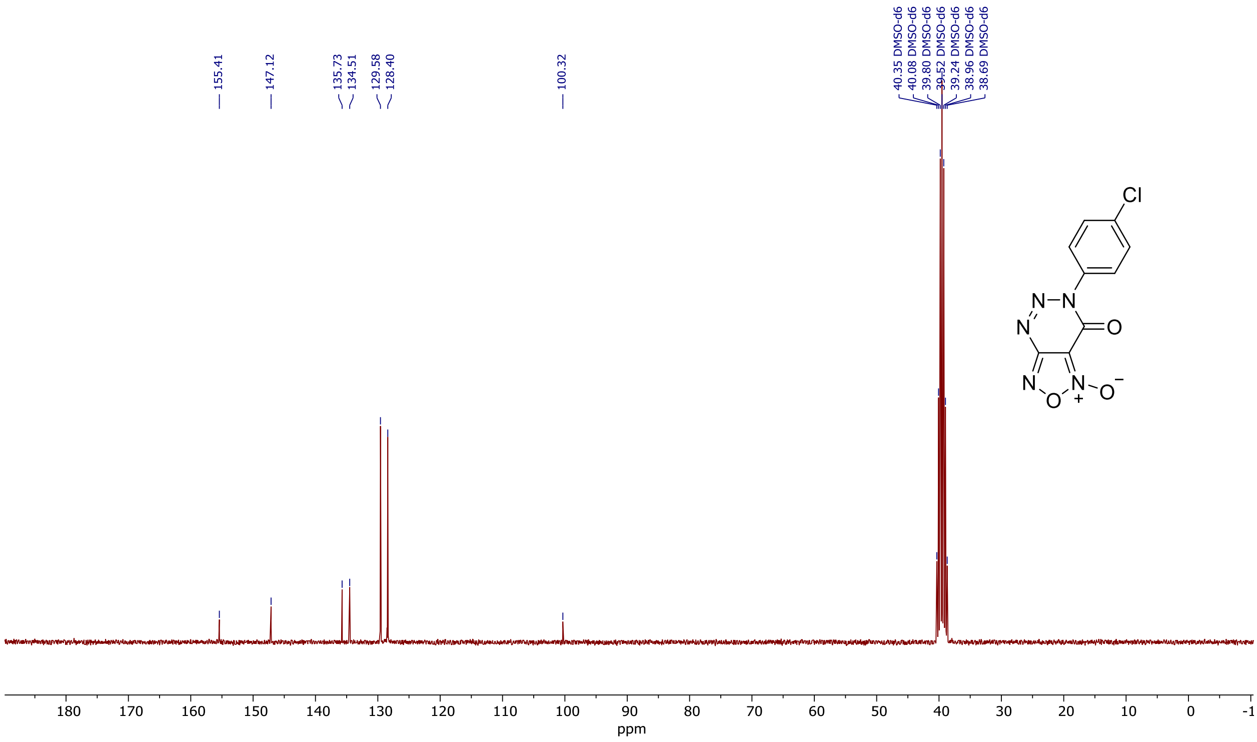

^{1}H NMR spectrum (300 MHz) of **1e** in DMSO-d_6

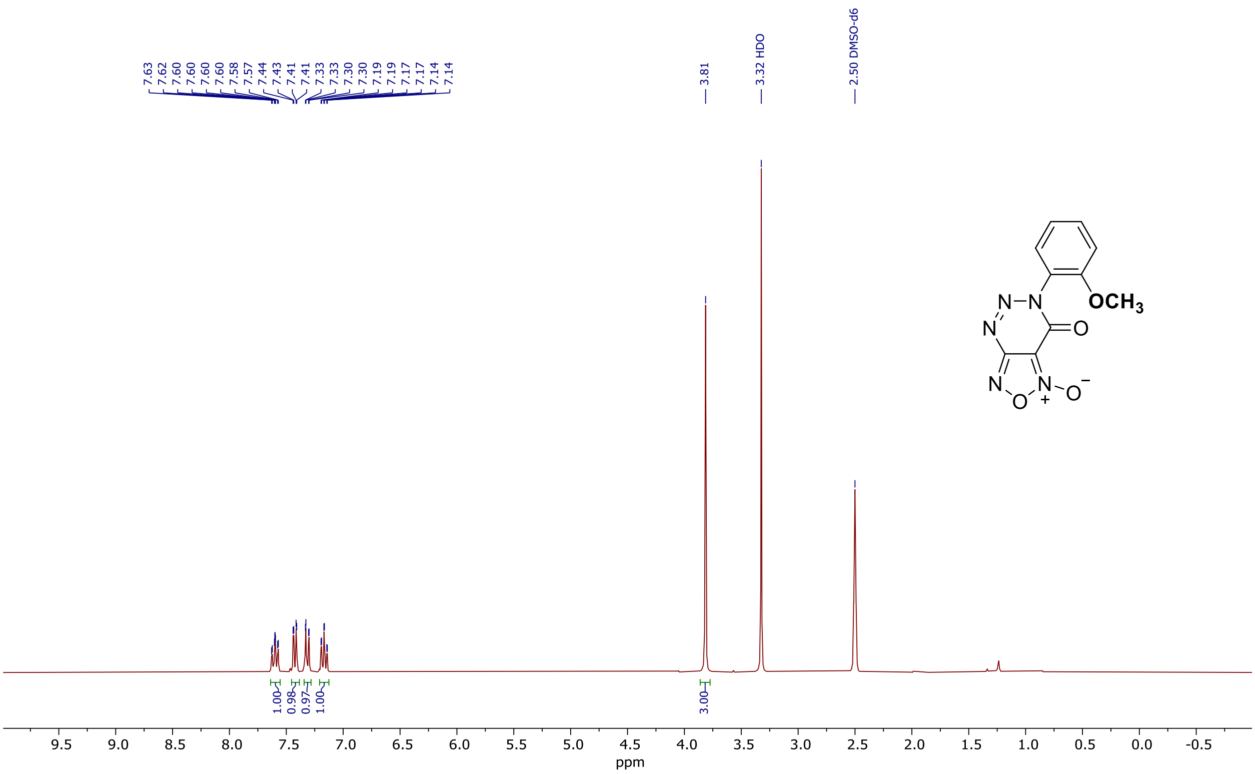

^{13}C {^{1}H} NMR spectrum (76 MHz) of **1e** in DMSO-d_6

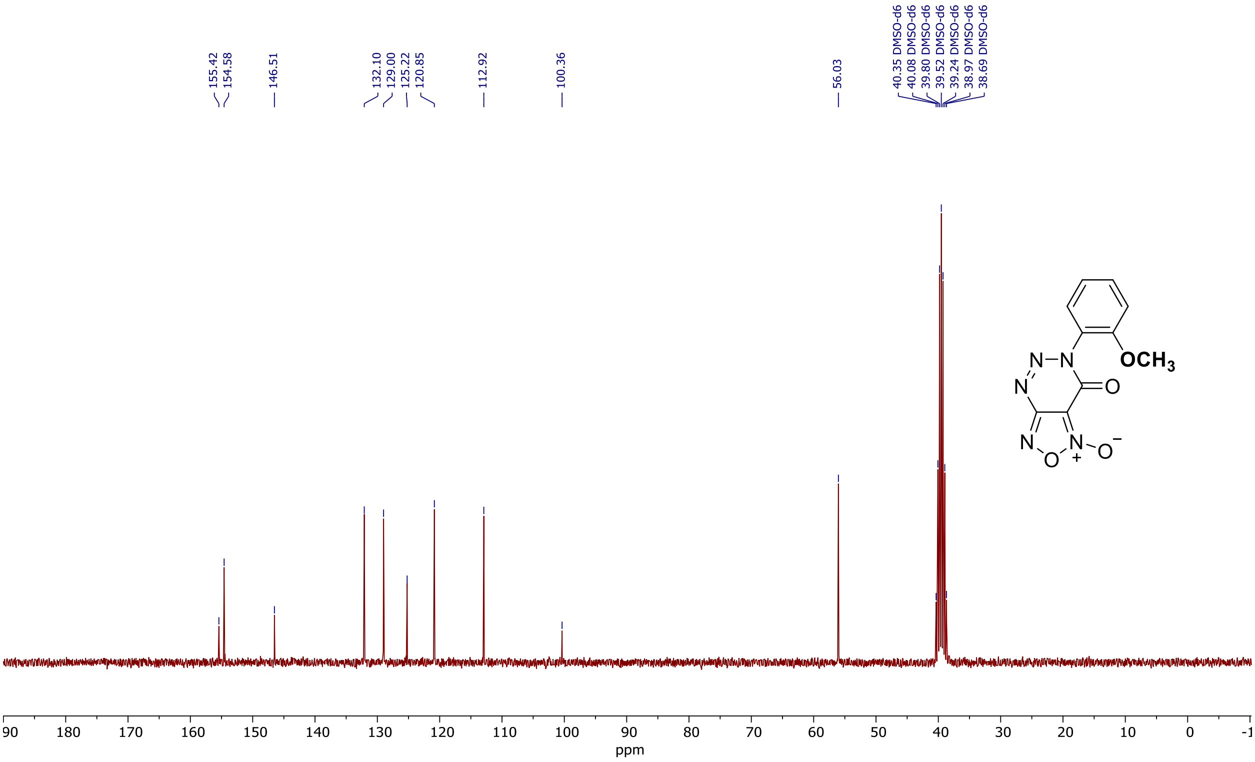

^1H NMR spectrum (300 MHz) of **1f** in DMSO-d_6

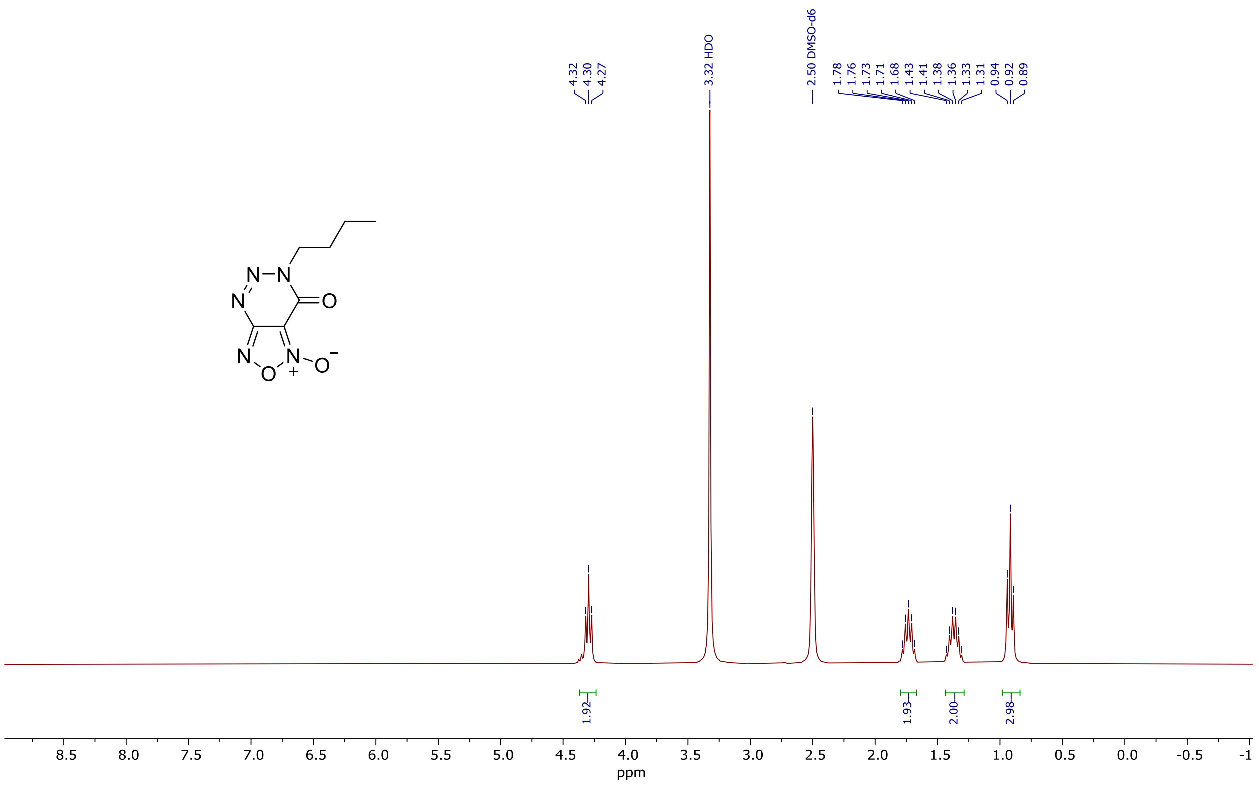

^{13}C {^1H} NMR spectrum (76 MHz) of **1f** in DMSO-d_6

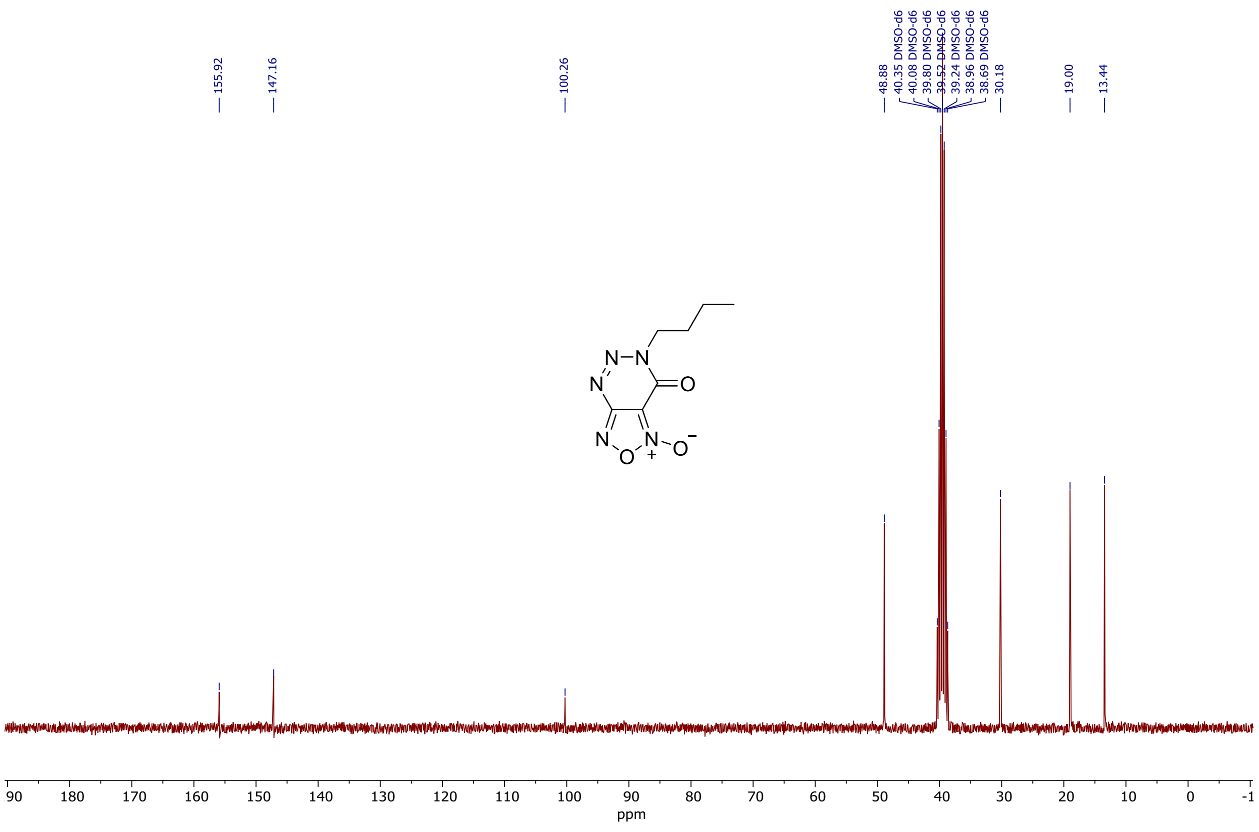

^{1}H NMR spectrum (300 MHz) of **1g** in DMSO-d_6

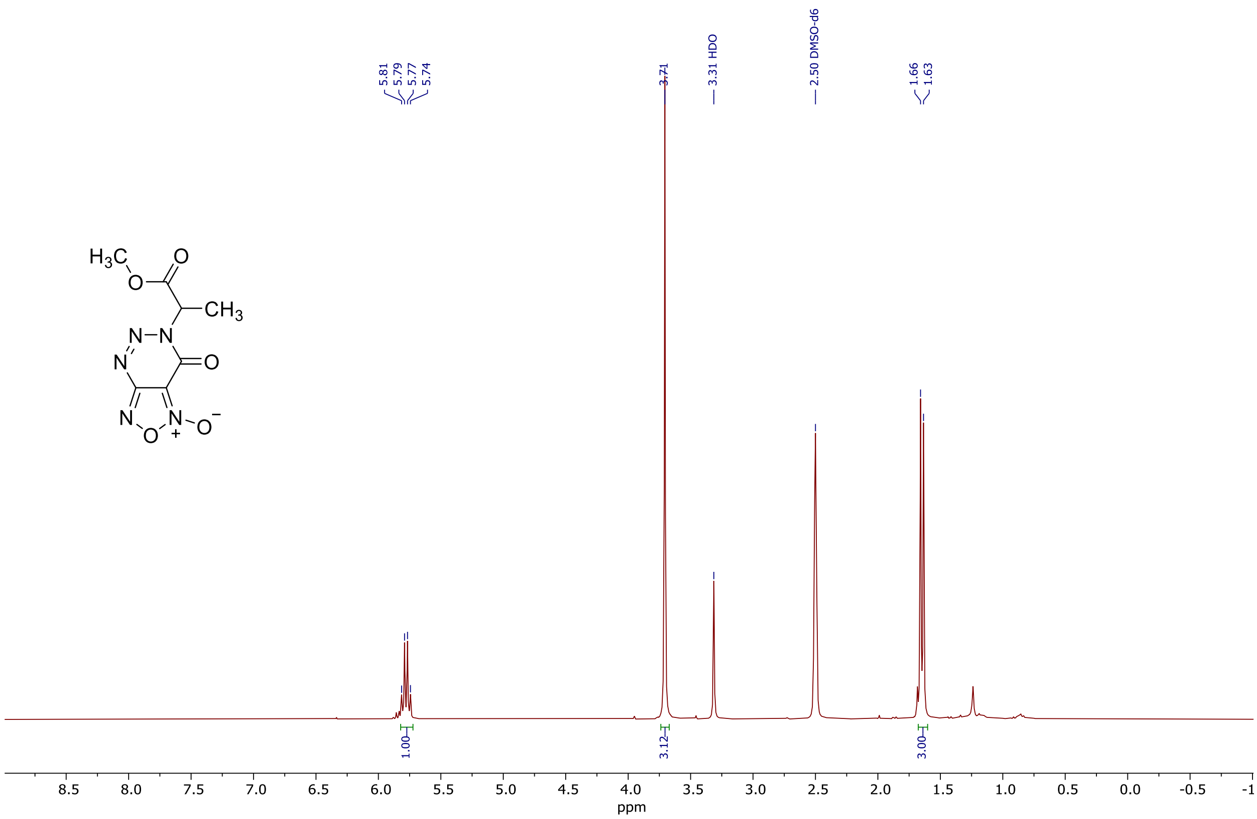

^{13}C {^{1}H} NMR spectrum (76 MHz) of **1g** in DMSO-d_6

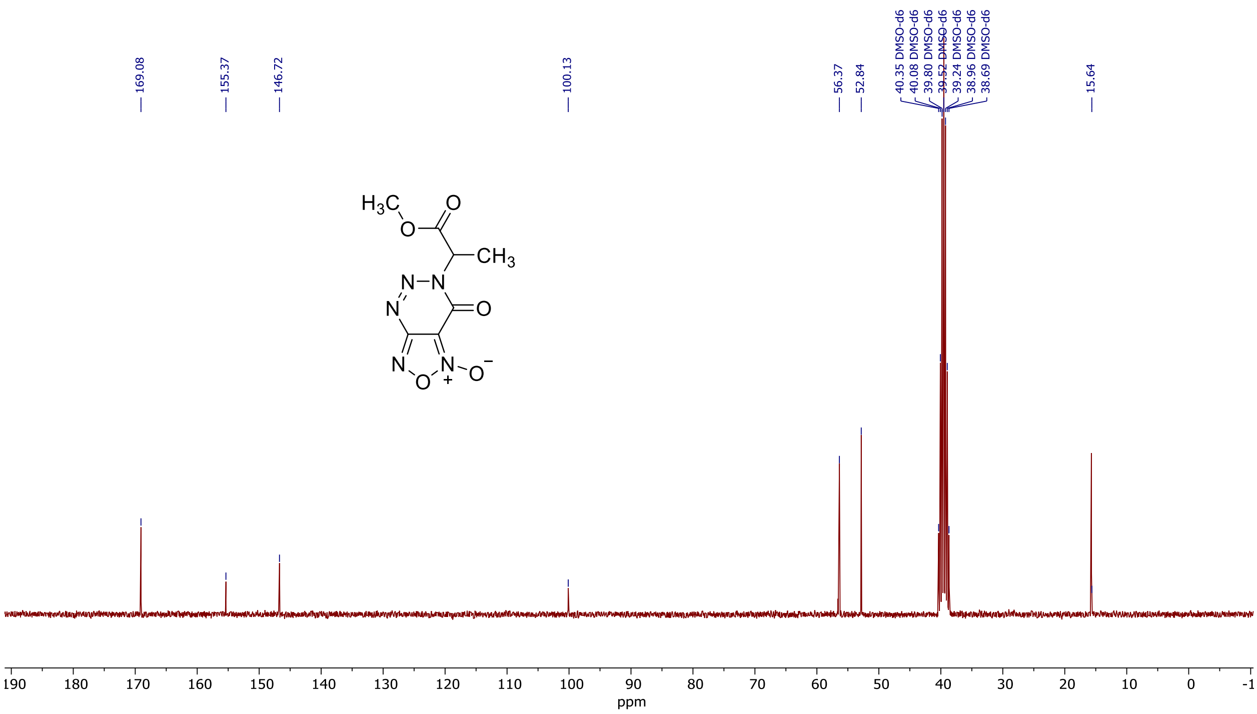

^{1}H NMR spectrum (300 MHz) of **1h** in DMSO-d_6

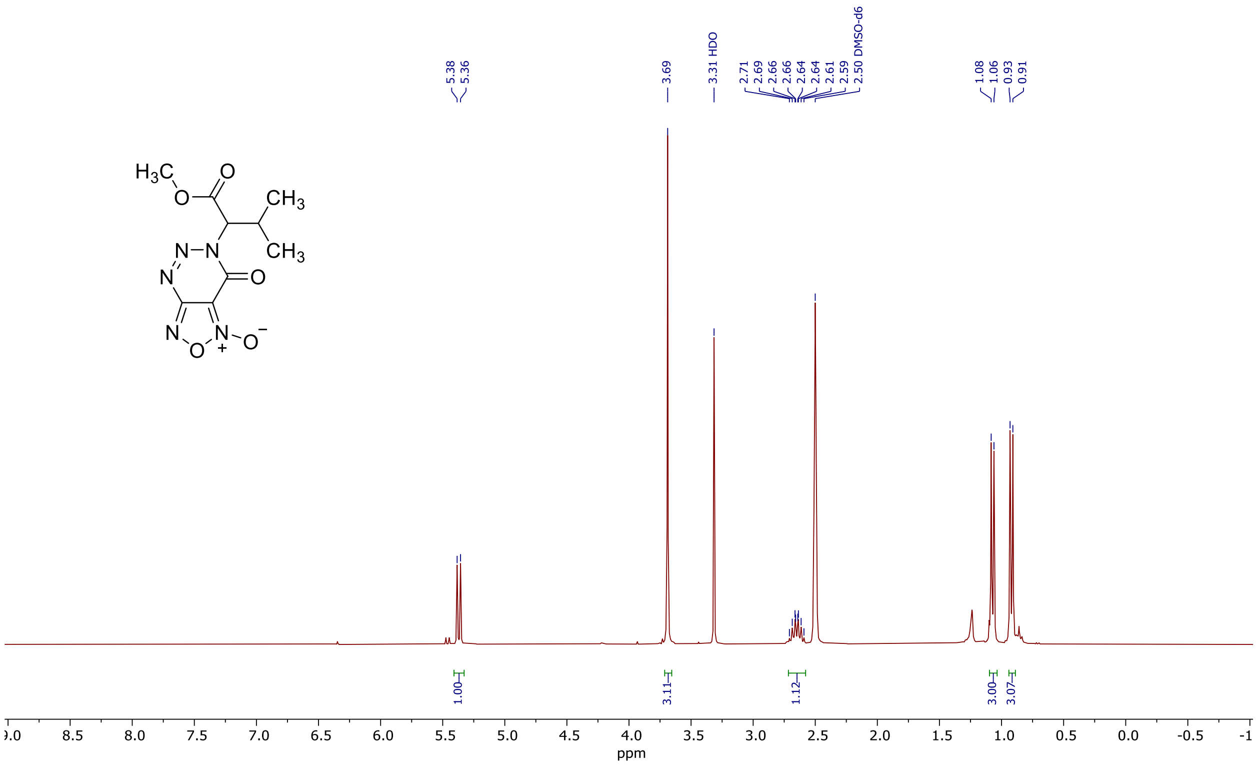

^{13}C {^{1}H} NMR spectrum (76 MHz) of **1h** in DMSO-d_6

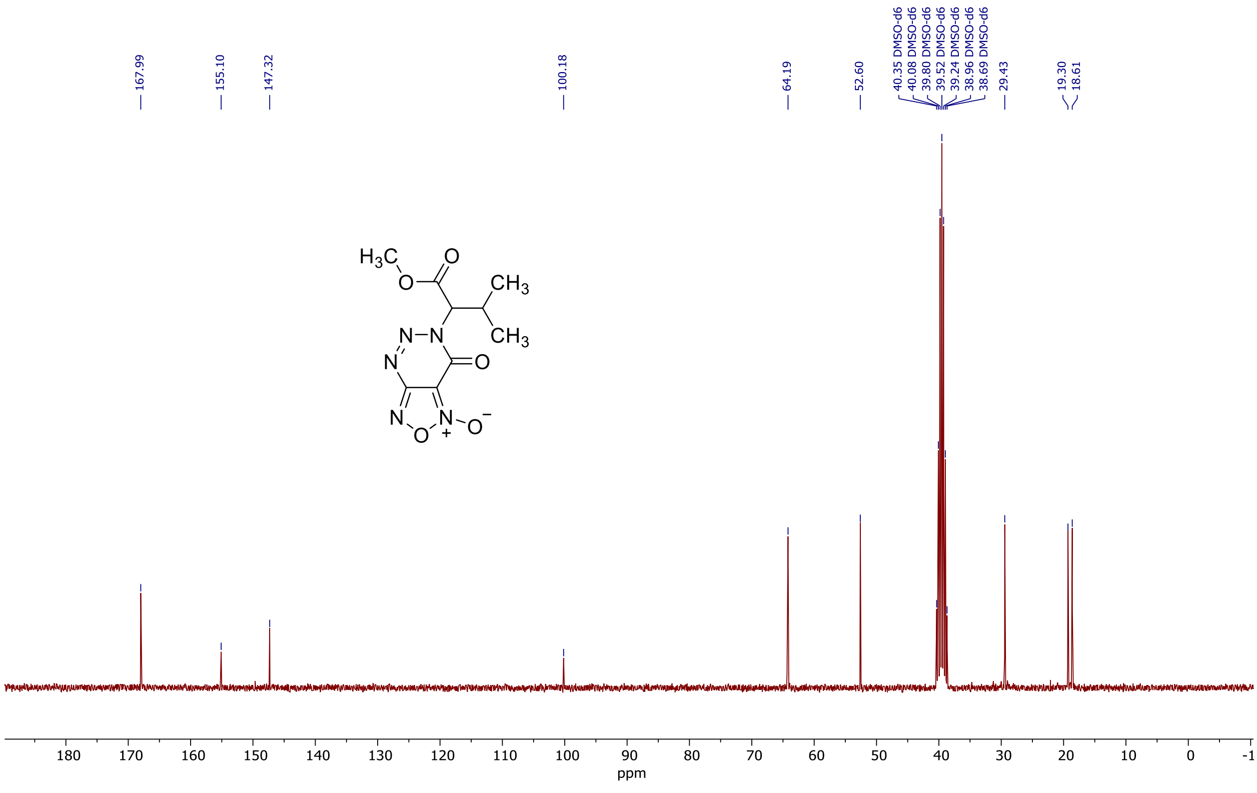

^{1}H NMR spectrum (300 MHz) of **7a** in DMSO-d_6

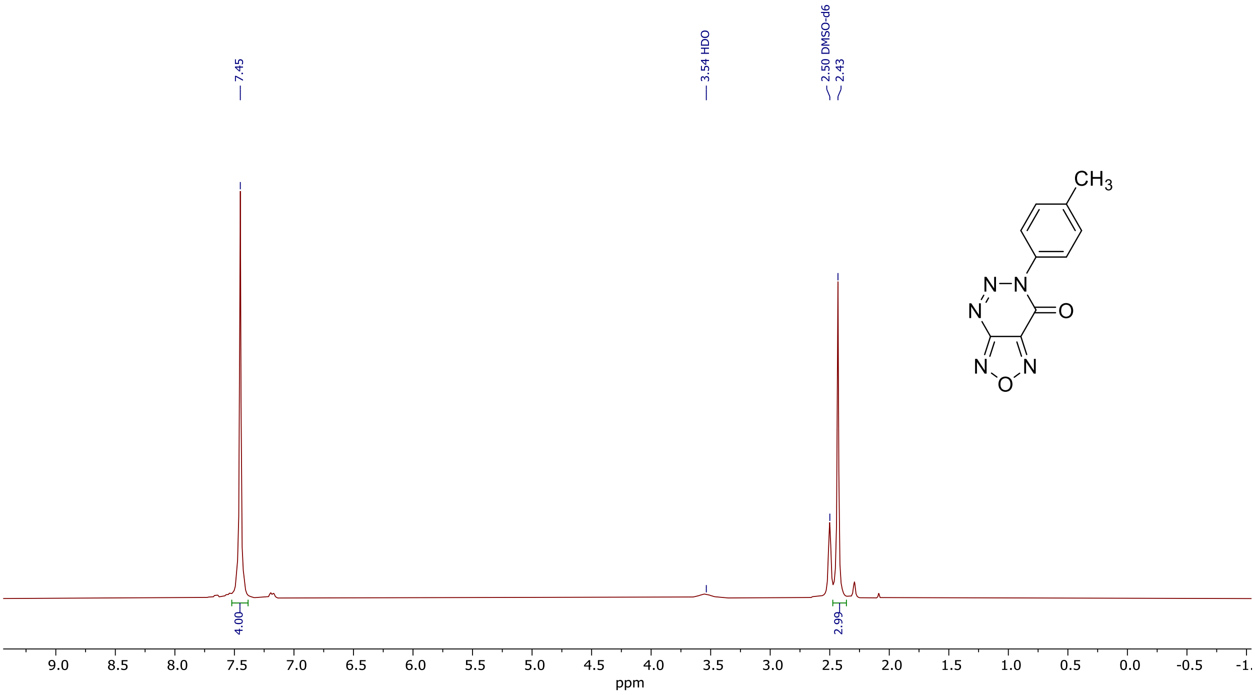

^{13}C {^{1}H} NMR spectrum (76 MHz) of **7a** in DMSO-d_6

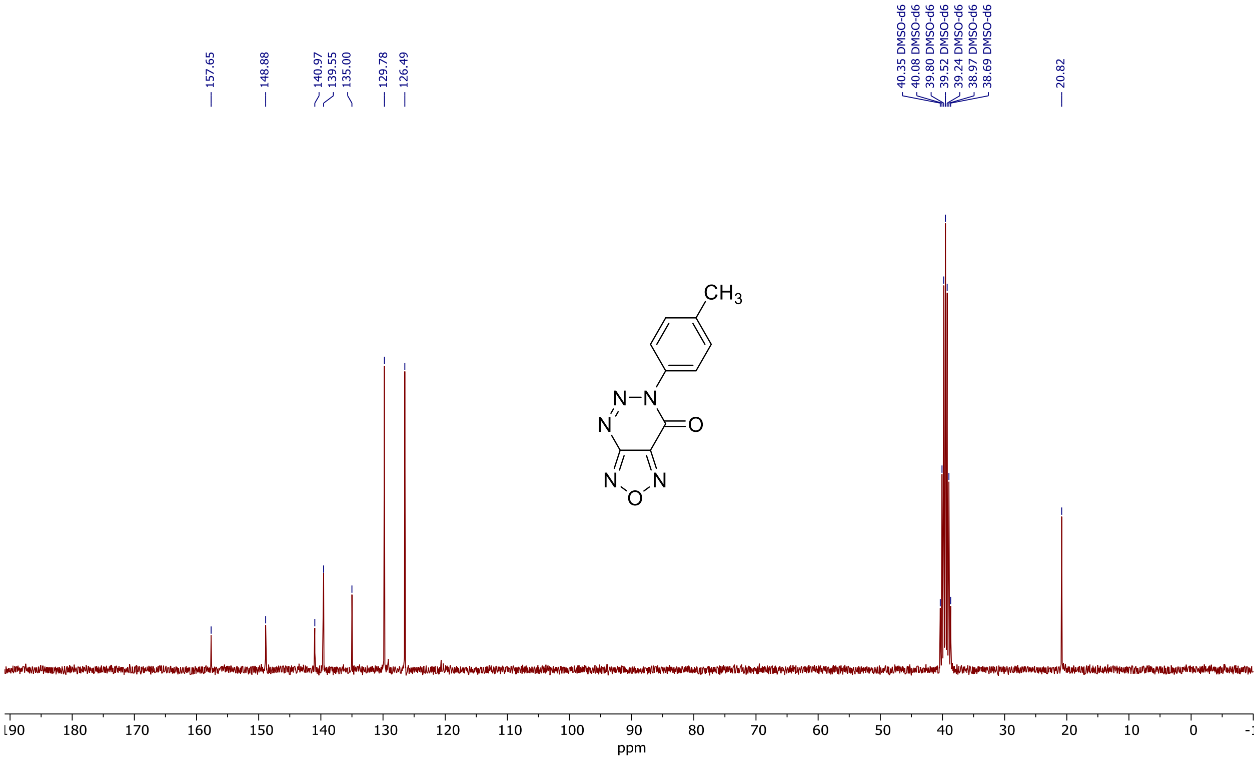

^{1}H NMR spectrum (300 MHz) of **7b** in DMSO-d_6

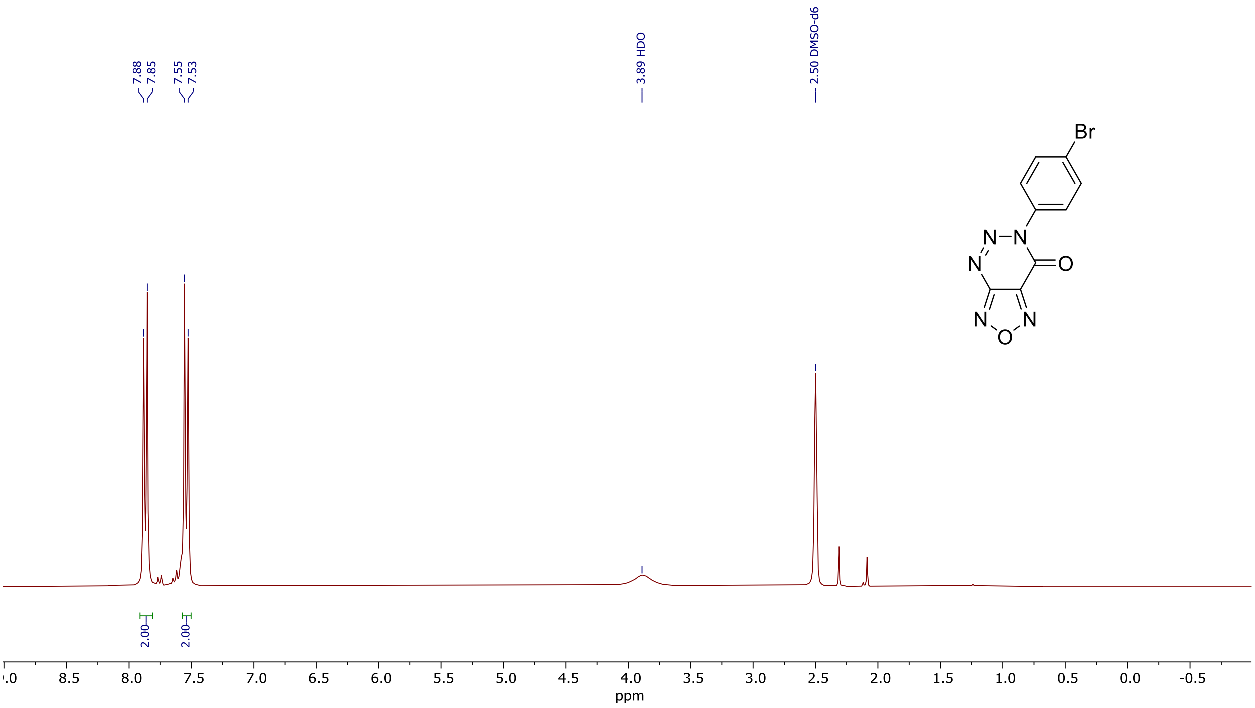

$^{13}C\{^{1}H\}$ NMR spectrum (76 MHz) of **7b** in DMSO-d_6

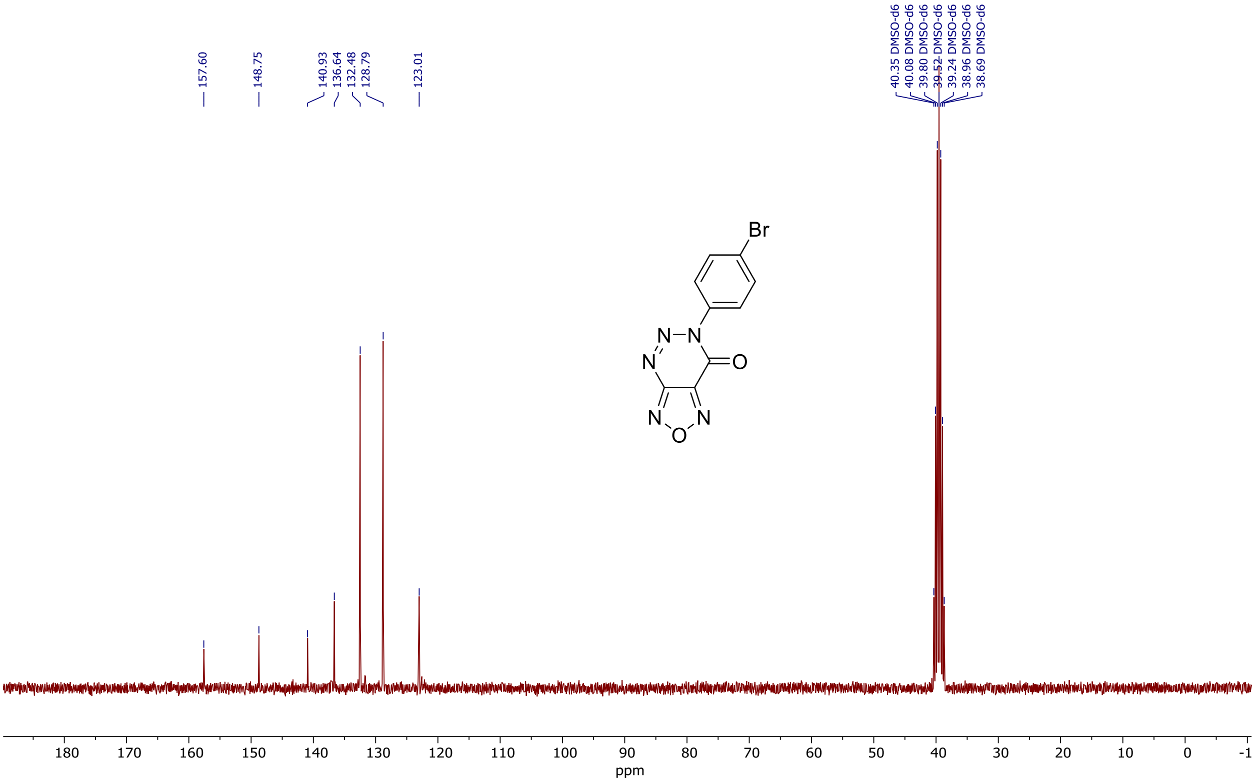

^{1}H NMR spectrum (300 MHz) of **7c** in DMSO-d_6

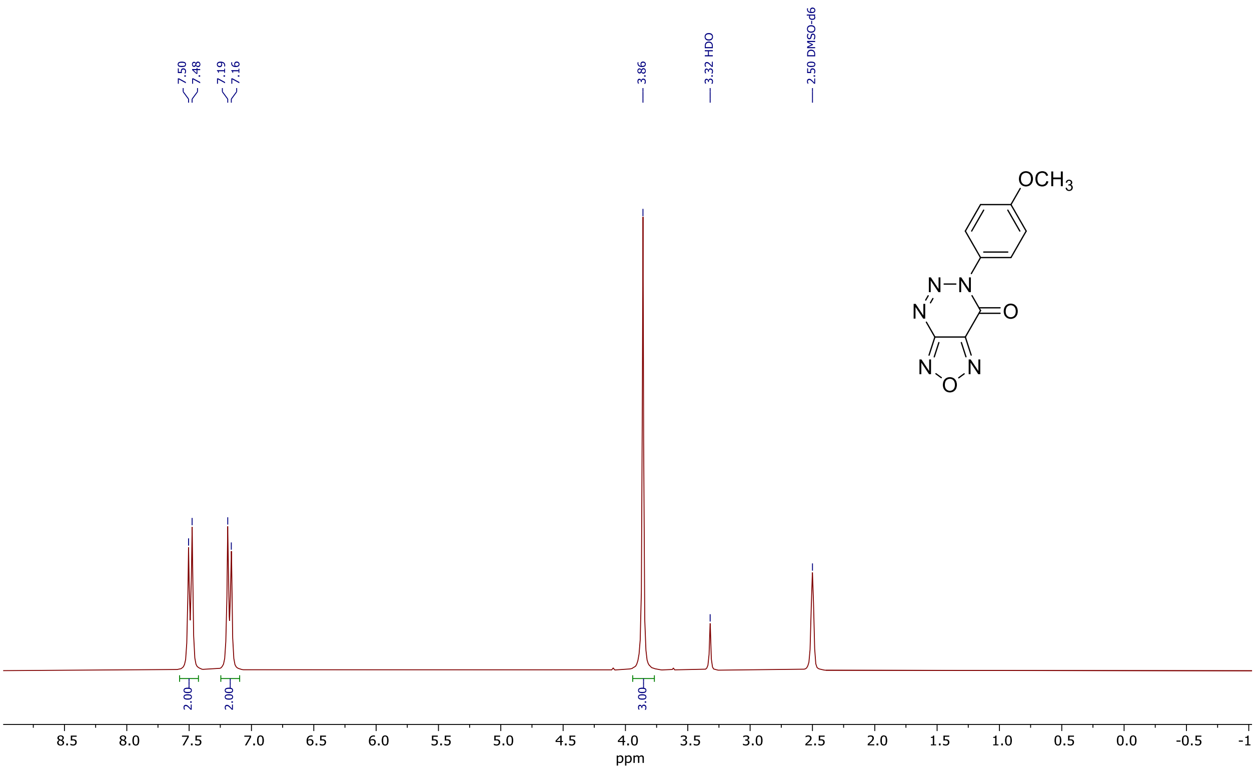

^{13}C {^{1}H} NMR spectrum (76 MHz) of **7c** in DMSO-d_6

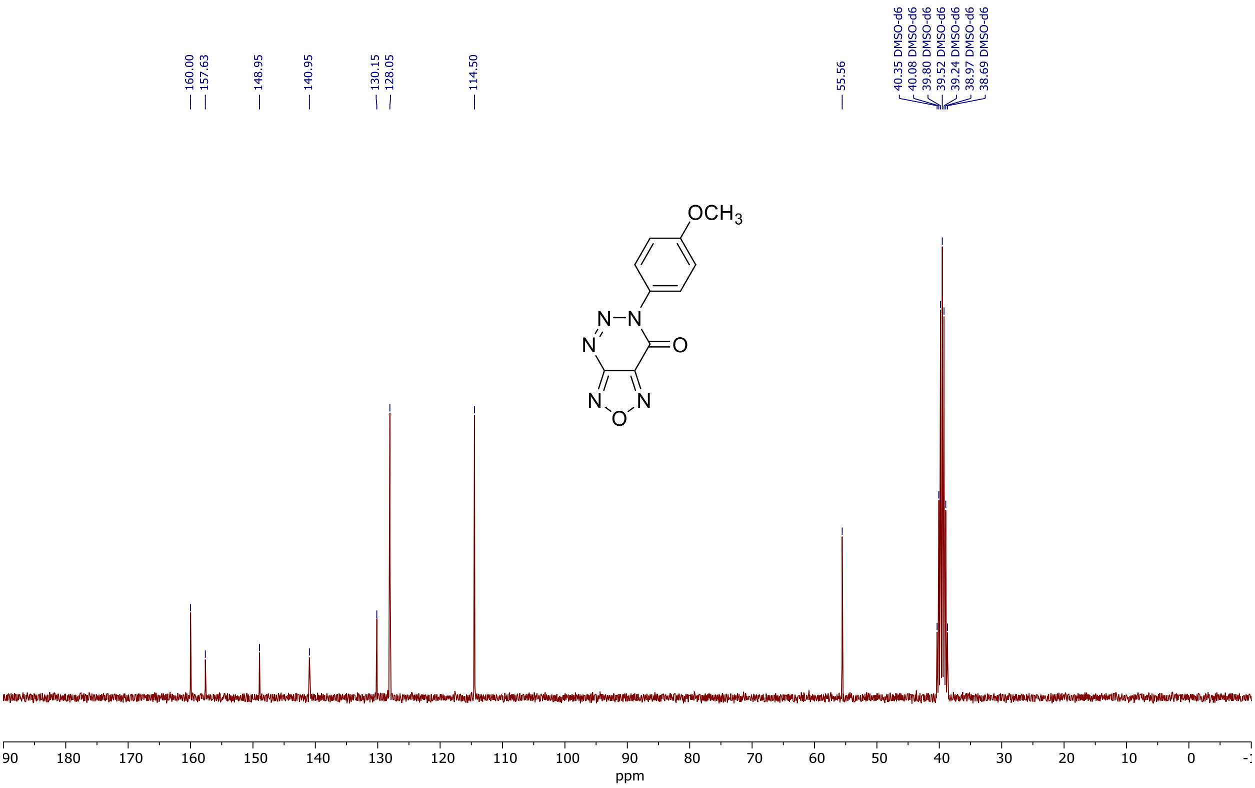

^{1}H NMR spectrum (300 MHz) of **7d** in DMSO-d_6

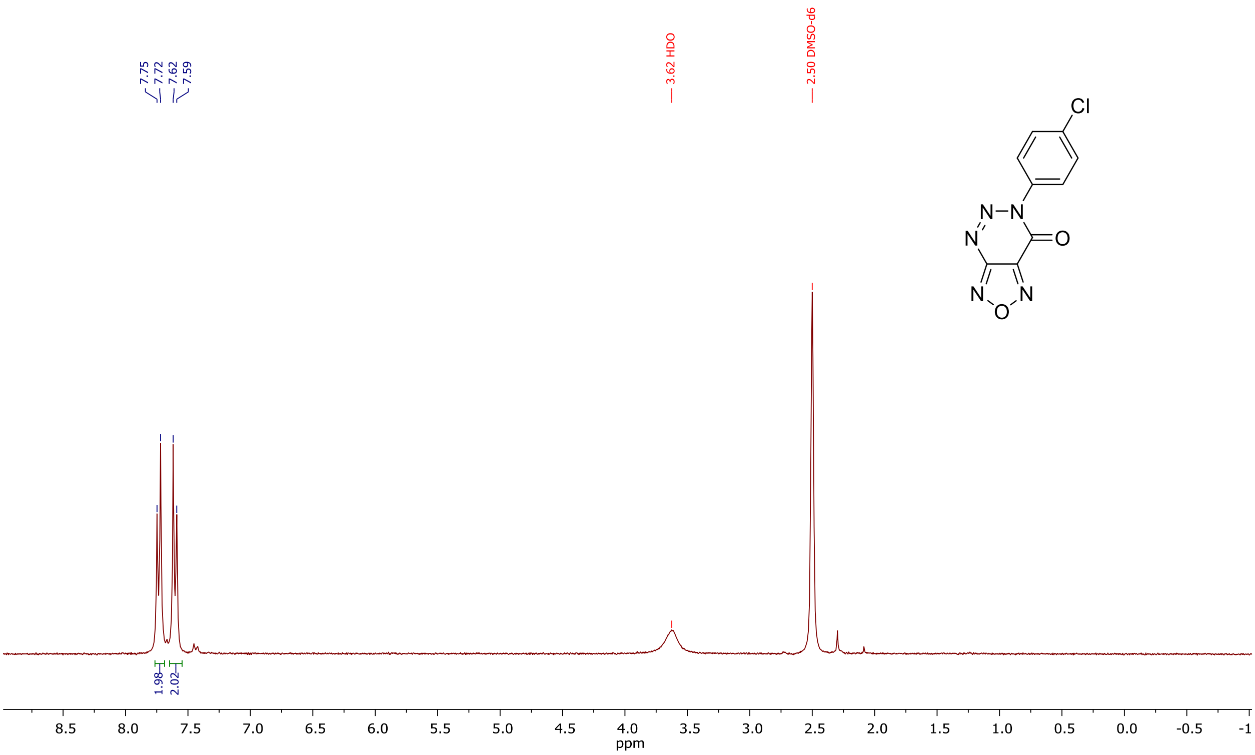

^{13}C {^{1}H} NMR spectrum (76 MHz) of **7d** in DMSO-d_6

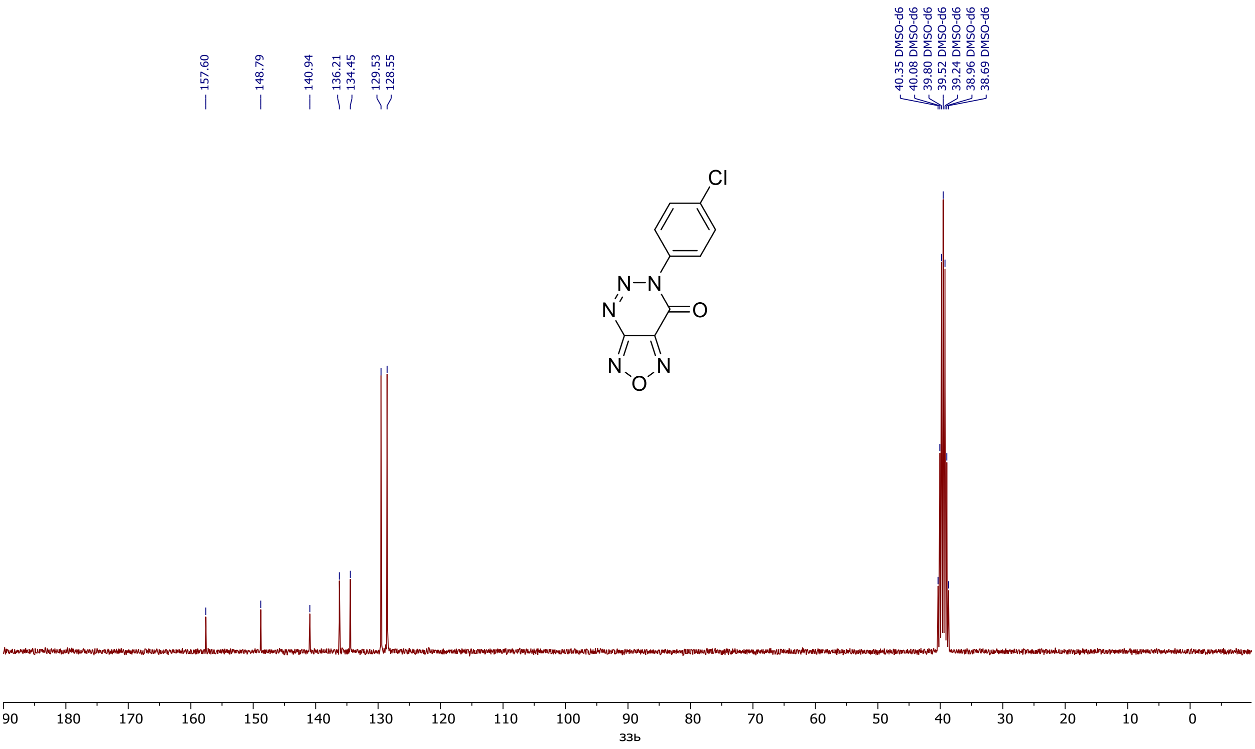

^{1}H NMR spectrum (300 MHz) of **7e** in DMSO-d_6

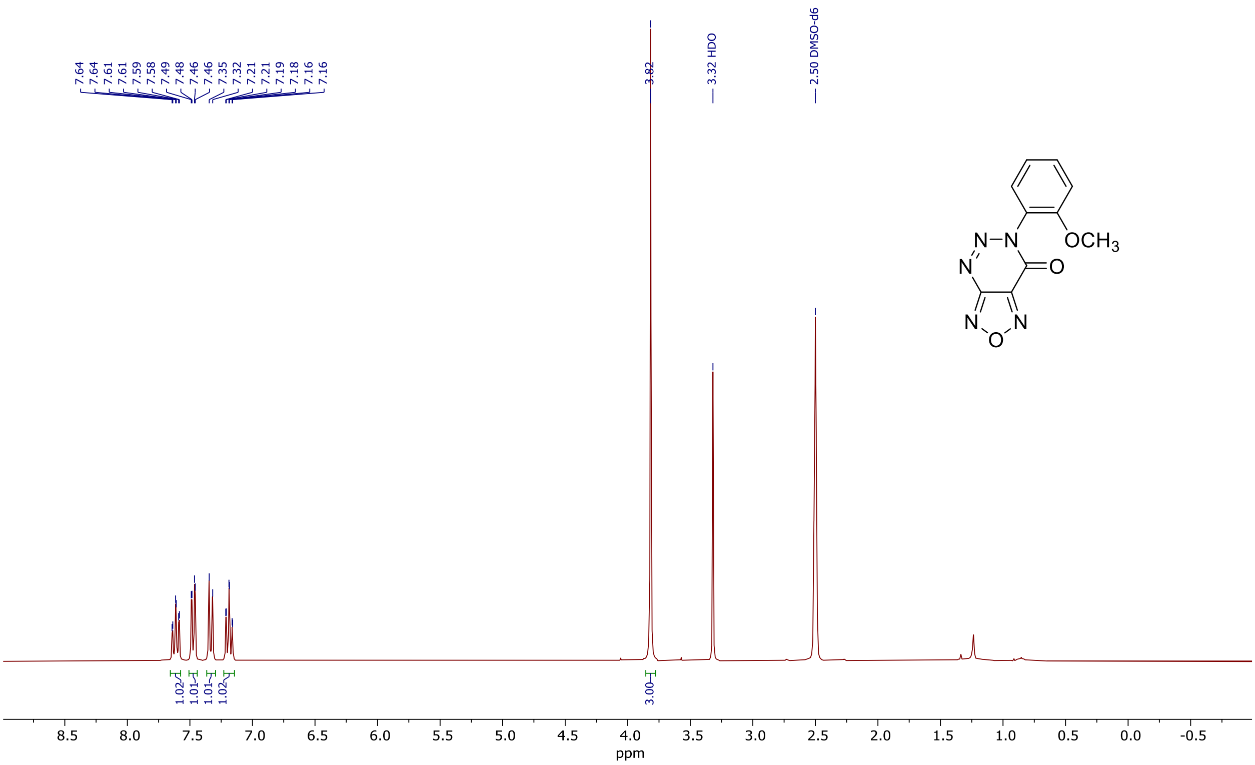

^{13}C {^{1}H} NMR spectrum (76 MHz) of **7e** in DMSO-d_6

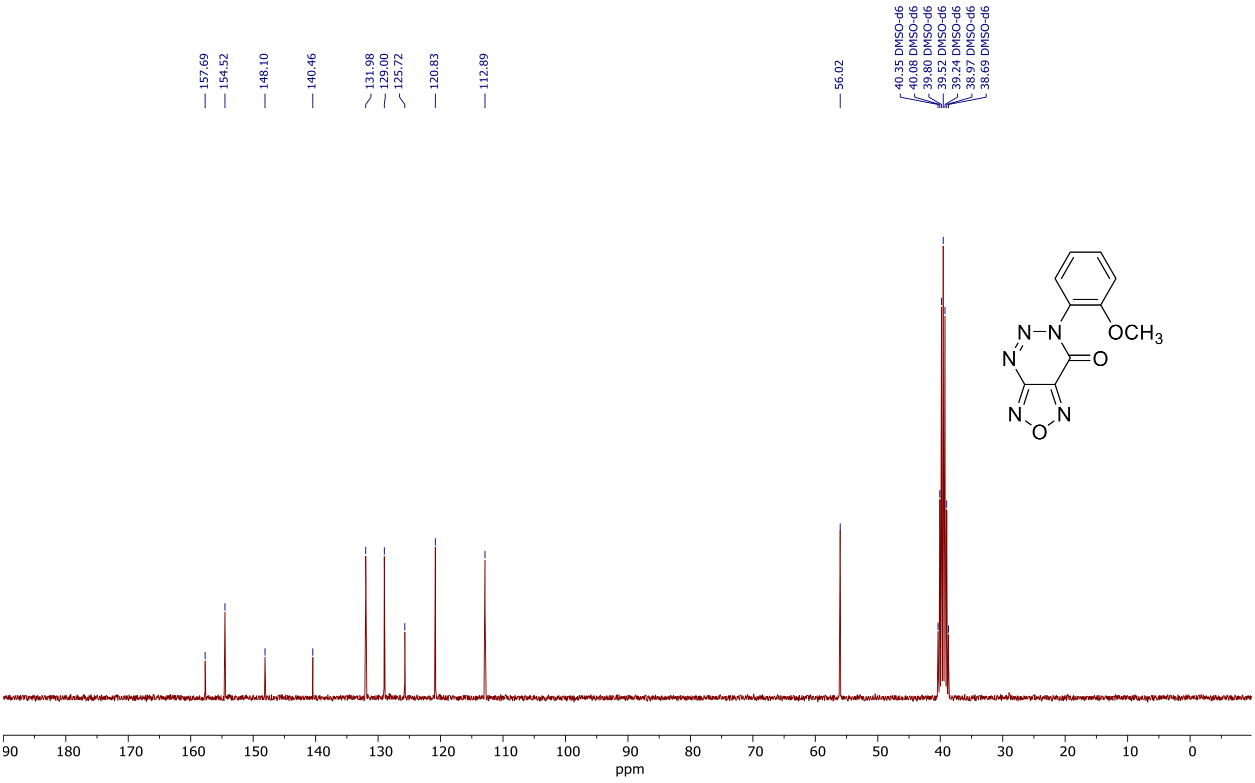

^{1}H NMR spectrum (300 MHz) of **7f** in DMSO-d_6

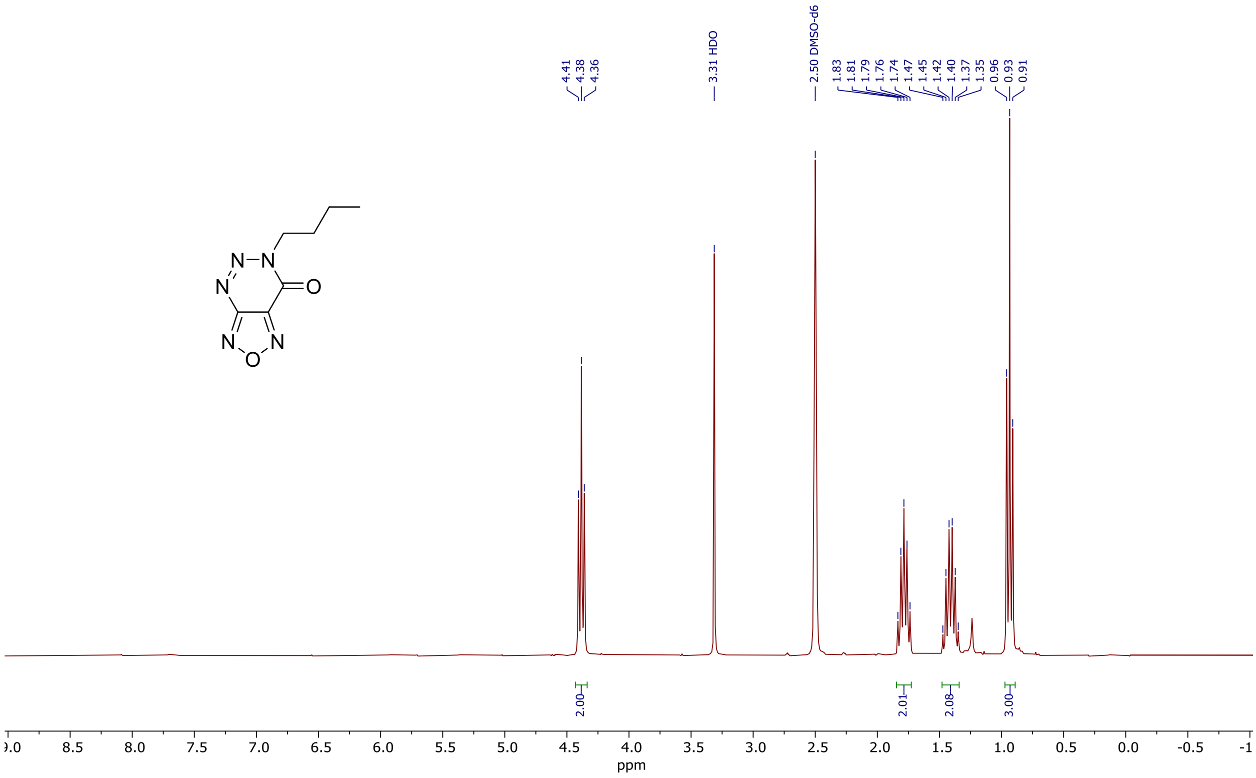

^{13}C {^{1}H} NMR spectrum (76 MHz) of **7f** in DMSO-d_6

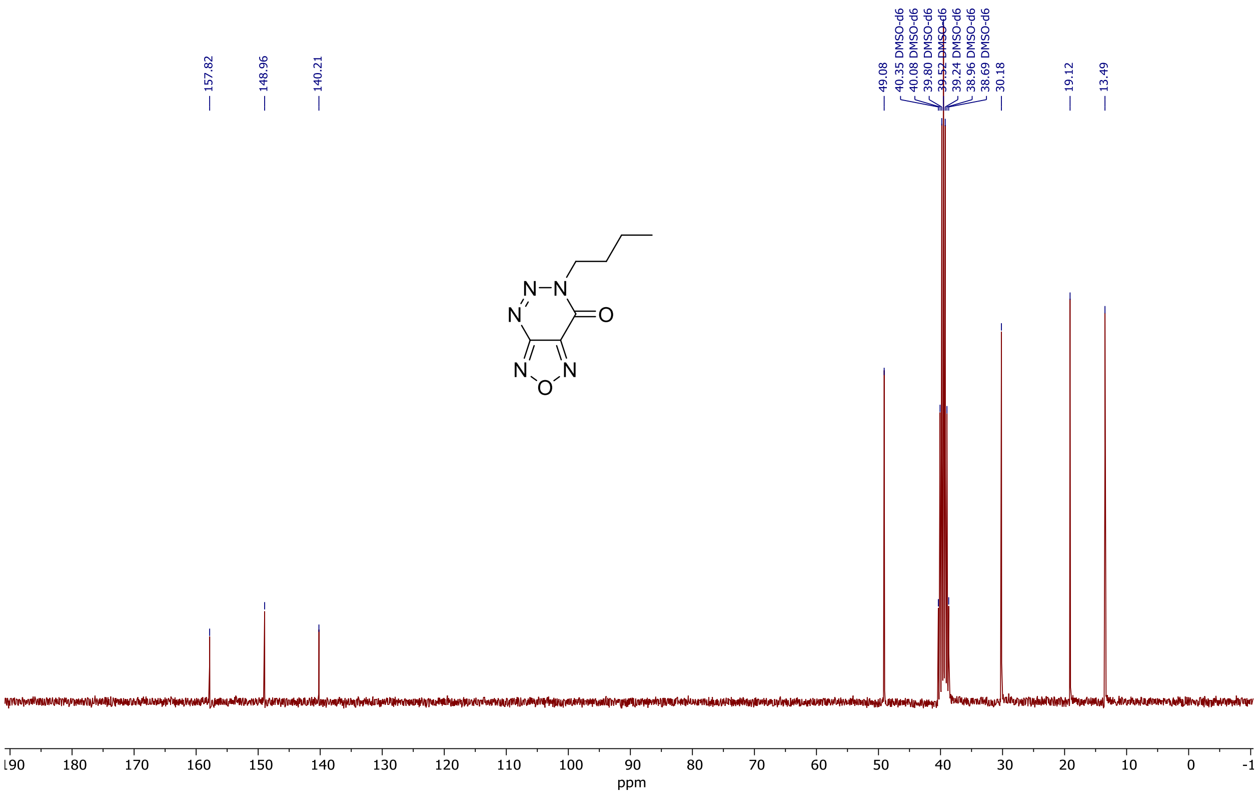

^{1}H NMR spectrum (300 MHz) of **7g** in DMSO-d_6

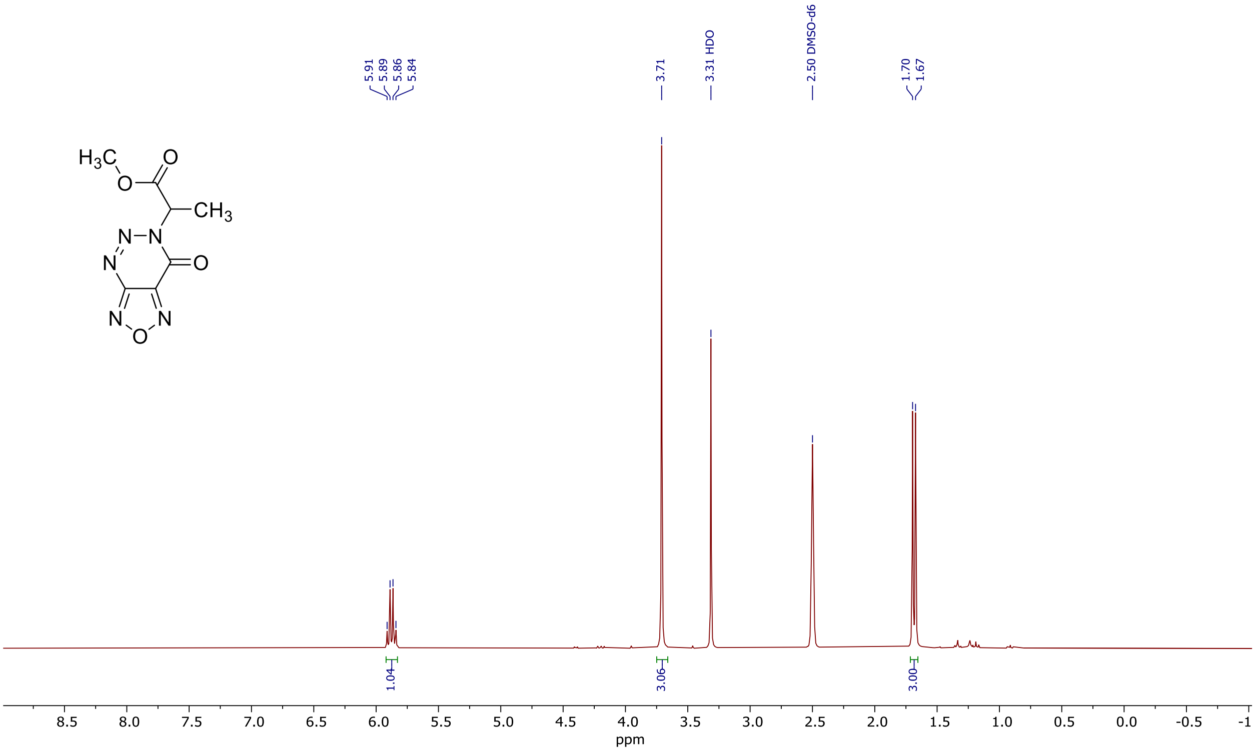

^{13}C {^{1}H} NMR spectrum (76 MHz) of **7g** in DMSO-d_6

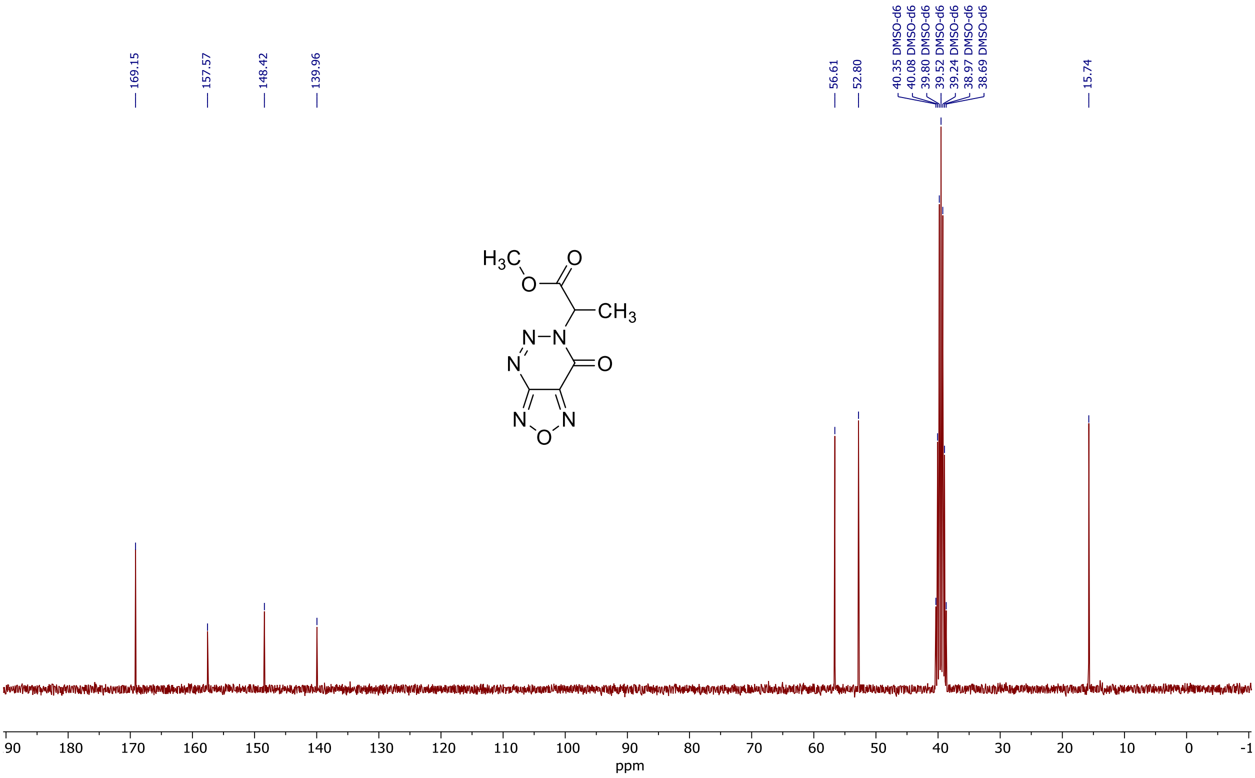

^{1}H NMR spectrum (300 MHz) of **7h** in DMSO-d_6

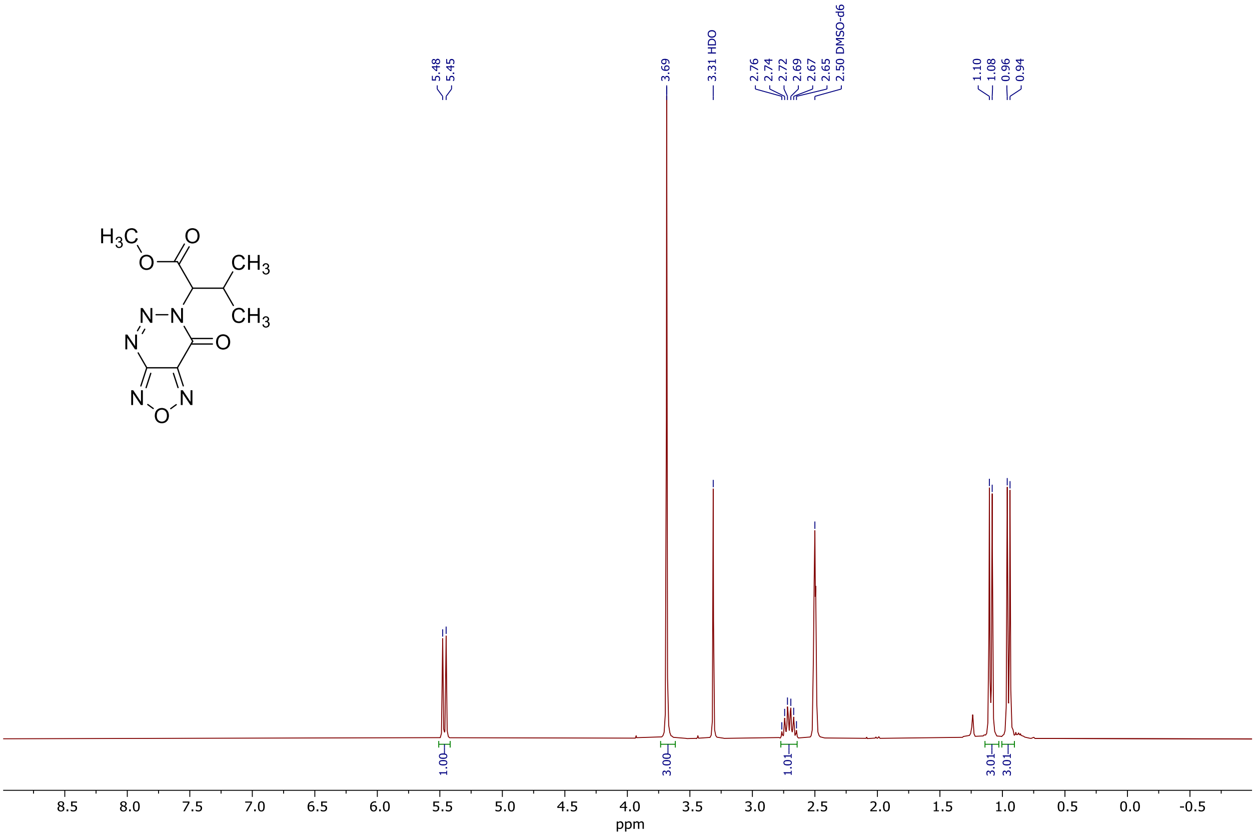

^{13}C {^{1}H} NMR spectrum (76 MHz) of **7h** in DMSO-d_6

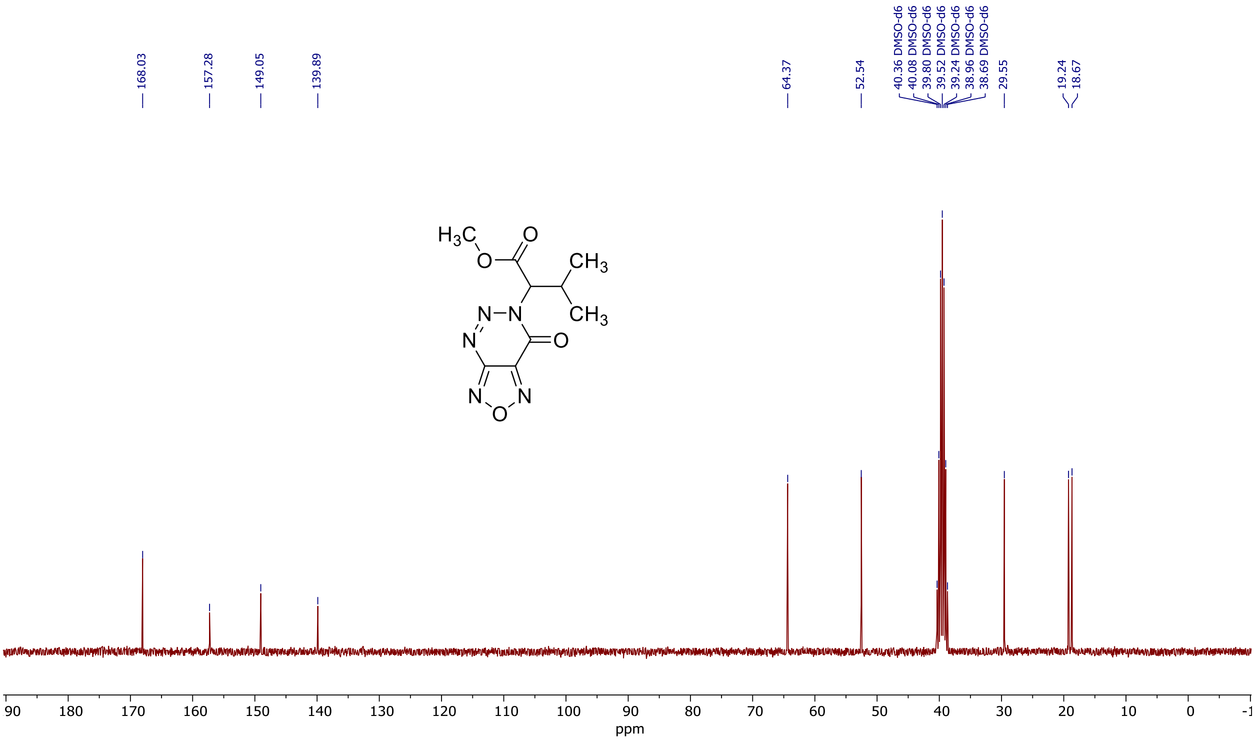

7. ^{15}N NMR spectra for compounds 2a, 1a and 8

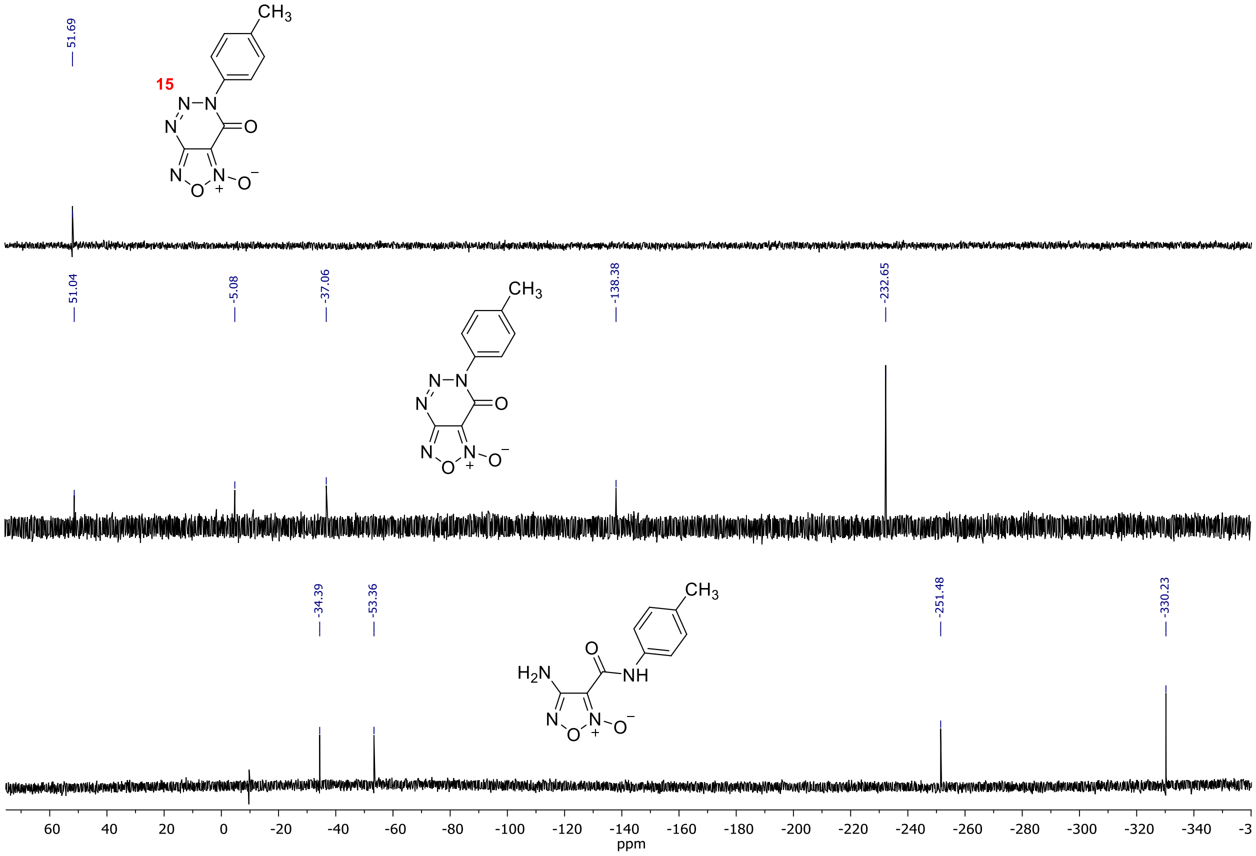

8. X-ray crystallographic data and refinement details for compound 1b

X-ray diffraction data were collected at 100 K on a Rigaku Synergy S diffractometer equipped with a HyPix6000HE area-detector (kappa geometry, shutterless ω-scan technique), using monochromatized Cu K_{α}-radiation. The intensity data were integrated and corrected for absorption and decay by the CrysAlisPro program.[1] The structure was solved by direct methods using SHELXT[2] and refined on F^2 using SHELXL-2018[3] in the OLEX2 program.[4] Positions of all atoms were found from the electron density-difference map. Atoms were refined with individual anisotropic (non-hydrogen atoms) or isotropic (hydrogen atoms) displacement parameters.

1. CrysAlisPro. Version 1.171.42. *Rigaku Oxford Diffraction*, **2022**.
2. Sheldrick, G. M. SHELXT - Integrated space-group and crystal-structure determination. *Acta Cryst.* **2015**, A71(1), 3-8. http://doi.org/10.1107/S2053273314026370
3. Sheldrick, G. M. Crystal structure refinement with SHELXL. *Acta Cryst.* **2015**, C71(1), 3-8. http://doi.org/10.1107/S2053229614024218
4. Dolomanov O.V.; Bourhis L.J.; Gildea R.J.; Howard J.A.K.; Puschmann H. OLEX2: a complete structure solution, refinement and analysis program. *J. Appl. Cryst.* **2009**, 42(2), 339-341. http://doi.org/10.1107/S0021889808042726

Table S1. Crystal data and structure refinement for **1b**.

Identification code	**1b**	
Empirical formula	C9 H4 Br N5 O3	
Formula weight	310.08	
Temperature	99.98(10) K	
Wavelength	1.54184 Å	
Crystal system	Orthorhombic	
Space group	Pbca	
Unit cell dimensions	a = 10.45440(12) Å	a= 90°.
	b = 7.81129(7) Å	b= 90°.
	c = 25.5530(3) Å	g = 90°.
Volume	2086.72(4) $Å^3$	
Z	8	
Density (calculated)	1.974 g/cm^3	
Absorption coefficient	5.516 mm^{-1}	
F(000)	1216	
Crystal size	0.14 x 0.07 x 0.02 mm^3	
Theta range for data collection	3.459 to 79.944°.	
Index ranges	-12<=h<=13, -9<=k<=5, -31<=l<=32	
Reflections collected	13694	
Independent reflections	2264 [R(int) = 0.0206]	
Observed reflections	2160	
Completeness to theta = 67.684°	100.0 %	
Absorption correction	Gaussian	
Max. and min. transmission	1.000 and 0.663	
Refinement method	Full-matrix least-squares on F^2	
Data / restraints / parameters	2264 / 0 / 179	
Goodness-of-fit on F^2	1.132	
Final R indices [I>2sigma(I)]	R1 = 0.0227, wR2 = 0.0625	
R indices (all data)	R1 = 0.0236, wR2 = 0.0631	
Largest diff. peak and hole	0.345 and -0.481 e.$Å^{-3}$	
CCDC	2363621	

Table S2. Atomic coordinates (x 10^4) and equivalent isotropic displacement parameters ($Å^2$x 10^3) for **1b**. U(eq) is defined as one third of the trace of the orthogonalized U^{ij} tensor.

	x	y	z	U(eq)
O(1)	2306(1)	3633(2)	8286(1)	22(1)
N(2)	3144(1)	4486(2)	8087(1)	16(1)
O(3)	4082(1)	5345(2)	8434(1)	20(1)
N(4)	4974(2)	6157(2)	8128(1)	20(1)
C(5)	4635(2)	5851(2)	7644(1)	16(1)
N(6)	5326(1)	6398(2)	7213(1)	18(1)
N(7)	4920(1)	5948(2)	6764(1)	16(1)
N(8)	3835(1)	4951(2)	6706(1)	15(1)
C(9)	3044(2)	4290(2)	7107(1)	15(1)
O(10)	2137(1)	3365(2)	7027(1)	19(1)
C(11)	3521(2)	4863(2)	7606(1)	16(1)
C(12)	3605(2)	4485(2)	6167(1)	15(1)
C(13)	2401(2)	4708(2)	5949(1)	18(1)
C(14)	2202(2)	4241(2)	5430(1)	19(1)
C(15)	3212(2)	3577(2)	5143(1)	18(1)
Br(16)	2953(1)	2887(1)	4440(1)	21(1)
C(17)	4424(2)	3388(2)	5359(1)	18(1)
C(18)	4618(2)	3841(2)	5878(1)	17(1)

Table S3. Bond lengths [Å] and angles [°] for **1b**.

O(1)-N(2)	1.212(2)	N(6)-C(5)-C(11)	123.31(15)
N(2)-O(3)	1.4822(19)	N(7)-N(6)-C(5)	117.19(14)
N(2)-C(11)	1.324(2)	N(6)-N(7)-N(8)	121.71(14)
O(3)-N(4)	1.371(2)	N(7)-N(8)-C(9)	127.36(14)
N(4)-C(5)	1.310(2)	N(7)-N(8)-C(12)	112.32(13)
C(5)-N(6)	1.384(2)	C(9)-N(8)-C(12)	120.14(14)
C(5)-C(11)	1.400(2)	N(8)-C(9)-C(11)	109.00(14)
N(6)-N(7)	1.273(2)	O(10)-C(9)-N(8)	123.63(16)
N(7)-N(8)	1.3842(19)	O(10)-C(9)-C(11)	127.35(16)
N(8)-C(9)	1.414(2)	N(2)-C(11)-C(5)	107.82(15)
N(8)-C(12)	1.445(2)	N(2)-C(11)-C(9)	130.48(16)
C(9)-O(10)	1.210(2)	C(5)-C(11)-C(9)	121.38(15)
C(9)-C(11)	1.439(2)	C(13)-C(12)-N(8)	120.08(15)
C(12)-C(13)	1.388(2)	C(18)-C(12)-N(8)	118.17(15)
C(12)-C(18)	1.385(2)	C(18)-C(12)-C(13)	121.74(16)
C(13)-H(13)	0.93(2)	C(12)-C(13)-H(13)	120.1(15)
C(13)-C(14)	1.391(3)	C(12)-C(13)-C(14)	118.99(16)
C(14)-H(14)	0.96(2)	C(14)-C(13)-H(13)	120.9(15)
C(14)-C(15)	1.387(3)	C(13)-C(14)-H(14)	119.3(14)
C(15)-Br(16)	1.8949(17)	C(15)-C(14)-C(13)	119.31(16)
C(15)-C(17)	1.391(2)	C(15)-C(14)-H(14)	121.4(14)
C(17)-H(17)	0.94(2)	C(14)-C(15)-Br(16)	119.97(14)
C(17)-C(18)	1.387(2)	C(14)-C(15)-C(17)	121.52(16)
C(18)-H(18)	0.89(3)	C(17)-C(15)-Br(16)	118.51(13)
O(1)-N(2)-O(3)	118.45(13)	C(15)-C(17)-H(17)	119.5(14)
O(1)-N(2)-C(11)	136.62(16)	C(18)-C(17)-C(15)	119.11(16)
C(11)-N(2)-O(3)	104.89(13)	C(18)-C(17)-H(17)	121.4(14)
N(4)-O(3)-N(2)	108.63(12)	C(12)-C(18)-C(17)	119.31(16)
C(5)-N(4)-O(3)	105.63(14)	C(12)-C(18)-H(18)	121.5(15)
N(4)-C(5)-N(6)	123.65(16)	C(17)-C(18)-H(18)	119.2(15)
N(4)-C(5)-C(11)	113.01(15)		

Table S4. Anisotropic displacement parameters ($Å^2$x 10^3) for **1b**. The anisotropic displacement factor exponent takes the form: $-2p^2[h^2 a^{*2}U^{11} + ... + 2 h k a^* b^* U^{12}]$

	U^{11}	U^{22}	U^{33}	U^{23}	U^{13}	U^{12}
O(1)	20(1)	24(1)	20(1)	3(1)	4(1)	-2(1)
N(2)	16(1)	17(1)	16(1)	0(1)	0(1)	1(1)
O(3)	20(1)	25(1)	15(1)	-2(1)	-1(1)	0(1)
N(4)	18(1)	22(1)	20(1)	-2(1)	0(1)	0(1)
C(5)	14(1)	15(1)	19(1)	-2(1)	-1(1)	2(1)
N(6)	16(1)	18(1)	20(1)	-2(1)	1(1)	-2(1)
N(7)	14(1)	16(1)	19(1)	-2(1)	1(1)	-2(1)
N(8)	14(1)	15(1)	15(1)	-1(1)	0(1)	-1(1)
C(9)	14(1)	13(1)	17(1)	1(1)	1(1)	1(1)
O(10)	17(1)	20(1)	20(1)	1(1)	-2(1)	-5(1)
C(11)	16(1)	14(1)	16(1)	1(1)	-1(1)	2(1)
C(12)	18(1)	13(1)	14(1)	-1(1)	0(1)	-2(1)
C(13)	15(1)	18(1)	20(1)	0(1)	1(1)	2(1)
C(14)	16(1)	20(1)	20(1)	1(1)	-2(1)	0(1)
C(15)	22(1)	17(1)	14(1)	0(1)	-1(1)	-2(1)
Br(16)	25(1)	25(1)	14(1)	-2(1)	-2(1)	-4(1)
C(17)	17(1)	19(1)	17(1)	0(1)	3(1)	0(1)
C(18)	15(1)	19(1)	17(1)	2(1)	-1(1)	0(1)

Table S5. Hydrogen coordinates (x 10^4) and isotropic displacement parameters (Å^{2}x 10 ^{3}) for **1b**.

	x	y	z	U(eq)
H(13)	1740(20)	5180(30)	6147(9)	21(6)
H(14)	1370(20)	4390(30)	5279(9)	21(5)
H(17)	5090(20)	2960(30)	5153(9)	17(5)
H(18)	5390(20)	3700(30)	6019(9)	23(6)

Table S6. Torsion angles [°] for **1b**.

O(1)-N(2)-O(3)-N(4)	176.94(14)	N(8)-C(9)-C(11)-N(2)	-175.27(17)
O(1)-N(2)-C(11)-C(5)	-175.98(19)	N(8)-C(9)-C(11)-C(5)	-2.6(2)
O(1)-N(2)-C(11)-C(9)	-2.6(3)	N(8)-C(12)-C(13)-C(14)	-179.65(15)
N(2)-O(3)-N(4)-C(5)	0.16(17)	N(8)-C(12)-C(18)-C(17)	-179.81(15)
O(3)-N(2)-C(11)-C(5)	1.45(17)	C(9)-N(8)-C(12)-C(13)	53.5(2)
O(3)-N(2)-C(11)-C(9)	174.85(16)	C(9)-N(8)-C(12)-C(18)	-127.40(17)
O(3)-N(4)-C(5)-N(6)	-177.34(15)	O(10)-C(9)-C(11)-N(2)	3.3(3)
O(3)-N(4)-C(5)-C(11)	0.78(19)	O(10)-C(9)-C(11)-C(5)	175.89(17)
N(4)-C(5)-N(6)-N(7)	177.00(16)	C(11)-N(2)-O(3)-N(4)	-1.05(16)
N(4)-C(5)-C(11)-N(2)	-1.5(2)	C(11)-C(5)-N(6)-N(7)	-0.9(2)
N(4)-C(5)-C(11)-C(9)	-175.62(15)	C(12)-N(8)-C(9)-O(10)	-2.1(2)
C(5)-N(6)-N(7)-N(8)	-0.1(2)	C(12)-N(8)-C(9)-C(11)	176.51(14)
N(6)-C(5)-C(11)-N(2)	176.62(15)	C(12)-C(13)-C(14)-C(15)	-0.5(3)
N(6)-C(5)-C(11)-C(9)	2.5(3)	C(13)-C(12)-C(18)-C(17)	-0.7(3)
N(6)-N(7)-N(8)-C(9)	-0.5(2)	C(13)-C(14)-C(15)-Br(16)	178.66(13)
N(6)-N(7)-N(8)-C(12)	-175.55(15)	C(13)-C(14)-C(15)-C(17)	-0.9(3)
N(7)-N(8)-C(9)-O(10)	-176.82(16)	C(14)-C(15)-C(17)-C(18)	1.4(3)
N(7)-N(8)-C(9)-C(11)	1.8(2)	C(15)-C(17)-C(18)-C(12)	-0.6(3)
N(7)-N(8)-C(12)-C(13)	-131.02(16)	Br(16)-C(15)-C(17)-C(18)	-178.12(13)
N(7)-N(8)-C(12)-C(18)	48.1(2)	C(18)-C(12)-C(13)-C(14)	1.3(3)

9. X-ray crystallographic data and refinement details for compound 7h

X-ray diffraction data were collected at 100K on a Rigaku Synergy S diffractometer equipped with a HyPix6000HE area-detector (kappa geometry, shutterless ω-scan technique), using monochromatized Cu K_a-radiation. The intensity data were integrated and corrected for absorption and decay by the CrysAlisPro program[1]. The structure was solved by direct methods using SHELXT[2] and refined on F^2 using SHELXL-2018[3] in the OLEX2 program.[4] Positions of all atoms were found from the electron density-difference map. Atoms were refined with individual anisotropic (non-hydrogen atoms) or isotropic (hydrogen atoms) displacement parameters.

1. CrysAlisPro. Version 1.171.42. *Rigaku Oxford Diffraction*, **2022**.
2. Sheldrick, G. M. SHELXT - Integrated space-group and crystal-structure determination. *Acta Cryst.* **2015**, A71(1), 3-8. http://doi.org/10.1107/S2053273314026370
3. Sheldrick, G. M. Crystal structure refinement with SHELXL. *Acta Cryst.* **2015**, C71(1), 3-8. http://doi.org/10.1107/S2053229614024218
4. Dolomanov O.V.; Bourhis L.J.; Gildea R.J.; Howard J.A.K.; Puschmann H. OLEX2: a complete structure solution, refinement and analysis program. *J. Appl. Cryst.* **2009**, 42(2), 339-341. http://doi.org/10.1107/S0021889808042726

Table S7. Crystal data and structure refinement for **7h**.

Identification code	7h	
Empirical formula	C9 H11 N5 O4	
Formula weight	253.23	
Temperature	99.99(10) K	
Wavelength	1.54184 Å	
Crystal system	Trigonal	
Space group	$P3_2$	
Unit cell dimensions	a = 10.47869(11) Å	a= 90°.
	b = 10.47869(11) Å	b= 90°.
	c = 9.09256(11) Å	g = 120°.
Volume	864.63(2) $Å^3$	
Z	3	
Density (calculated)	1.459 g/cm^3	
Absorption coefficient	1.005 mm^{-1}	
F(000)	396	
Crystal size	0.445 x 0.389 x 0.221 mm^3	
Theta range for data collection	4.873 to 79.537°.	
Index ranges	-13<=h<=12, -10<=k<=13, -11<=l<=11	
Reflections collected	10401	
Independent reflections	2446 [R(int) = 0.0238]	
Observed reflections	2441	
Completeness to theta = 67.684°	100.0 %	
Absorption correction	Gaussian	
Max. and min. transmission	1.000 and 0.175	
Refinement method	Full-matrix least-squares on F^2	
Data / restraints / parameters	2446 / 1 / 208	
Goodness-of-fit on F^2	1.052	
Final R indices [I>2sigma(I)]	R1 = 0.0327, wR2 = 0.0823	
R indices (all data)	R1 = 0.0327, wR2 = 0.0824	
Absolute structure parameter	0.02(7)	
Extinction coefficient	0.0076(10)	
Largest diff. peak and hole	0.566 and -0.166 e.$Å^{-3}$	
CCDC	2363622	

Table S8. Atomic coordinates (x 10^4) and equivalent isotropic displacement parameters ($Å^2$x 10^3) for **7h**. U(eq) is defined as one third of the trace of the orthogonalized U^{ij} tensor.

	x	y	z	U(eq)
O(1)	3735(2)	4609(3)	5468(3)	36(1)
N(1)	4746(3)	4400(3)	6250(3)	30(1)
C(1)	5991(3)	5289(3)	5613(3)	25(1)
O(2)	7915(2)	5055(2)	6758(2)	29(1)
N(2)	4357(3)	5613(3)	4368(3)	35(1)
C(2)	5749(3)	6037(3)	4463(3)	28(1)
O(3)	10795(2)	9172(2)	5877(2)	26(1)
N(3)	6815(3)	7065(3)	3516(3)	32(1)
C(3)	7499(3)	5583(3)	5854(3)	24(1)
O(4)	11878(2)	9251(2)	3726(2)	32(1)
N(4)	8102(2)	7304(3)	3698(3)	28(1)
C(4)	9982(3)	6900(3)	4682(3)	19(1)
N(5)	8444(2)	6582(2)	4810(2)	21(1)
C(5)	10175(3)	6121(3)	3350(3)	23(1)
C(6)	11642(3)	6151(4)	3465(3)	31(1)
C(7)	10994(3)	8572(3)	4670(3)	20(1)
C(8)	11706(3)	10761(3)	6025(3)	29(1)
C(9)	8921(3)	4525(3)	3239(3)	31(1)

Table S9. Bond lengths [Å] and angles [°] for **7h**.

O(1)-N(1)	1.382(3)	N(3)-C(2)-C(1)	126.3(3)
O(1)-N(2)	1.359(4)	C(7)-O(3)-C(8)	116.7(2)
N(1)-C(1)	1.300(4)	N(4)-N(3)-C(2)	116.0(2)
C(1)-C(2)	1.405(4)	O(2)-C(3)-C(1)	127.8(2)
C(1)-C(3)	1.467(4)	O(2)-C(3)-N(5)	122.1(2)
O(2)-C(3)	1.189(3)	N(5)-C(3)-C(1)	110.1(2)
N(2)-C(2)	1.298(4)	N(3)-N(4)-N(5)	121.6(2)
C(2)-N(3)	1.395(4)	N(5)-C(4)-H(4)	109(2)
O(3)-C(7)	1.332(3)	N(5)-C(4)-C(5)	111.2(2)
O(3)-C(8)	1.453(3)	N(5)-C(4)-C(7)	108.21(19)
N(3)-N(4)	1.254(3)	C(5)-C(4)-H(4)	107.8(19)
C(3)-N(5)	1.393(3)	C(7)-C(4)-H(4)	106(2)
O(4)-C(7)	1.201(3)	C(7)-C(4)-C(5)	113.9(2)
N(4)-N(5)	1.412(3)	C(3)-N(5)-N(4)	127.9(2)
C(4)-H(4)	0.92(3)	C(3)-N(5)-C(4)	120.6(2)
C(4)-N(5)	1.478(3)	N(4)-N(5)-C(4)	111.29(19)
C(4)-C(5)	1.529(3)	C(4)-C(5)-H(5)	107(2)
C(4)-C(7)	1.529(3)	C(6)-C(5)-C(4)	109.6(2)
C(5)-H(5)	0.98(4)	C(6)-C(5)-H(5)	110(2)
C(5)-C(6)	1.525(4)	C(6)-C(5)-C(9)	109.5(2)
C(5)-C(9)	1.529(4)	C(9)-C(5)-C(4)	111.4(2)
C(6)-H(6A)	0.98(4)	C(9)-C(5)-H(5)	109(2)
C(6)-H(6B)	0.98(6)	H(6A)-C(6)-H(6B)	102(4)
C(6)-H(6C)	0.99(5)	H(6A)-C(6)-H(6C)	117(3)
C(8)-H(8A)	0.97(4)	C(5)-C(6)-H(6A)	111(3)
C(8)-H(8B)	1.00(5)	C(5)-C(6)-H(6B)	112(3)
C(8)-H(8C)	1.04(5)	C(5)-C(6)-H(6C)	109(3)
C(9)-H(9A)	0.91(5)	H(6B)-C(6)-H(6C)	105(4)
C(9)-H(9B)	0.98(4)	O(3)-C(7)-C(4)	110.6(2)
C(9)-H(9C)	0.99(5)	O(4)-C(7)-O(3)	124.4(2)
N(2)-O(1)-N(1)	113.2(2)	O(4)-C(7)-C(4)	124.9(2)
C(1)-N(1)-O(1)	103.4(2)	O(3)-C(8)-H(8A)	108(3)
N(1)-C(1)-C(2)	109.6(2)	O(3)-C(8)-H(8B)	112(3)
N(1)-C(1)-C(3)	132.4(3)	O(3)-C(8)-H(8C)	104(3)
C(2)-C(1)-C(3)	118.0(2)	H(8A)-C(8)-H(8B)	102(3)
C(2)-N(2)-O(1)	103.8(2)	H(8A)-C(8)-H(8C)	116(4)
N(2)-C(2)-C(1)	110.1(3)	H(8B)-C(8)-H(8C)	115(4)
N(2)-C(2)-N(3)	123.5(3)	C(5)-C(9)-H(9A)	120(3)

C(5)-C(9)-H(9B)	110(2)	H(9A)-C(9)-H(9C)	101(4)
C(5)-C(9)-H(9C)	112(3)	H(9B)-C(9)-H(9C)	108(3)
H(9A)-C(9)-H(9B)	105(3)		

Table S10. Anisotropic displacement parameters (Å^{2}x 10^3) for **7h**. The anisotropic displacement factor exponent takes the form: -2p^2[h^2 a*^{2}U^{11} + ... + 2 h k a* b* U^{12}]

	U^{11}	U^{22}	U^{33}	U^{23}	U^{13}	U^{12}
O(1)	17(1)	45(1)	42(1)	-14(1)	-3(1)	13(1)
N(1)	27(1)	32(1)	26(1)	-6(1)	0(1)	10(1)
C(1)	18(1)	23(1)	31(1)	-12(1)	-4(1)	10(1)
O(2)	25(1)	32(1)	31(1)	1(1)	-1(1)	15(1)
N(2)	36(1)	38(1)	37(1)	-13(1)	-8(1)	22(1)
C(2)	30(1)	22(1)	29(1)	-9(1)	2(1)	12(1)
O(3)	24(1)	19(1)	28(1)	-1(1)	4(1)	7(1)
N(3)	30(1)	31(1)	37(1)	2(1)	-7(1)	18(1)
C(3)	24(1)	21(1)	24(1)	-1(1)	3(1)	9(1)
O(4)	30(1)	24(1)	36(1)	3(1)	13(1)	8(1)
N(4)	22(1)	26(1)	38(1)	-5(1)	-4(1)	15(1)
C(4)	17(1)	21(1)	20(1)	3(1)	2(1)	10(1)
N(5)	16(1)	21(1)	26(1)	-2(1)	2(1)	9(1)
C(5)	24(1)	24(1)	23(1)	2(1)	3(1)	14(1)
C(6)	27(1)	38(2)	35(2)	2(1)	6(1)	22(1)
C(7)	16(1)	21(1)	24(1)	2(1)	1(1)	10(1)
C(8)	24(1)	21(1)	39(2)	-5(1)	0(1)	9(1)
C(9)	33(2)	24(1)	36(2)	-5(1)	6(1)	13(1)

Table S11. Hydrogen coordinates (x 10^4) and isotropic displacement parameters ($Å^2$x 10^3) for **7h**.

	x	y	z	U(eq)
H(6A)	12470(50)	7160(50)	3490(50)	46(11)
H(4)	10220(30)	6560(30)	5510(30)	19(7)
H(8A)	11510(50)	11210(50)	5190(50)	48(11)
H(5)	10170(40)	6670(40)	2470(40)	27(8)
H(9A)	8690(50)	3910(50)	4030(50)	53(12)
H(6B)	11850(60)	5750(60)	2580(60)	75(15)
H(6C)	11580(50)	5490(50)	4280(50)	54(12)
H(8B)	12780(50)	11100(50)	5900(50)	52(12)
H(8C)	11410(60)	10980(60)	7050(50)	66(14)
H(9B)	9110(40)	4010(40)	2440(40)	31(8)
H(9C)	7960(50)	4460(50)	3050(40)	49(11)

Table S12. Torsion angles [°] for **7h**.

O(1)-N(1)-C(1)-C(2)	0.3(3)	N(3)-N(4)-N(5)-C(3)	-1.9(4)
O(1)-N(1)-C(1)-C(3)	-177.6(3)	N(3)-N(4)-N(5)-C(4)	172.7(2)
O(1)-N(2)-C(2)-C(1)	0.5(3)	C(3)-C(1)-C(2)-N(2)	177.7(2)
O(1)-N(2)-C(2)-N(3)	179.1(2)	C(3)-C(1)-C(2)-N(3)	-0.8(4)
N(1)-O(1)-N(2)-C(2)	-0.3(3)	N(5)-C(4)-C(5)-C(6)	-165.9(2)
N(1)-C(1)-C(2)-N(2)	-0.5(3)	N(5)-C(4)-C(5)-C(9)	-44.6(3)
N(1)-C(1)-C(2)-N(3)	-179.1(3)	N(5)-C(4)-C(7)-O(3)	57.4(2)
N(1)-C(1)-C(3)-O(2)	-1.8(5)	N(5)-C(4)-C(7)-O(4)	-124.6(3)
N(1)-C(1)-C(3)-N(5)	176.3(3)	C(5)-C(4)-N(5)-C(3)	103.6(3)
C(1)-C(2)-N(3)-N(4)	2.0(4)	C(5)-C(4)-N(5)-N(4)	-71.5(2)
C(1)-C(3)-N(5)-N(4)	2.9(3)	C(5)-C(4)-C(7)-O(3)	-178.3(2)
C(1)-C(3)-N(5)-C(4)	-171.3(2)	C(5)-C(4)-C(7)-O(4)	-0.3(3)
O(2)-C(3)-N(5)-N(4)	-178.9(2)	C(7)-C(4)-N(5)-C(3)	-130.6(2)
O(2)-C(3)-N(5)-C(4)	6.9(4)	C(7)-C(4)-N(5)-N(4)	54.3(2)
N(2)-O(1)-N(1)-C(1)	0.0(3)	C(7)-C(4)-C(5)-C(6)	71.5(3)
N(2)-C(2)-N(3)-N(4)	-176.4(3)	C(7)-C(4)-C(5)-C(9)	-167.2(2)
C(2)-C(1)-C(3)-O(2)	-179.5(3)	C(8)-O(3)-C(7)-O(4)	1.0(4)
C(2)-C(1)-C(3)-N(5)	-1.4(3)	C(8)-O(3)-C(7)-C(4)	179.0(2)
C(2)-N(3)-N(4)-N(5)	-0.7(4)		

Table S13. Hydrogen bonds for **7h** [Å and °].

D-H...A	d(D-H)	d(H...A)	d(D...A)	<(DHA)
C(8)-H(8A)...O(3)#1	0.97(4)	2.60(4)	3.537(4)	161(3)

Symmetry transformations used to generate equivalent atoms:

#1 -y+2,x-y+1,z-1/3

10. DSC data

DSC data for compound **1a**

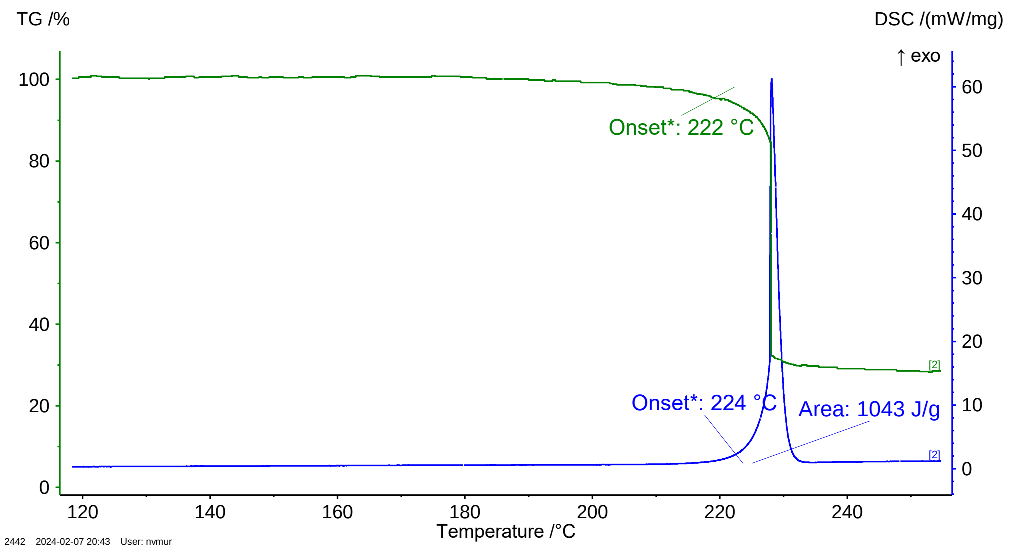

DSC data for compound **1b**

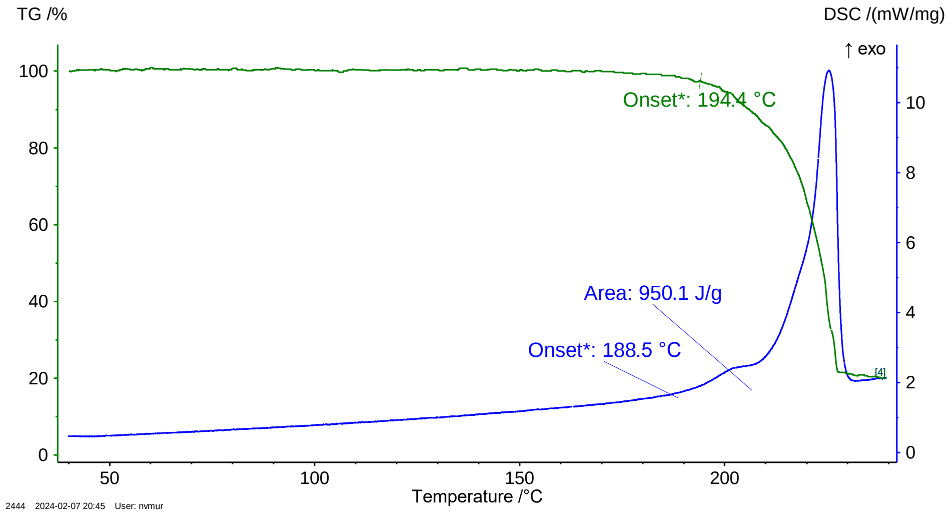

DSC data for compound **1c**

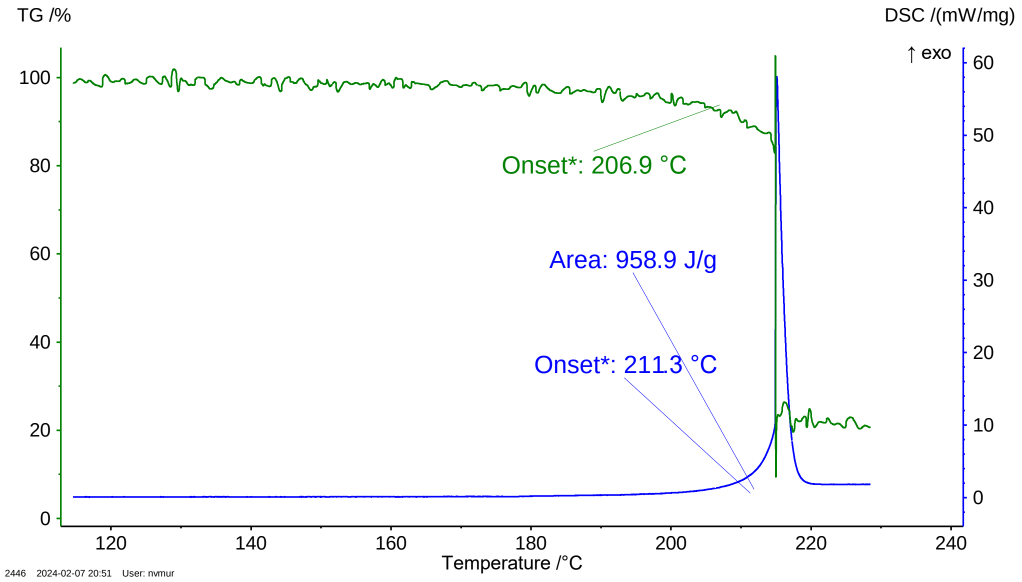

DSC data for compound **1d**

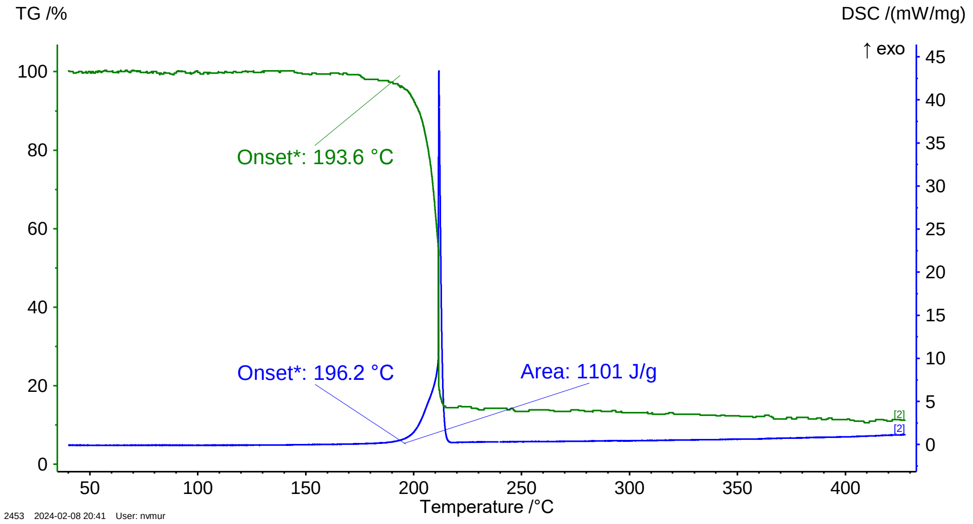

DSC data for compound **1e**

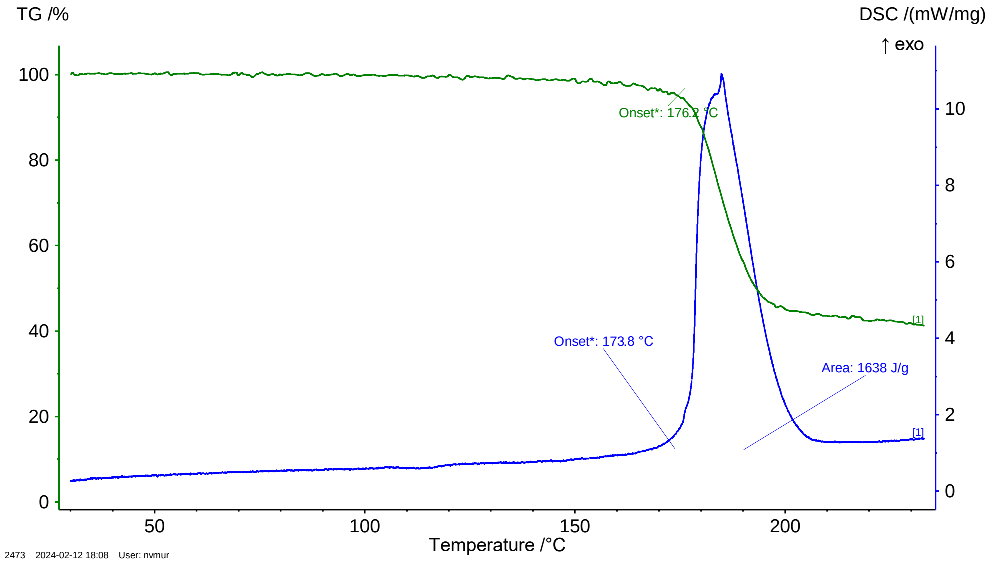

DSC data for compound **1f**

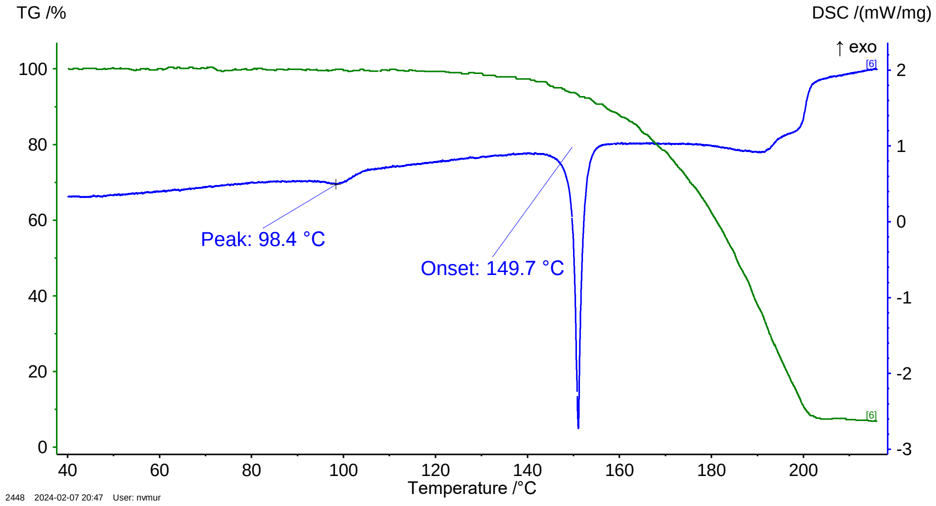

DSC data for compound **1g**

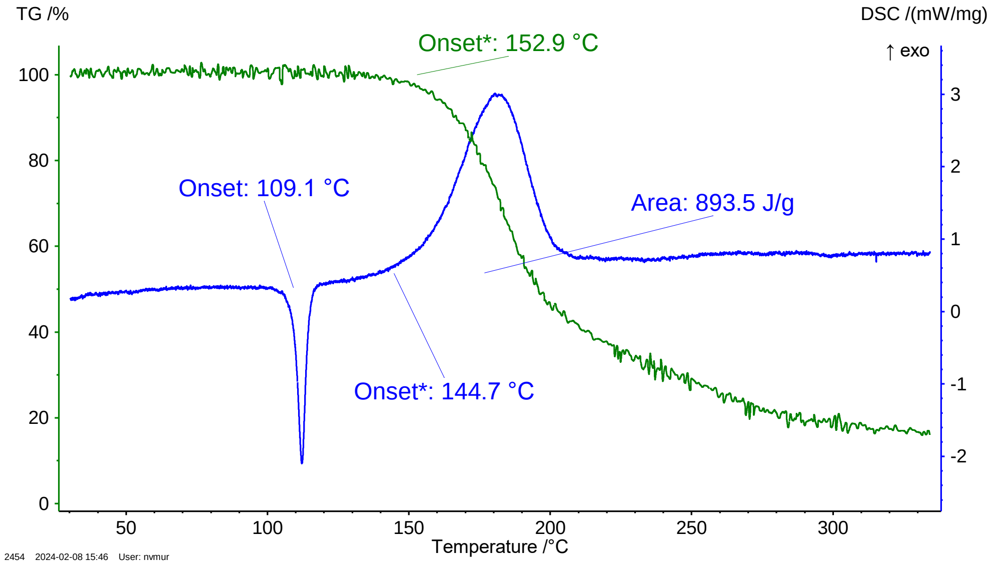

DSC data for compound **1h**

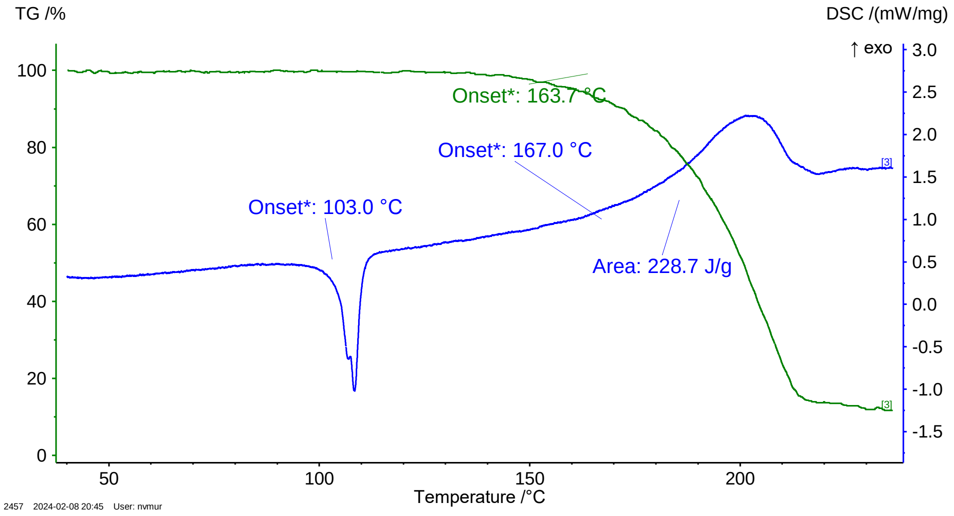

DSC data for compound **7a**

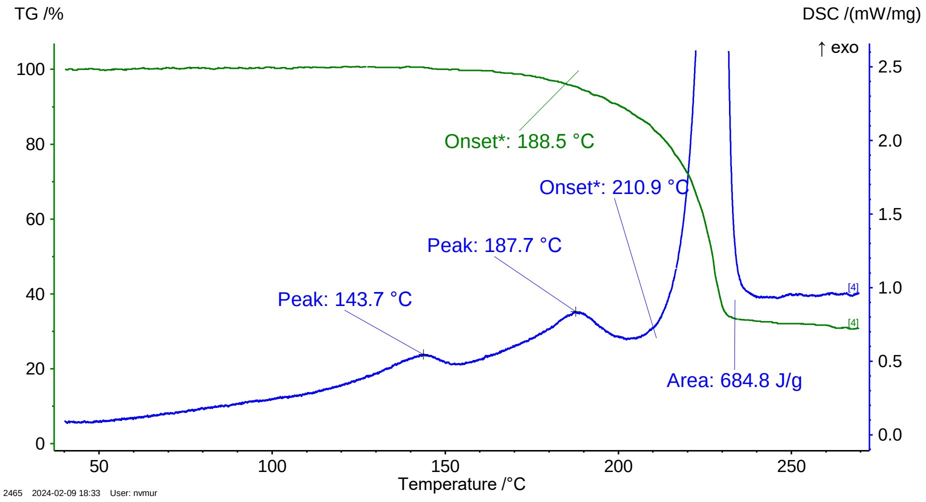

DSC data for compound **7b**

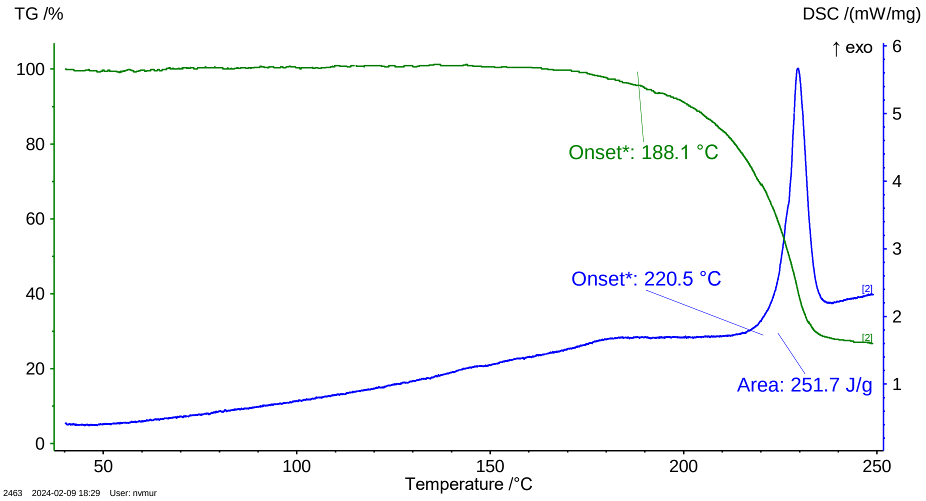

DSC data for compound **7c**

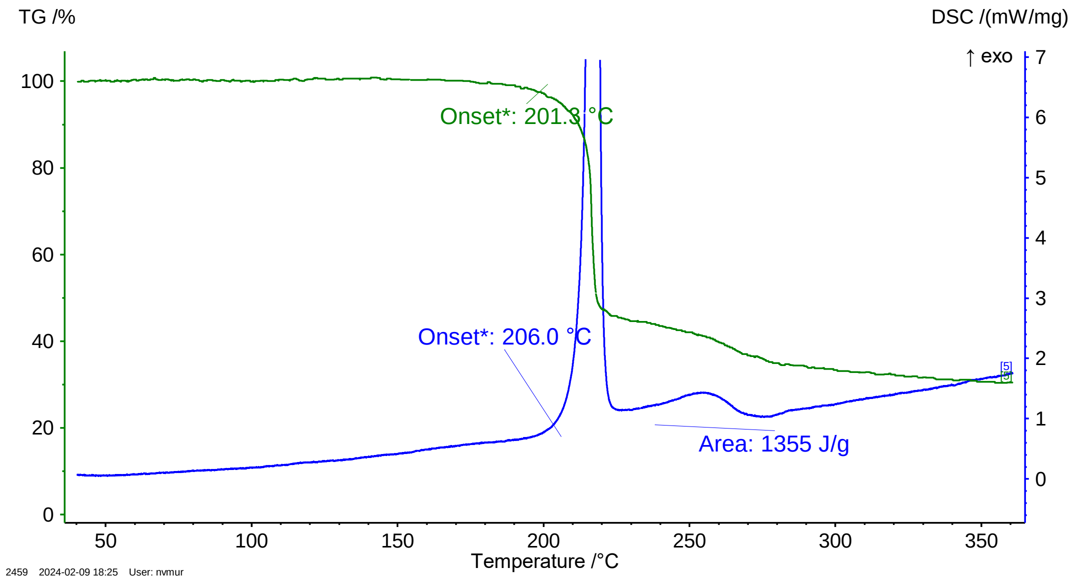

DSC data for compound **7d**

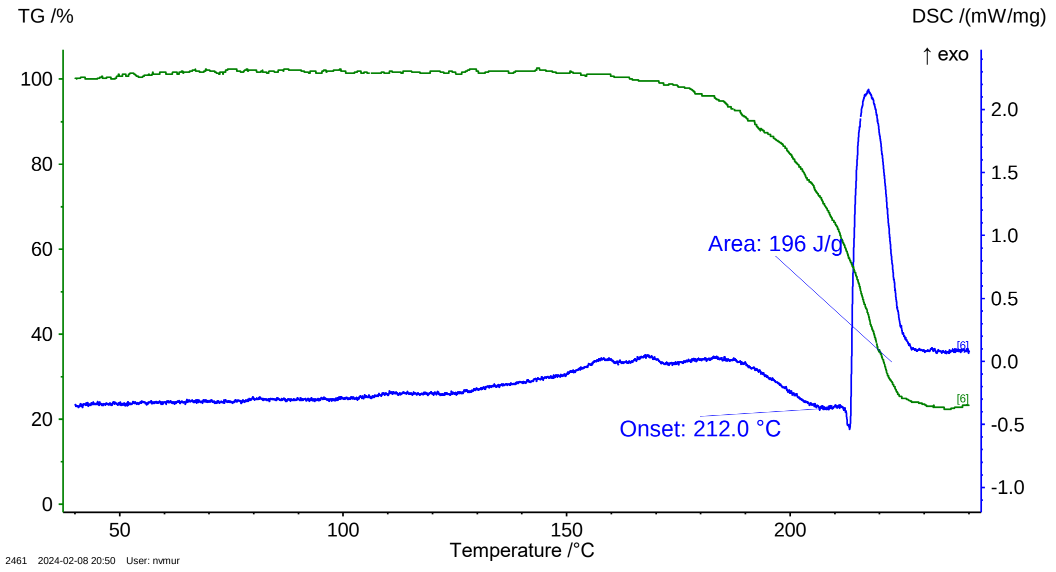

DSC data for compound **7e**

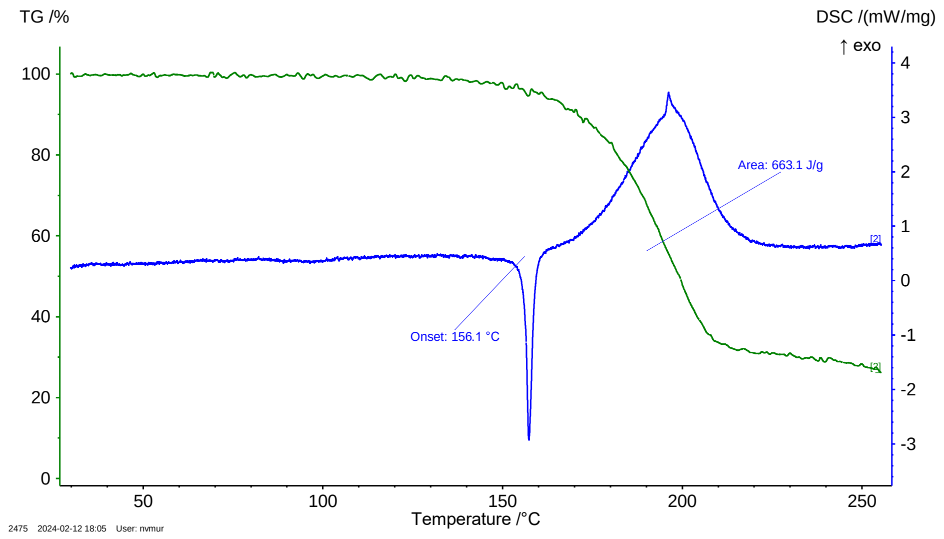

DSC data for compound **7f**

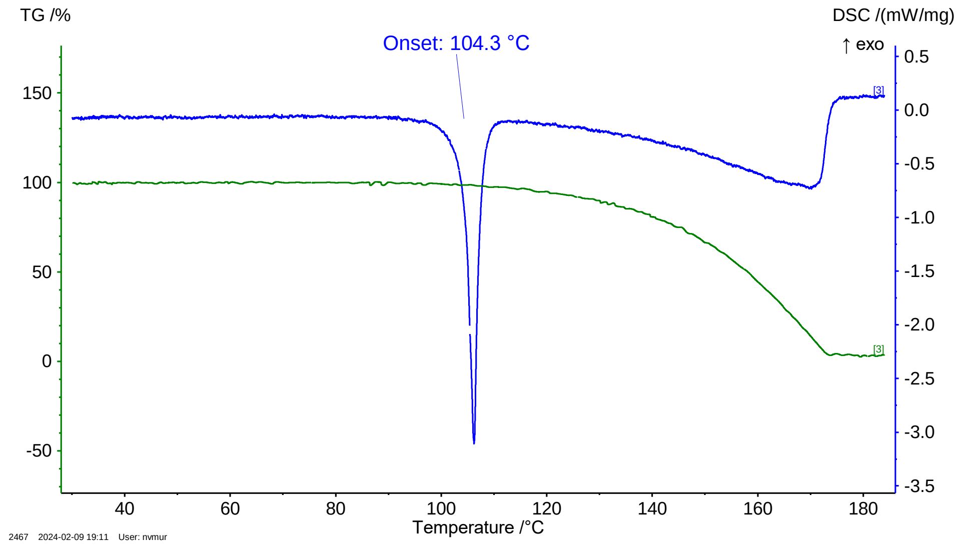

DSC data for compound **7g**

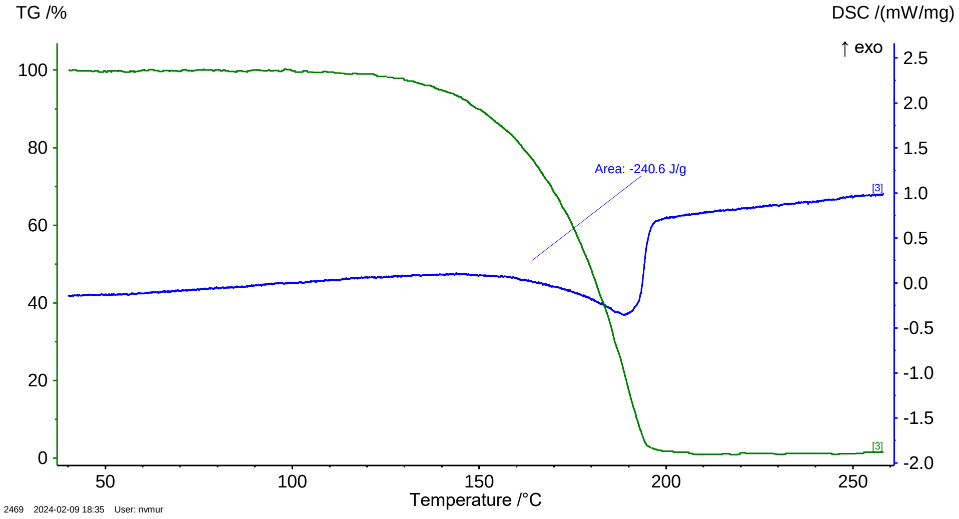

DSC data for compound **7h**

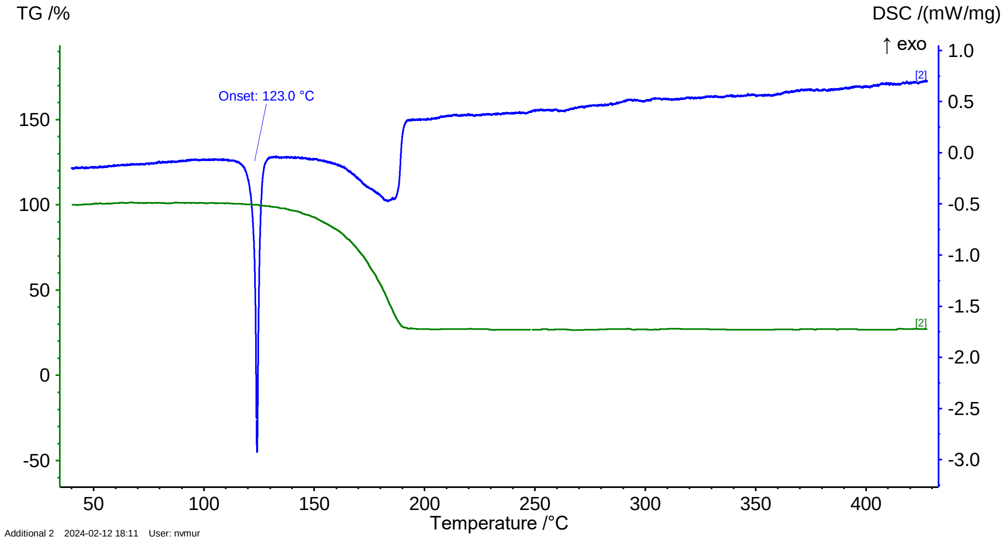

11. Copies of IR spectra

IR spectrum for compound **2a**

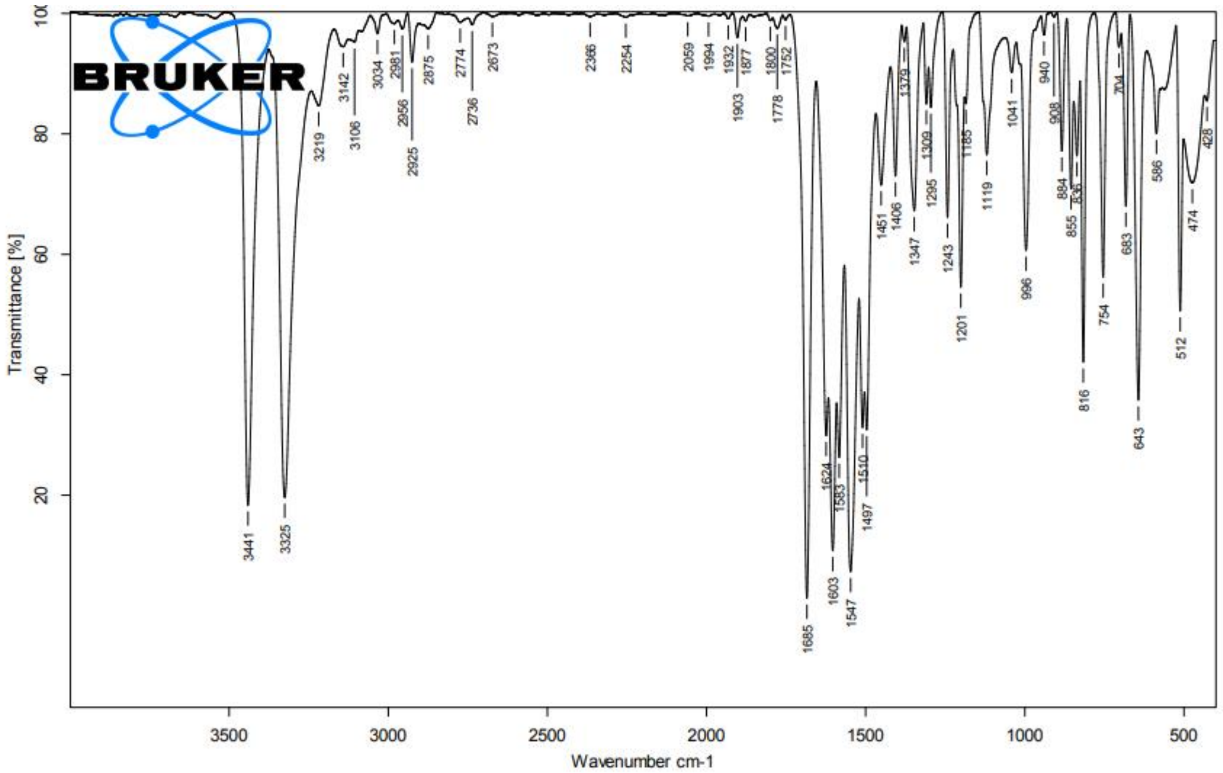

IR spectrum for compound **2b**

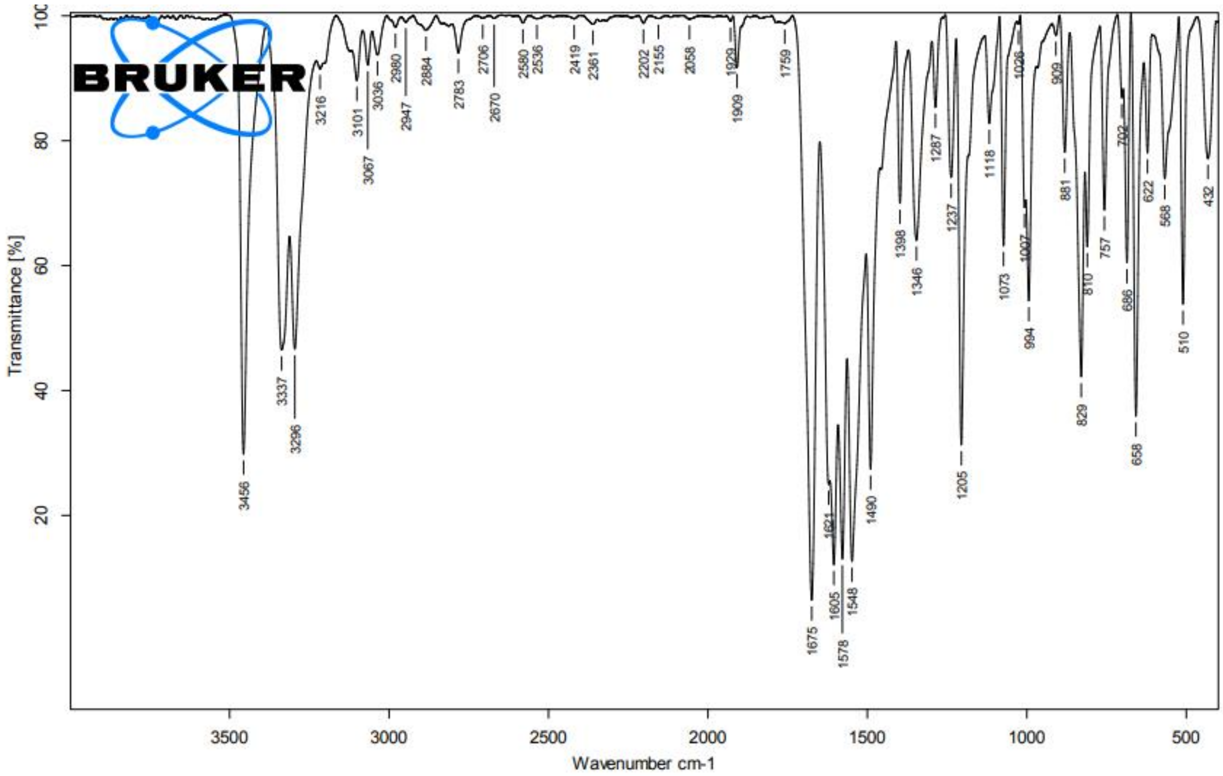

IR spectrum for compound **2c**

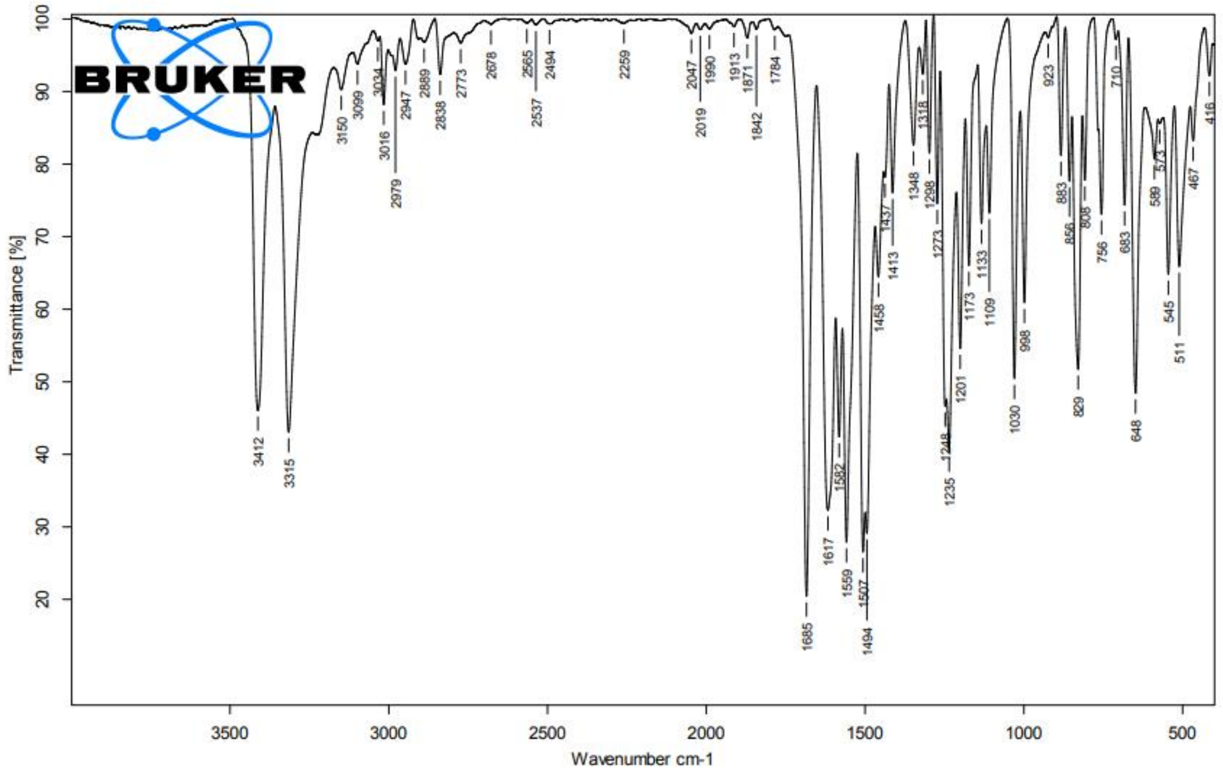

IR spectrum for compound **2d**

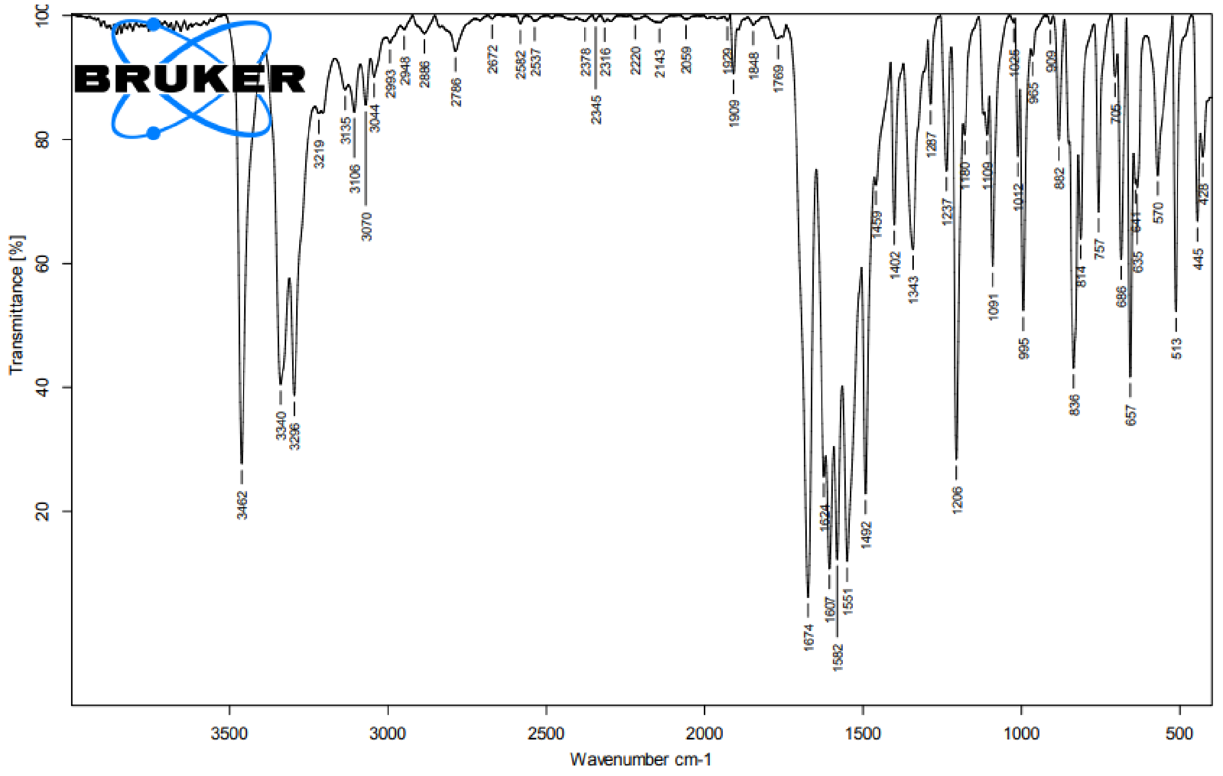

IR spectrum for compound **2e**

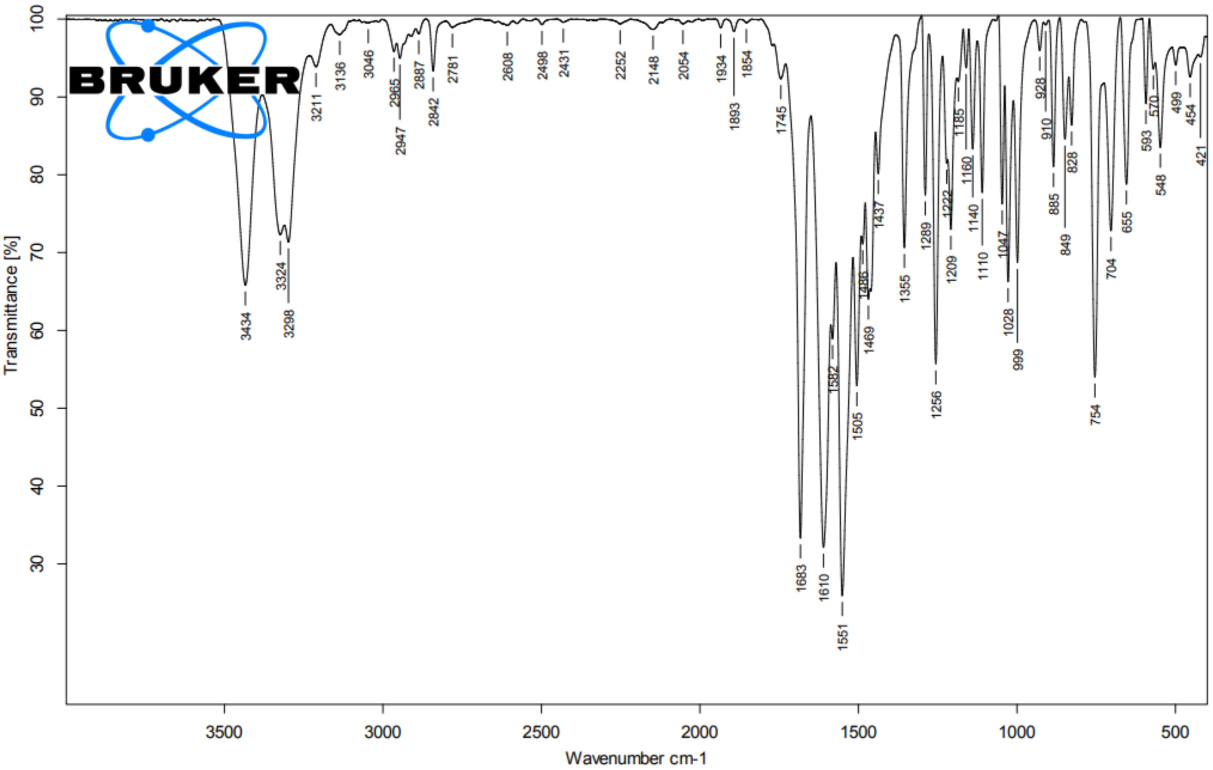

IR spectrum for compound **2f**

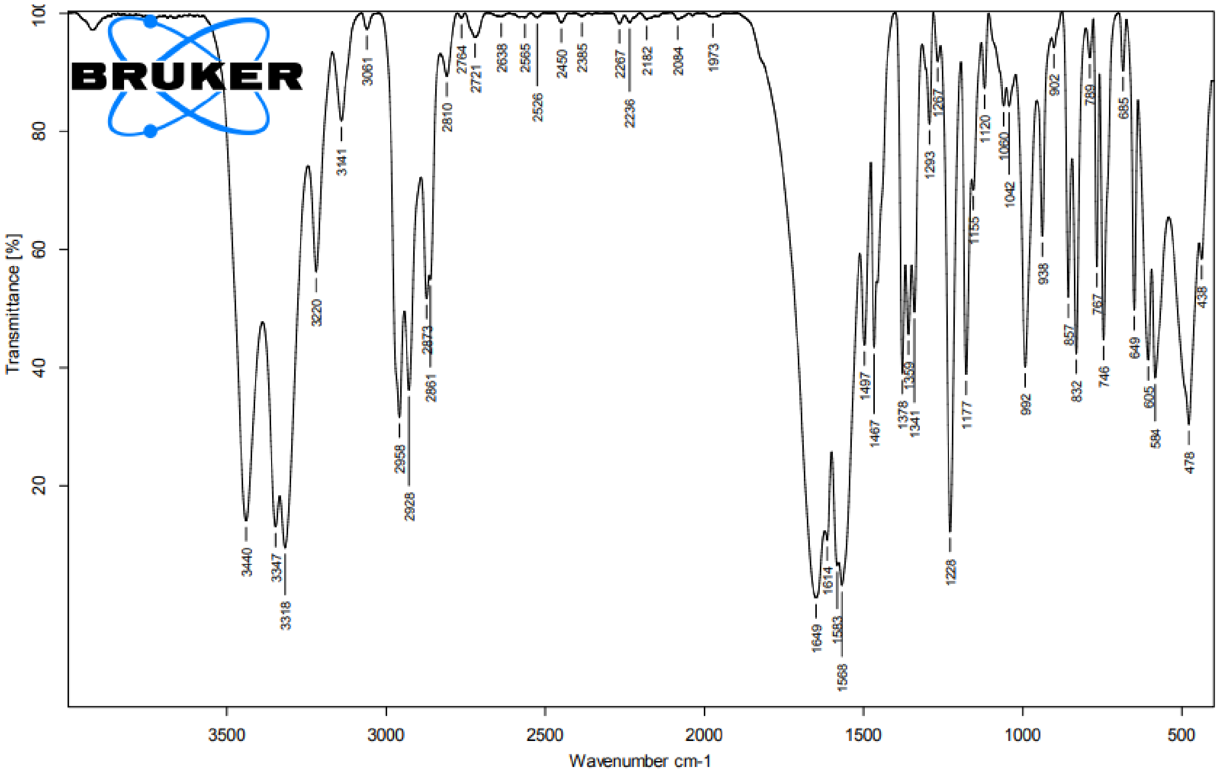

IR spectrum for compound **2g**

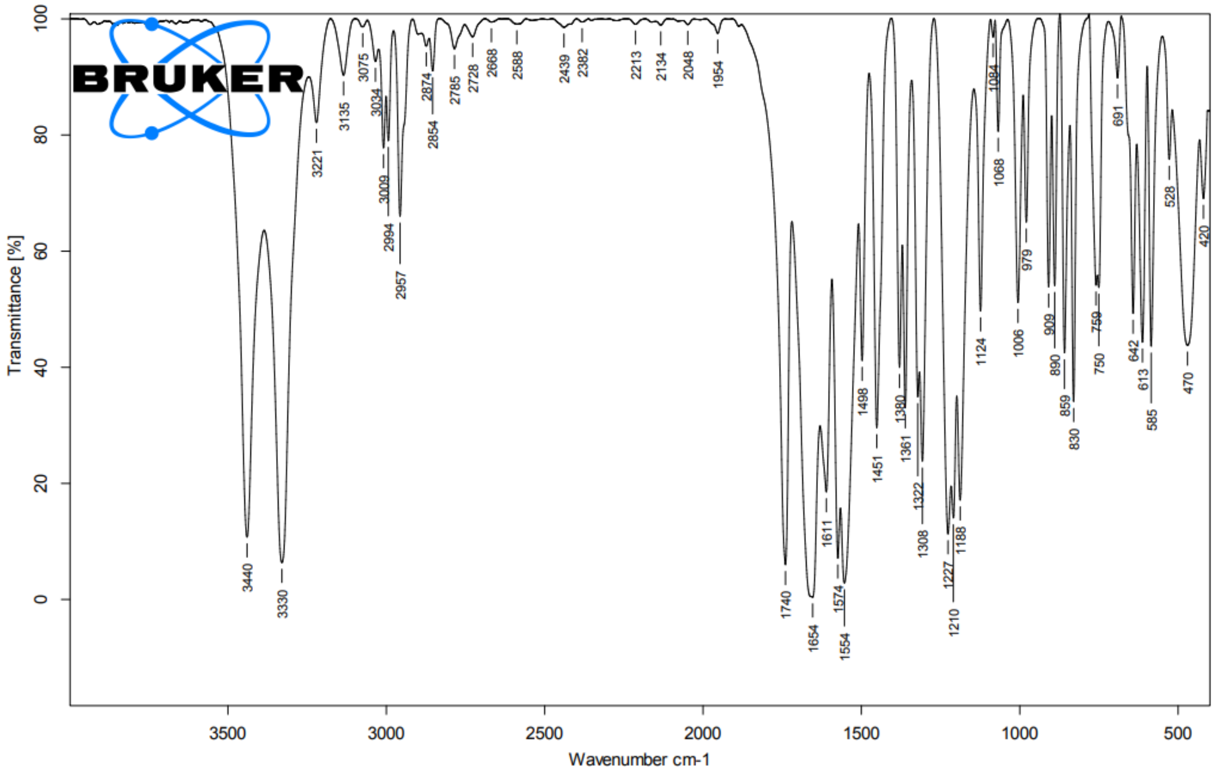

IR spectrum for compound **2h**

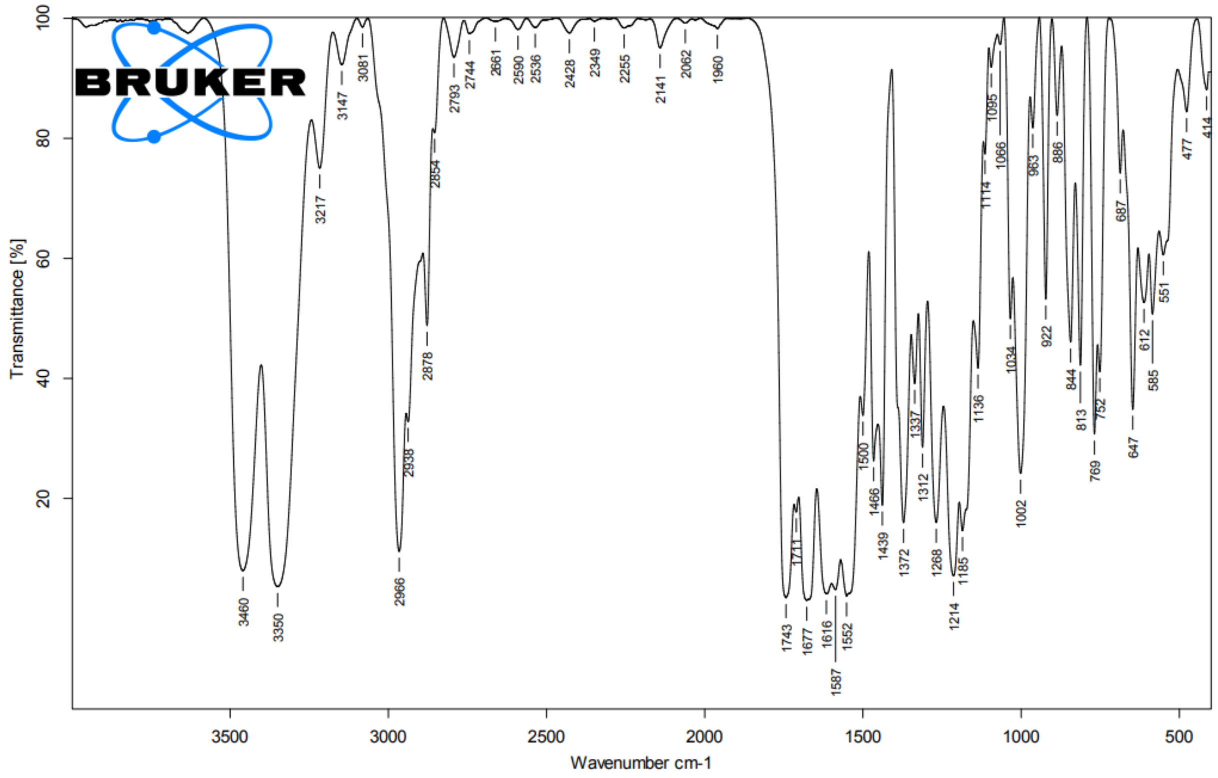

IR spectrum for compound **5a**

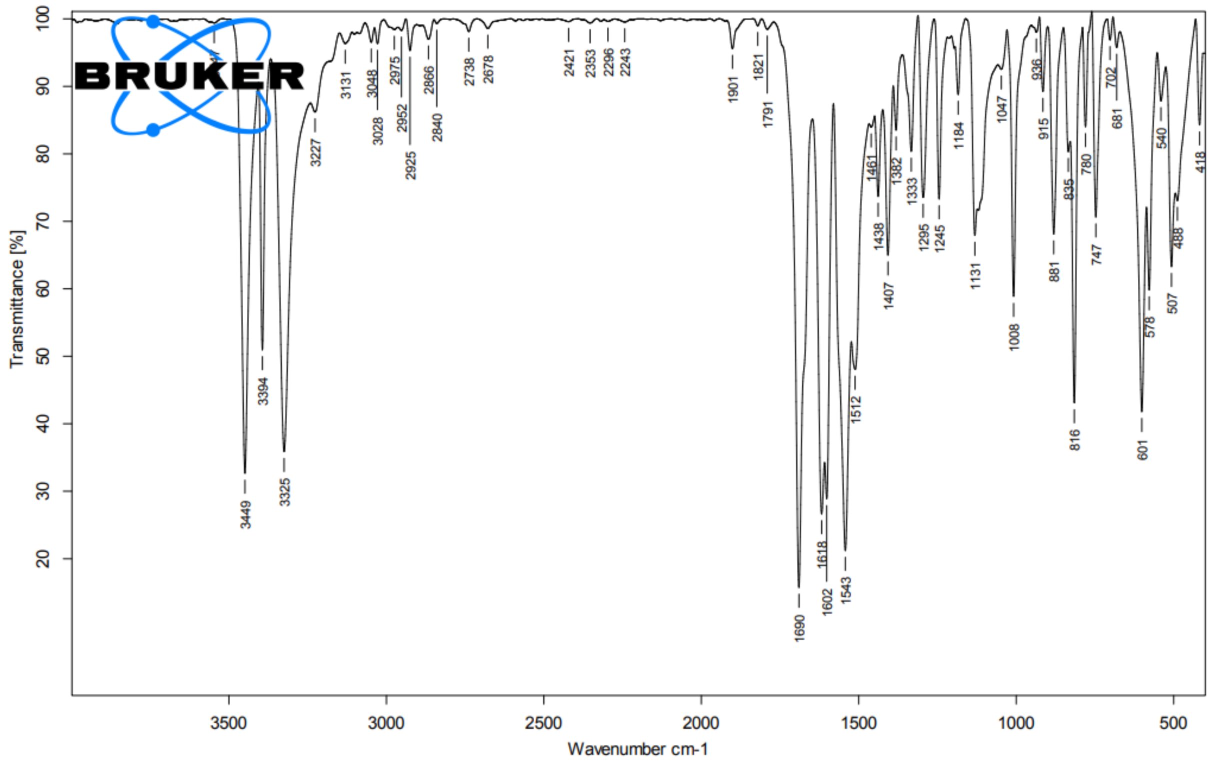

IR spectrum for compound **5b**

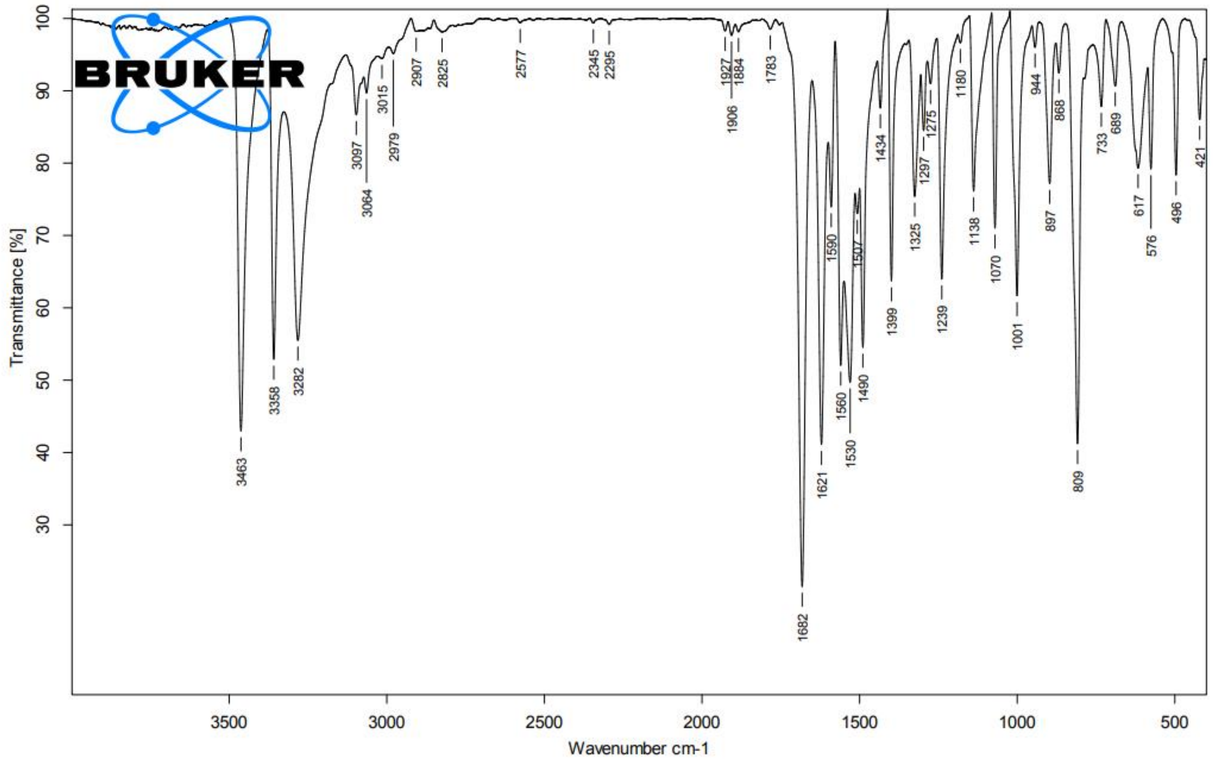

IR spectrum for compound **5c**

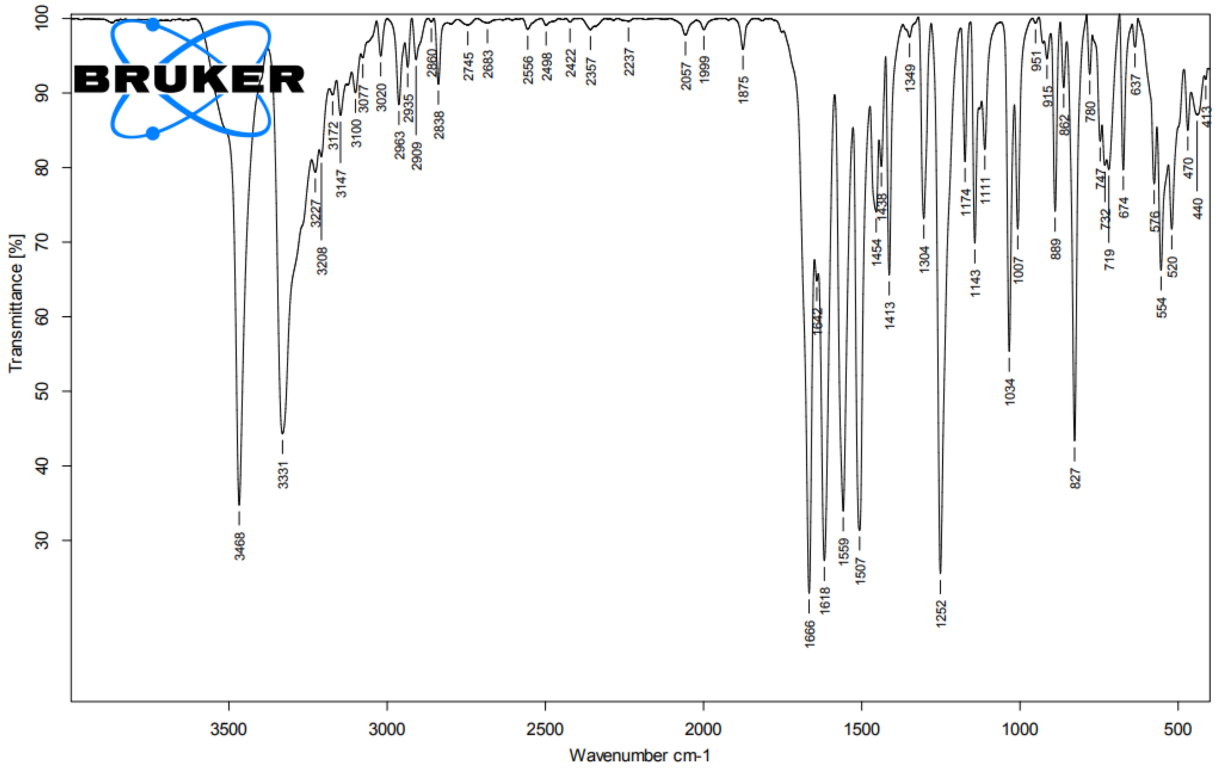

IR spectrum for compound **5d**

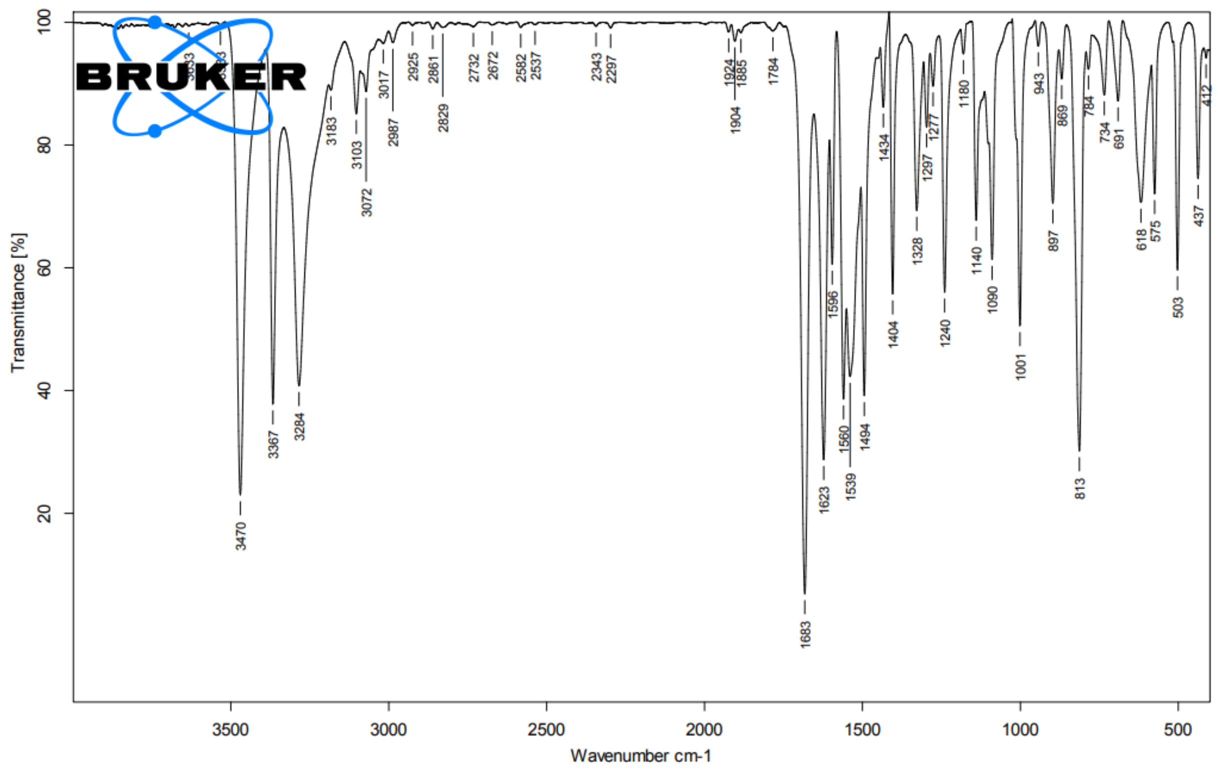

IR spectrum for compound **5e**

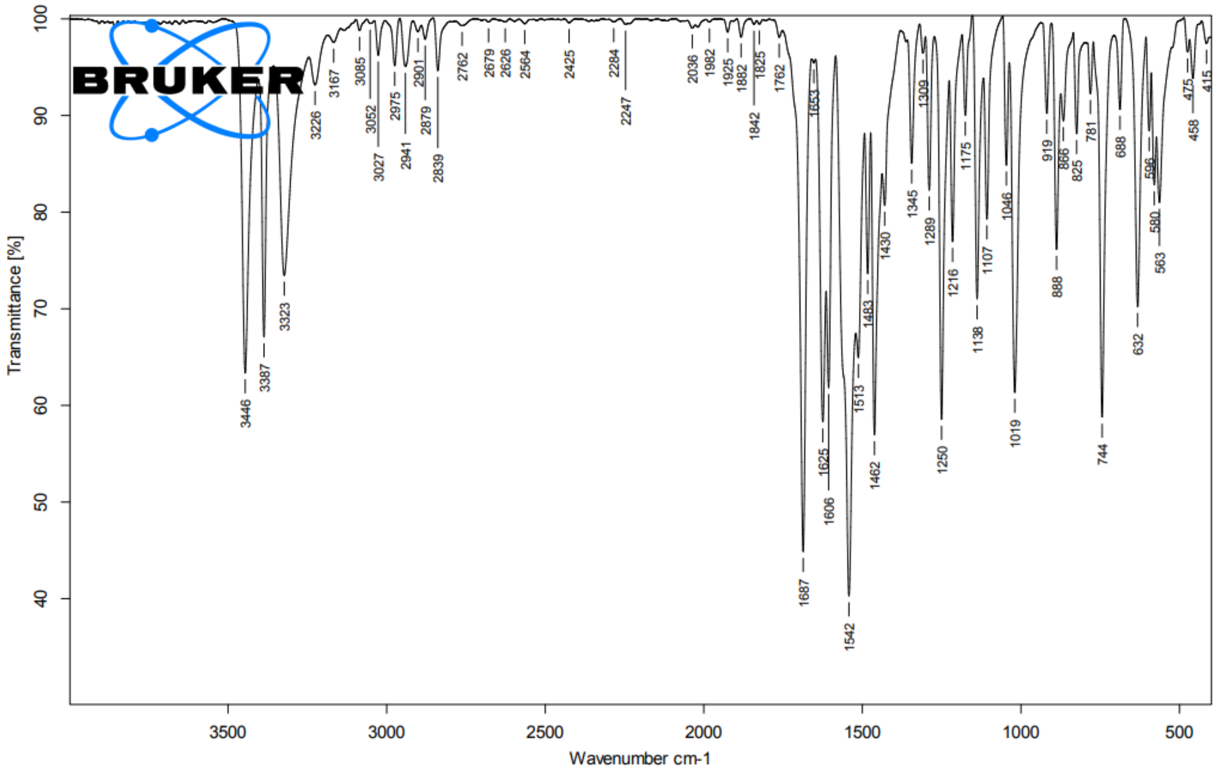

IR spectrum for compound **5f**

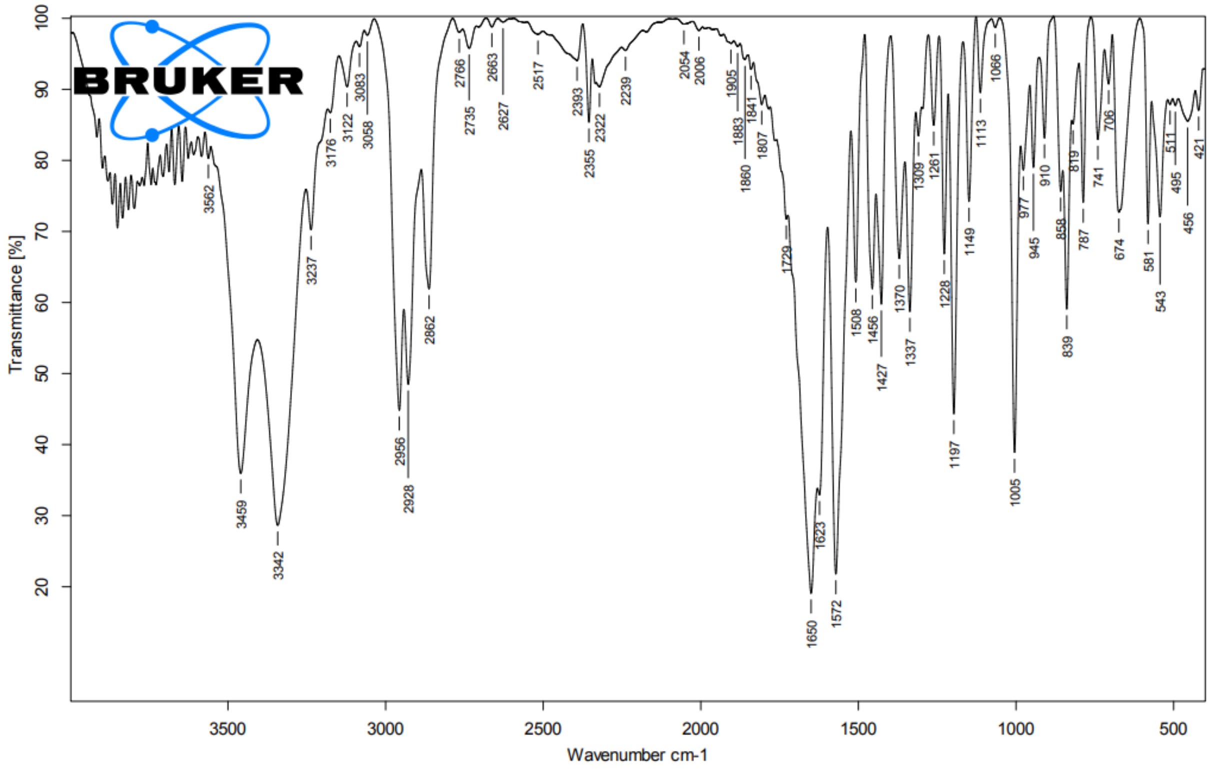

IR spectrum for compound **5g**

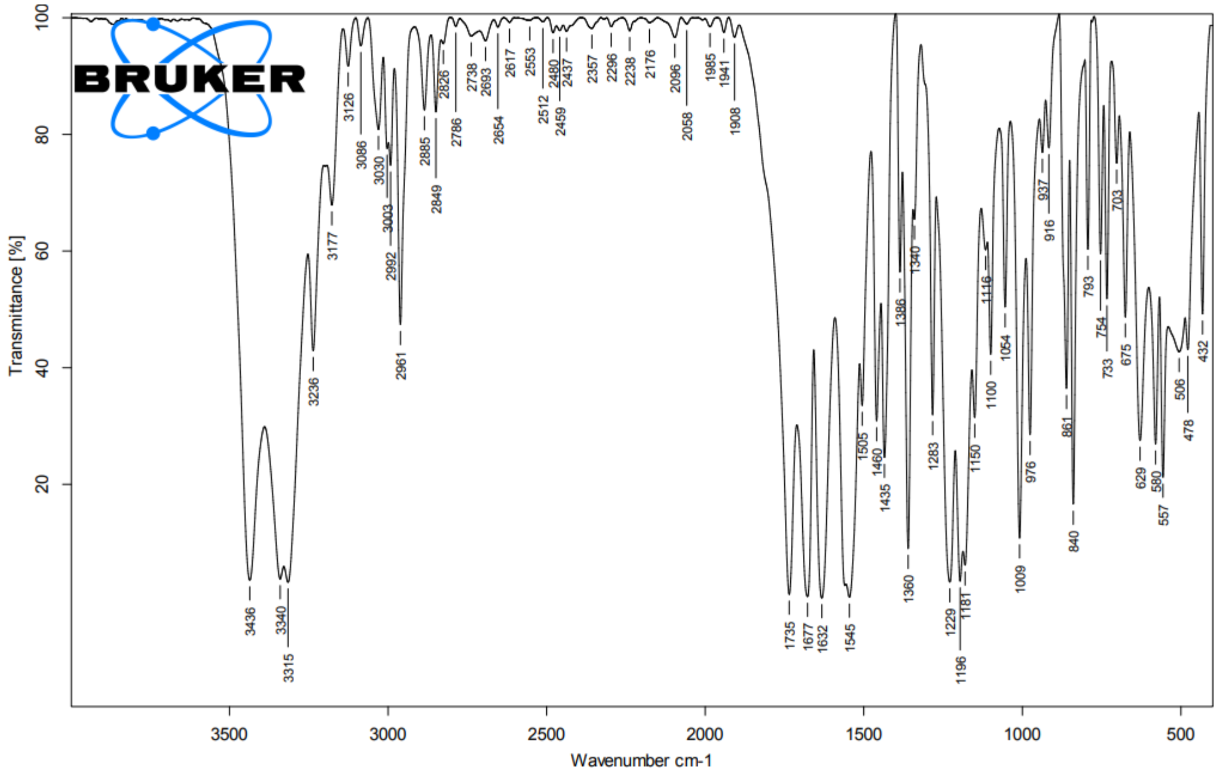

IR spectrum for compound **5h**

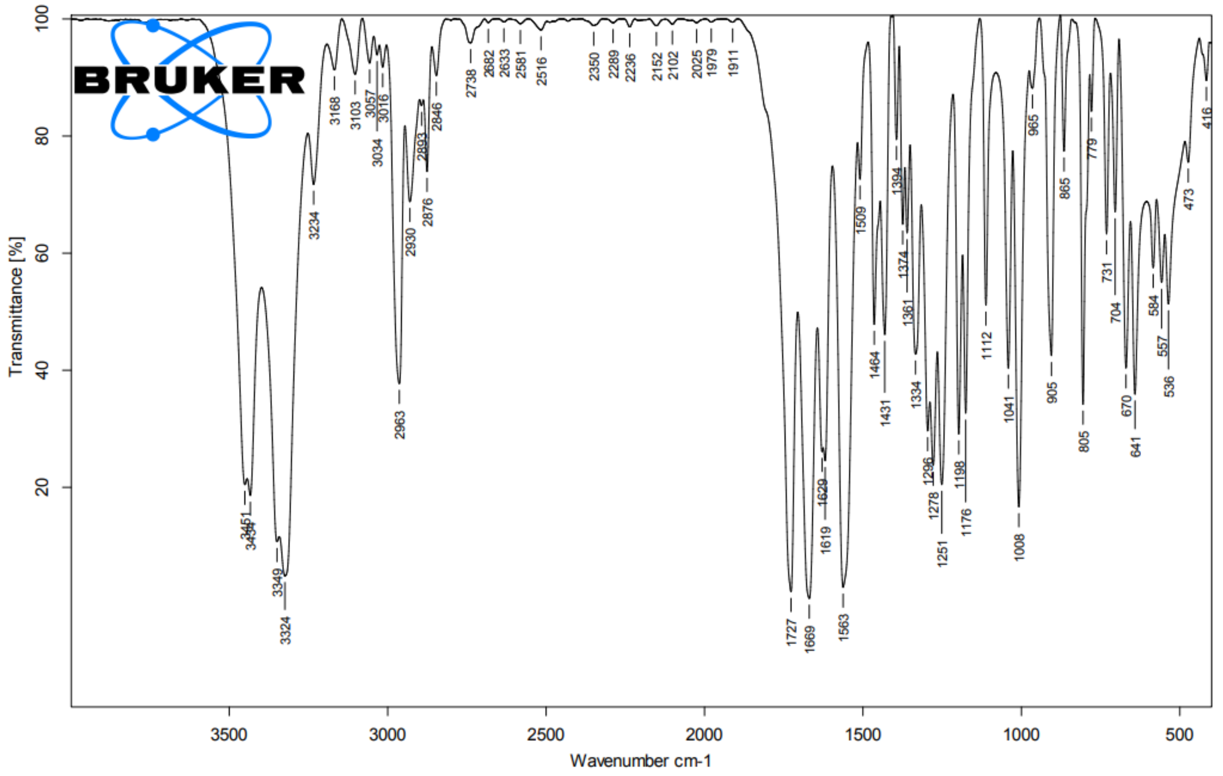

IR spectrum for compound **1a**

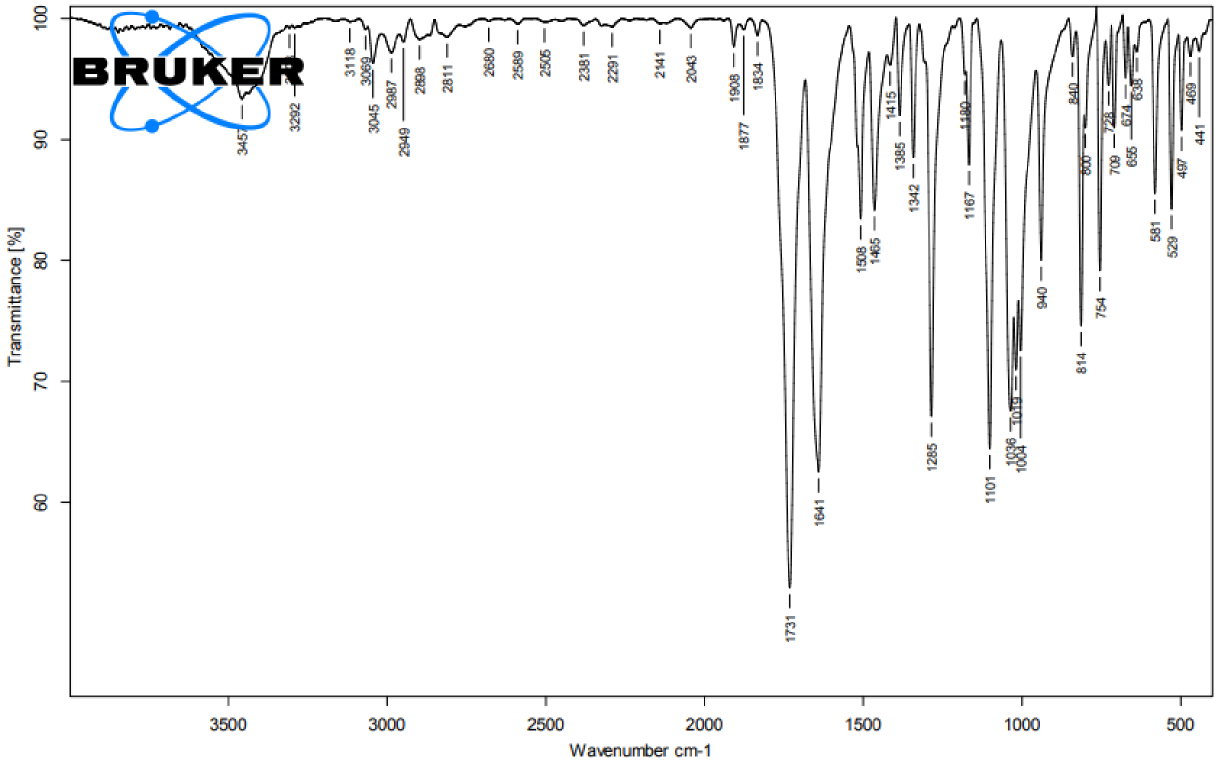

IR spectrum for compound **1b**

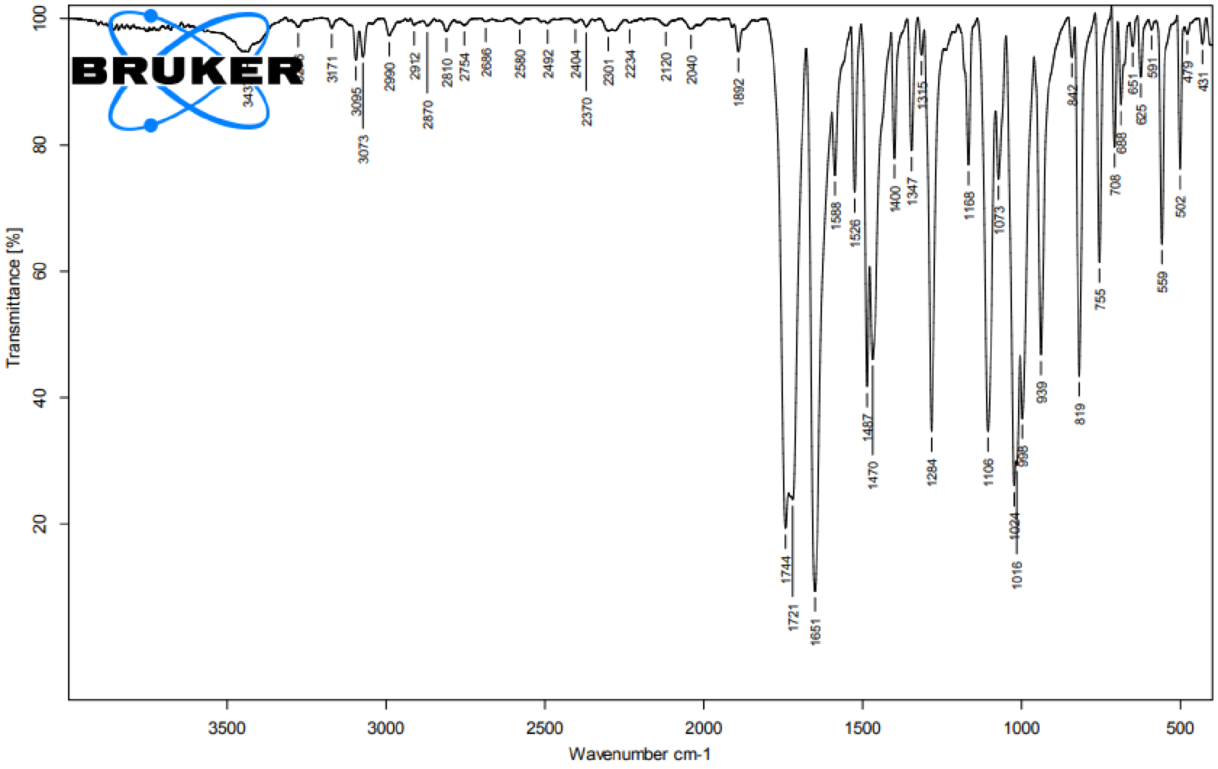

IR spectrum for compound **1c**

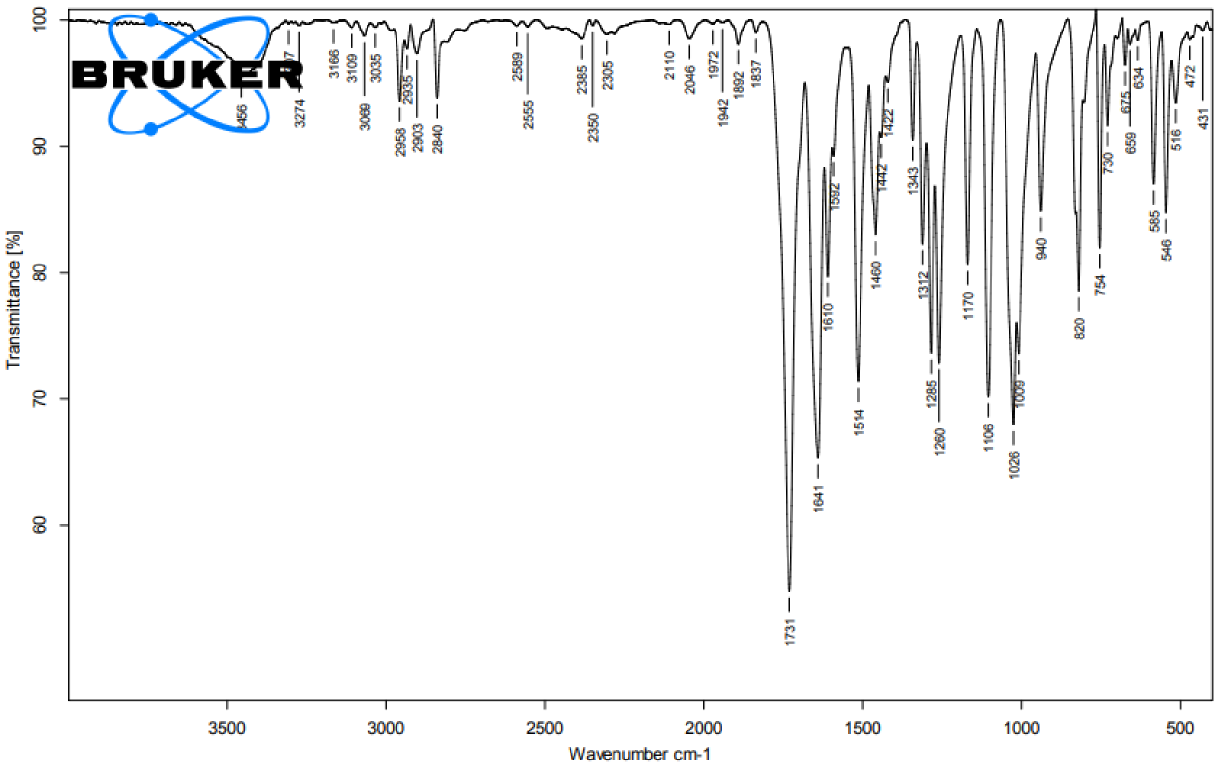

IR spectrum for compound **1d**

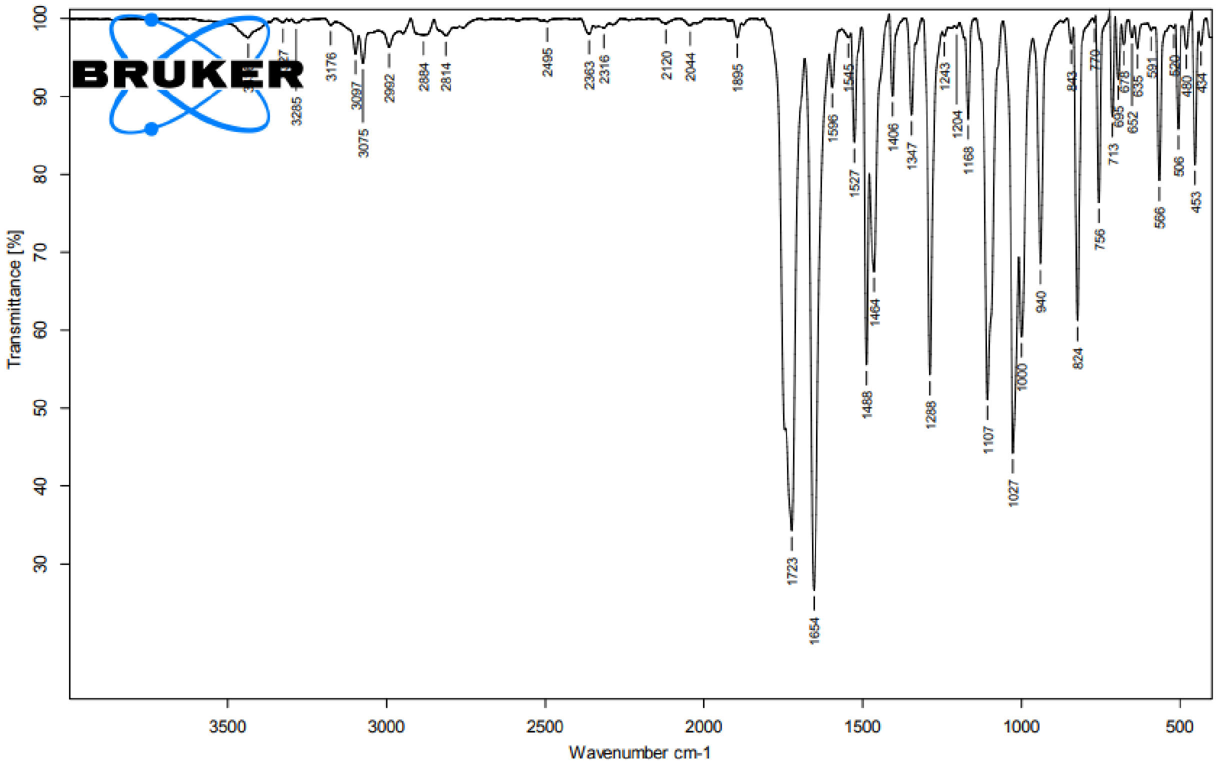

IR spectrum for compound **1e**

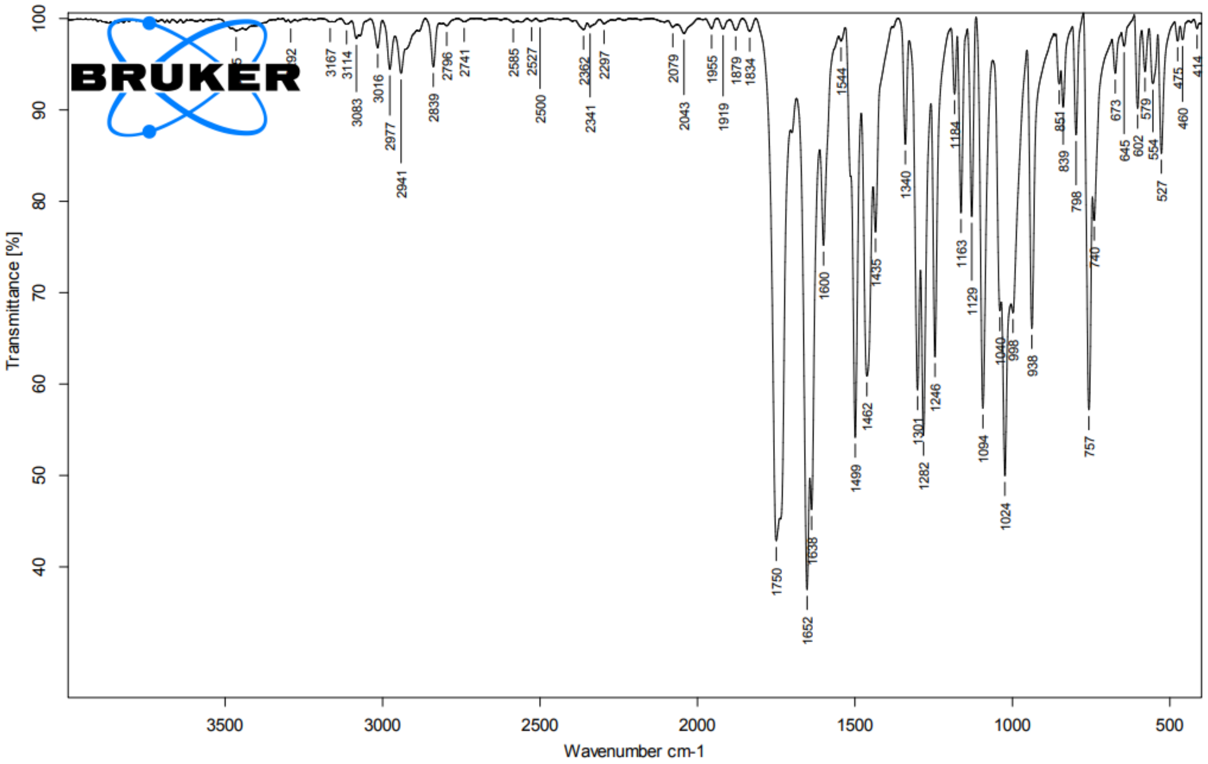

IR spectrum for compound **1f**

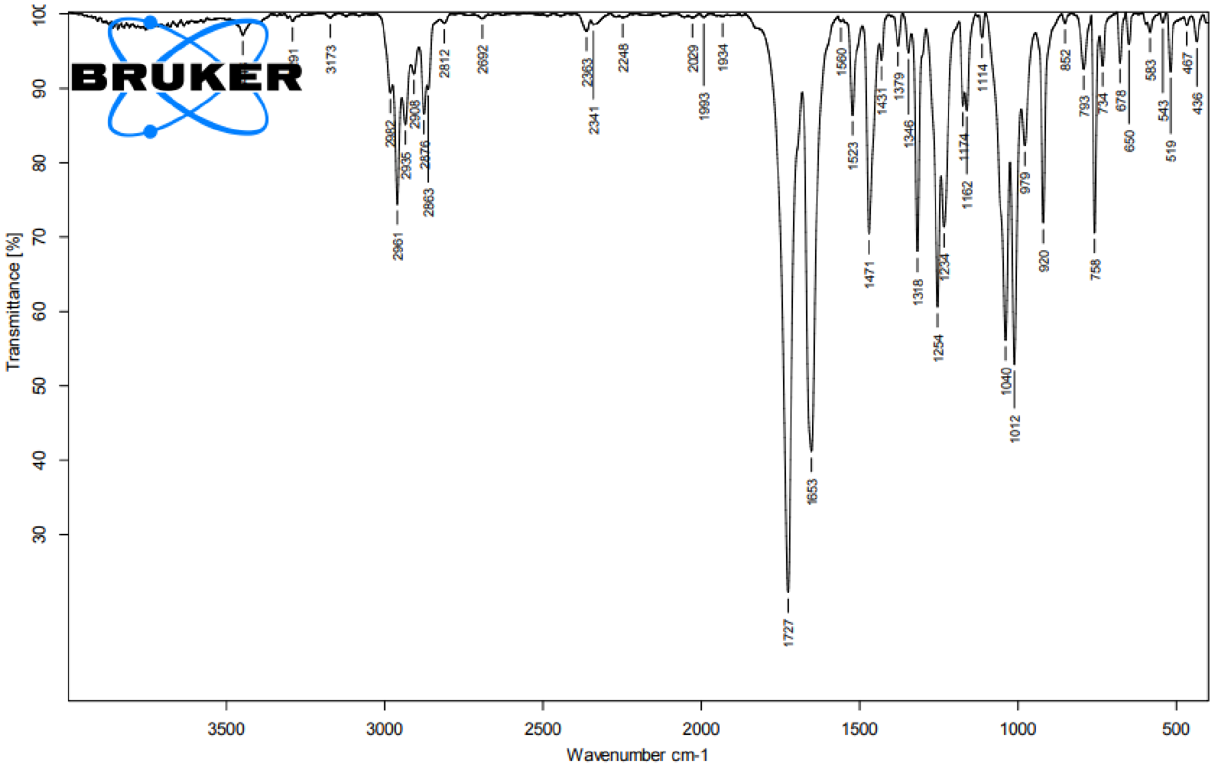

IR spectrum for compound **1g**

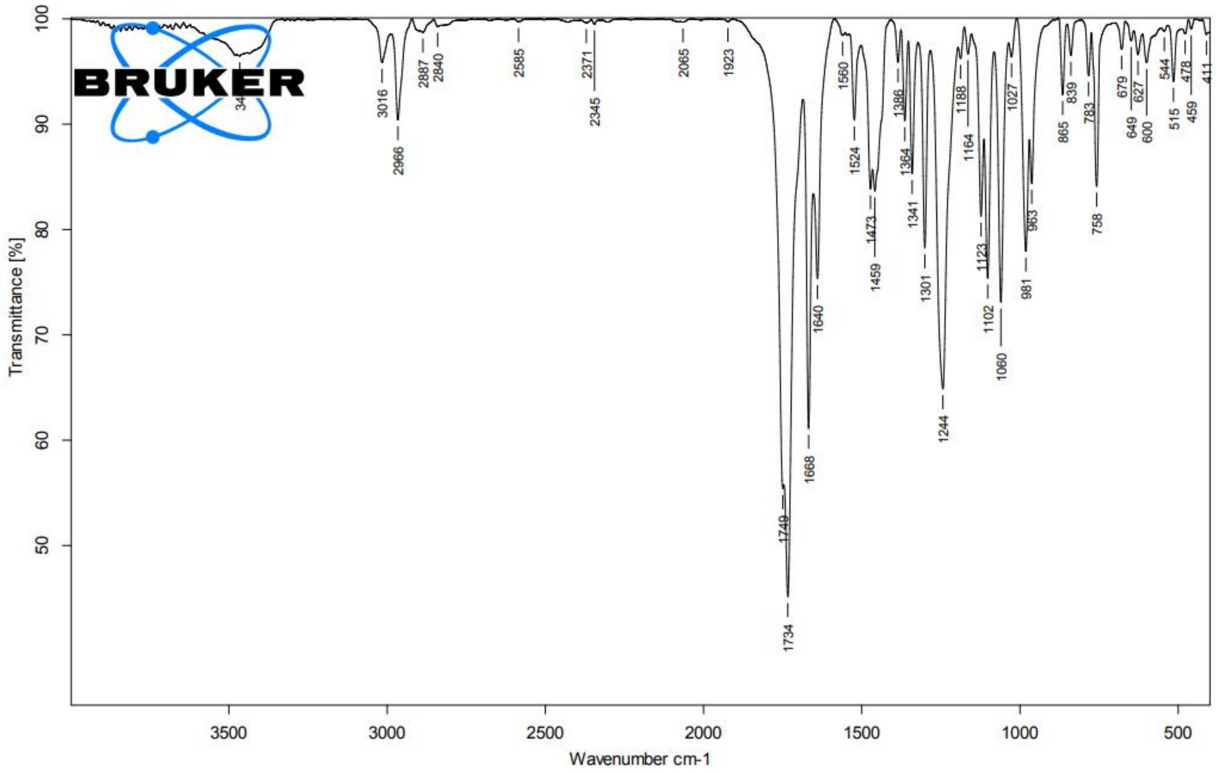

IR spectrum for compound **1h**

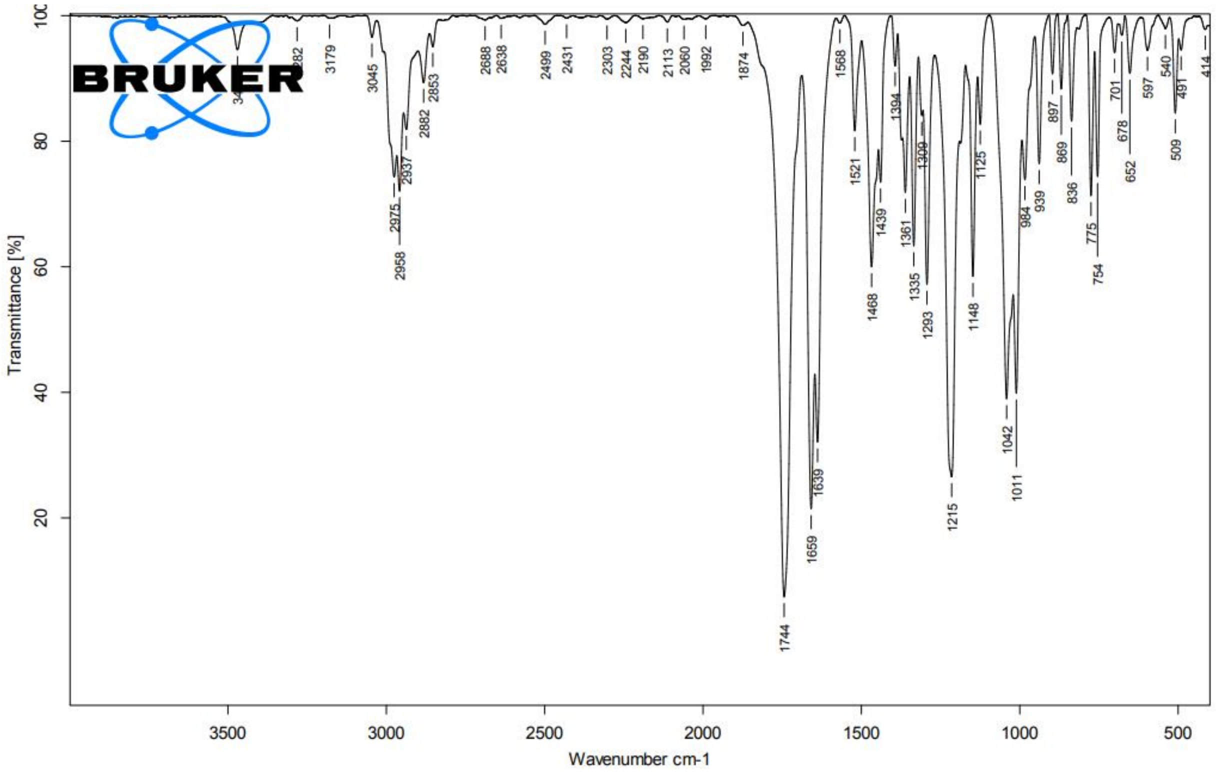

IR spectrum for compound **7a**

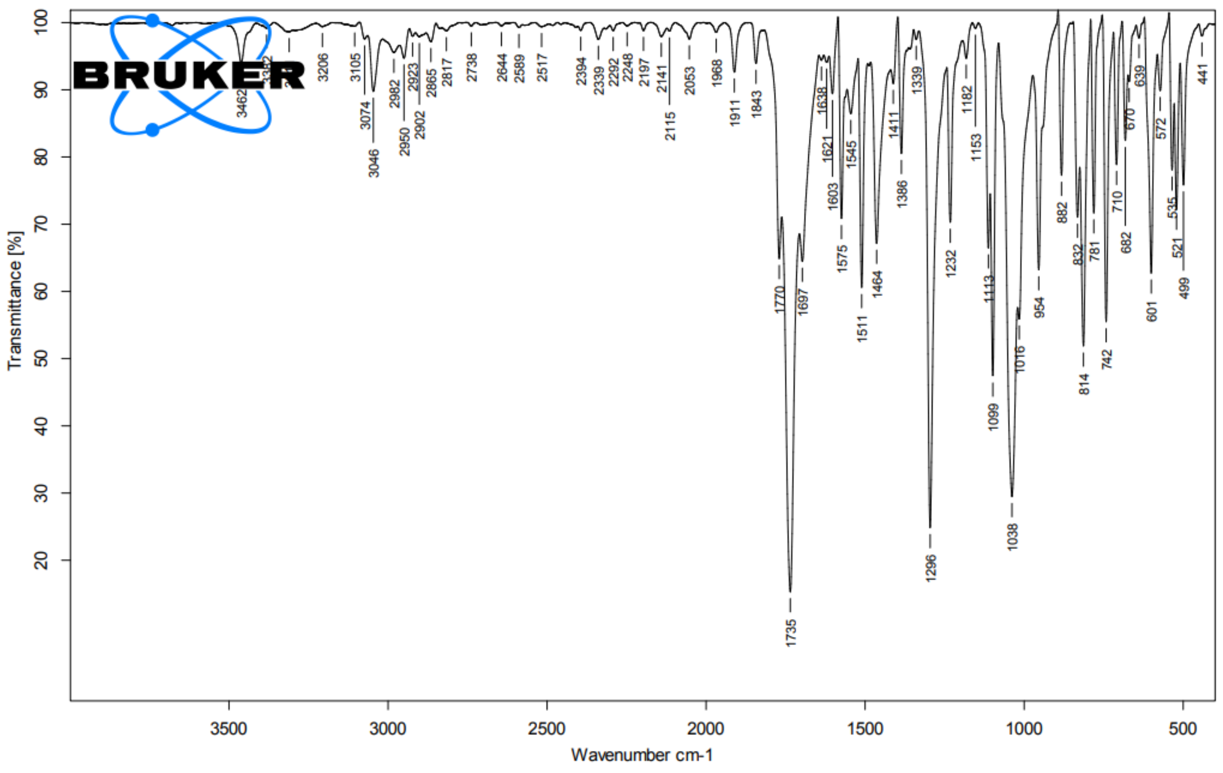

IR spectrum for compound **7b**

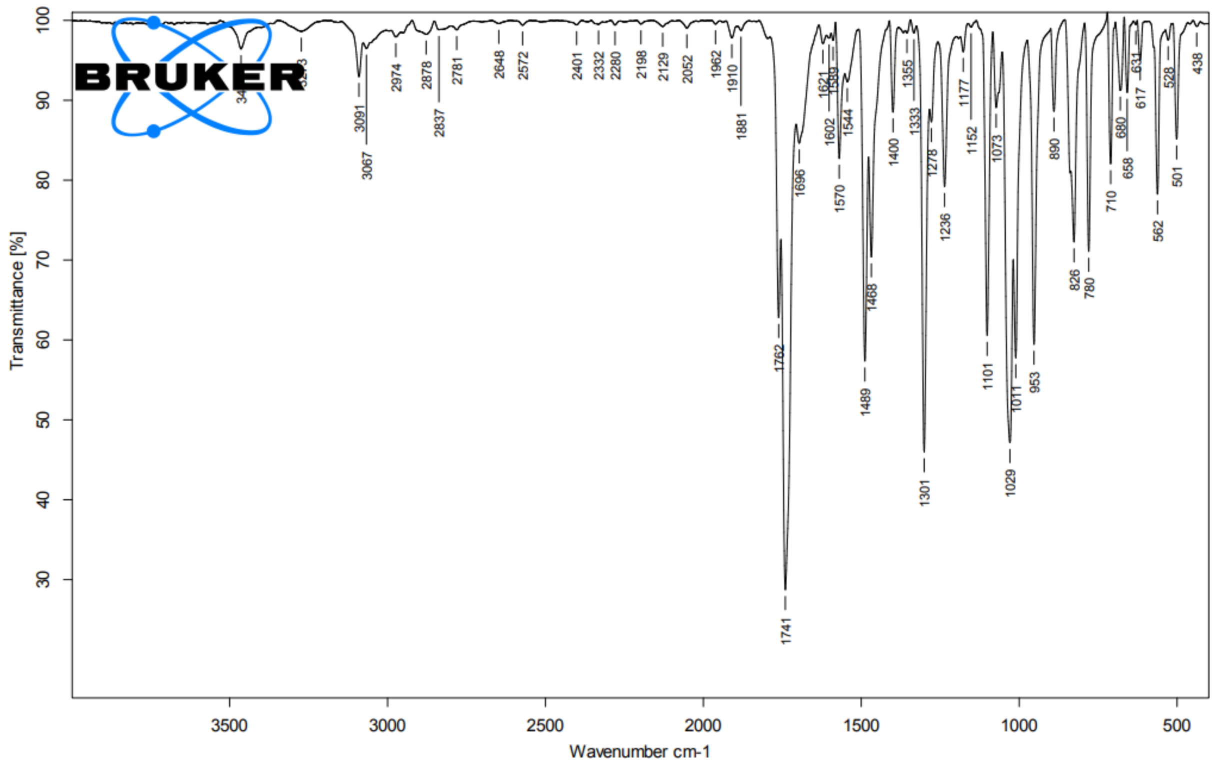

IR spectrum for compound **7c**

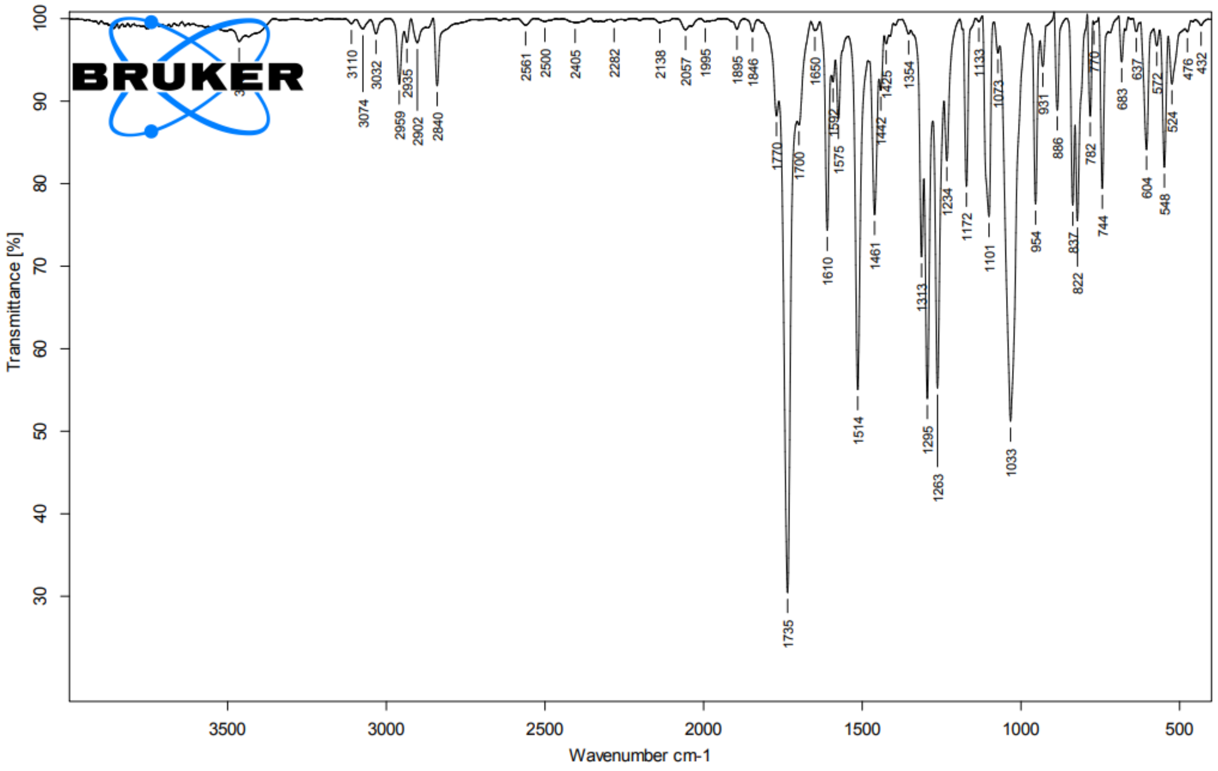

IR spectrum for compound **7d**

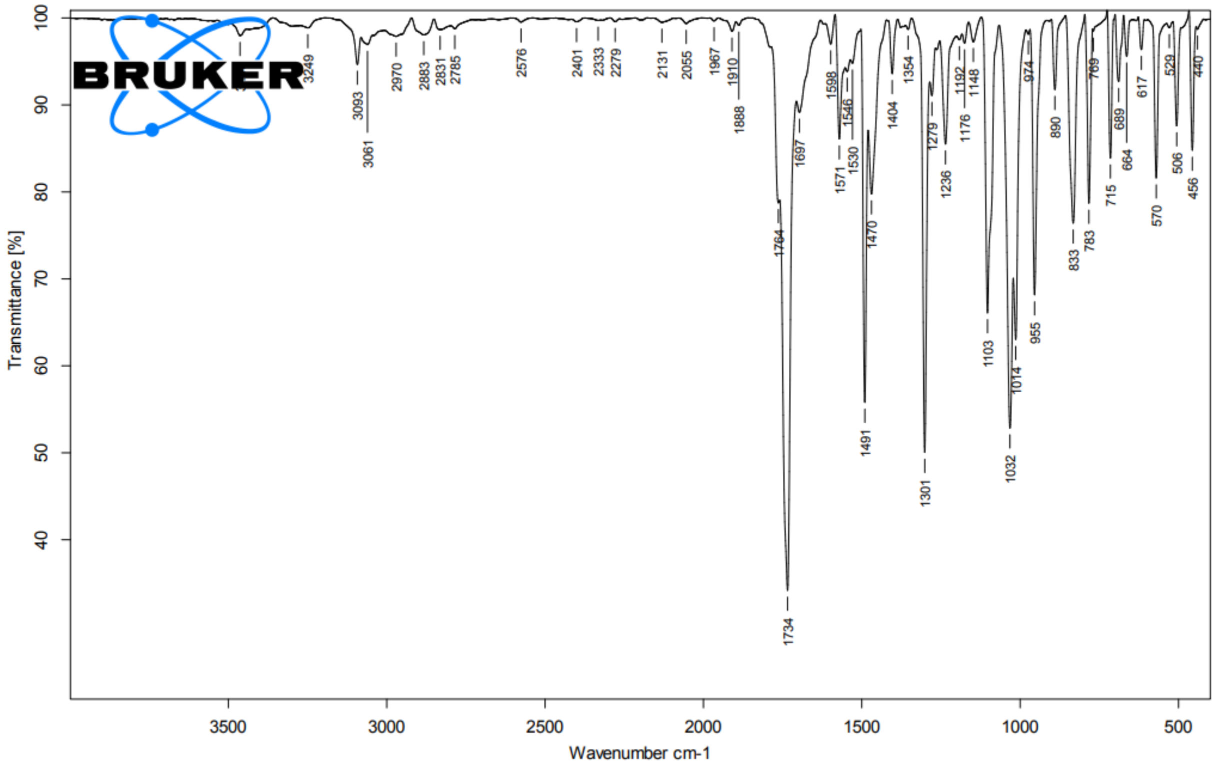

IR spectrum for compound **7e**

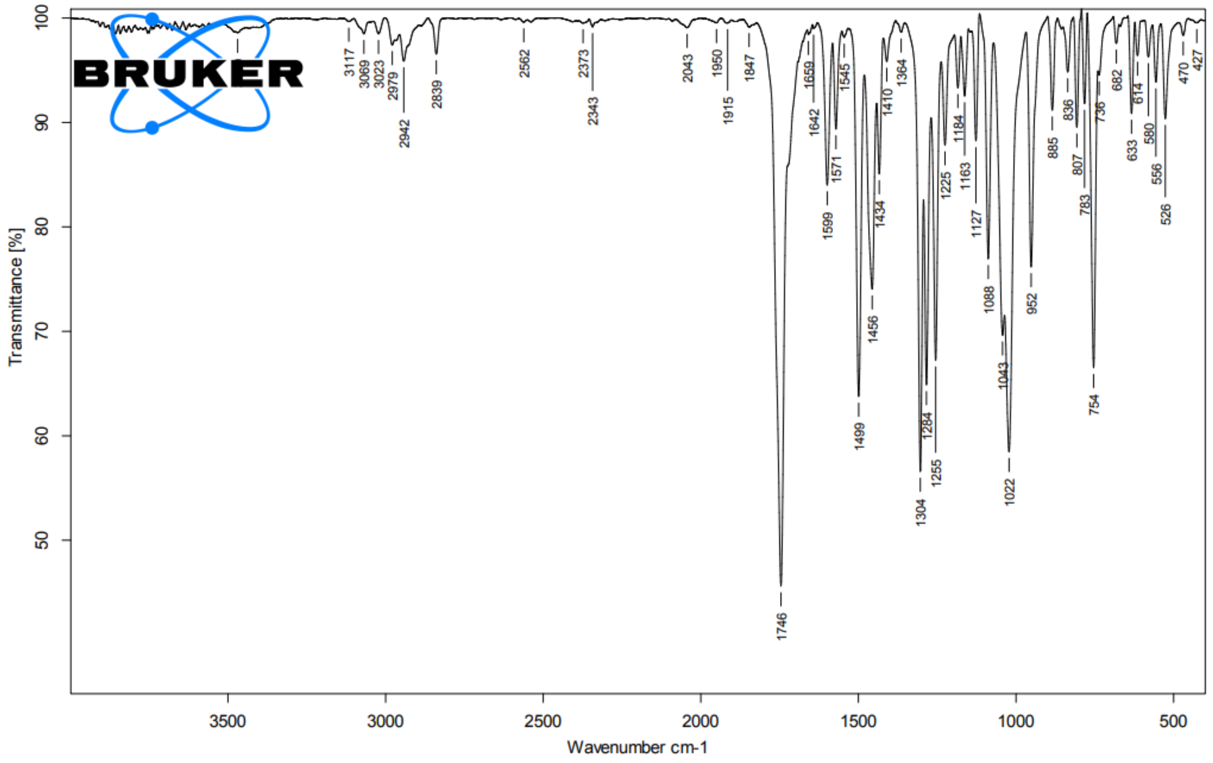

IR spectrum for compound **7f**

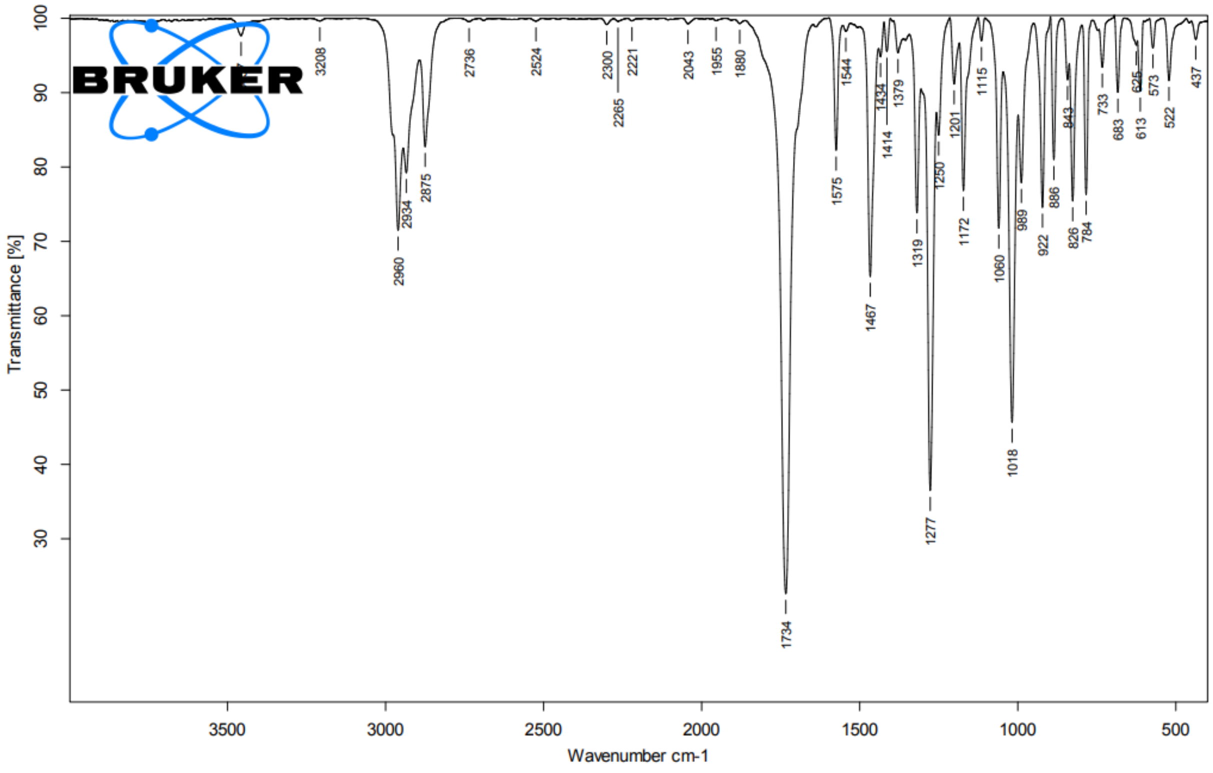

IR spectrum for compound **7g**

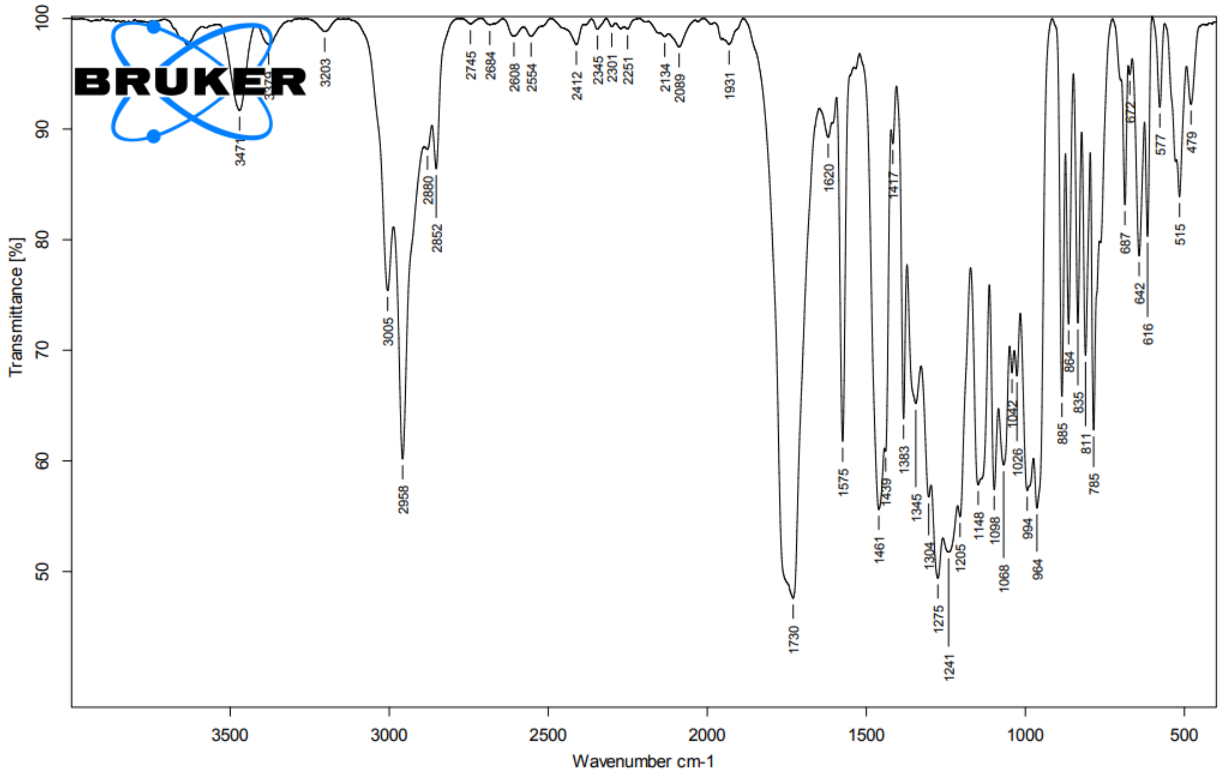

IR spectrum for compound **7h**

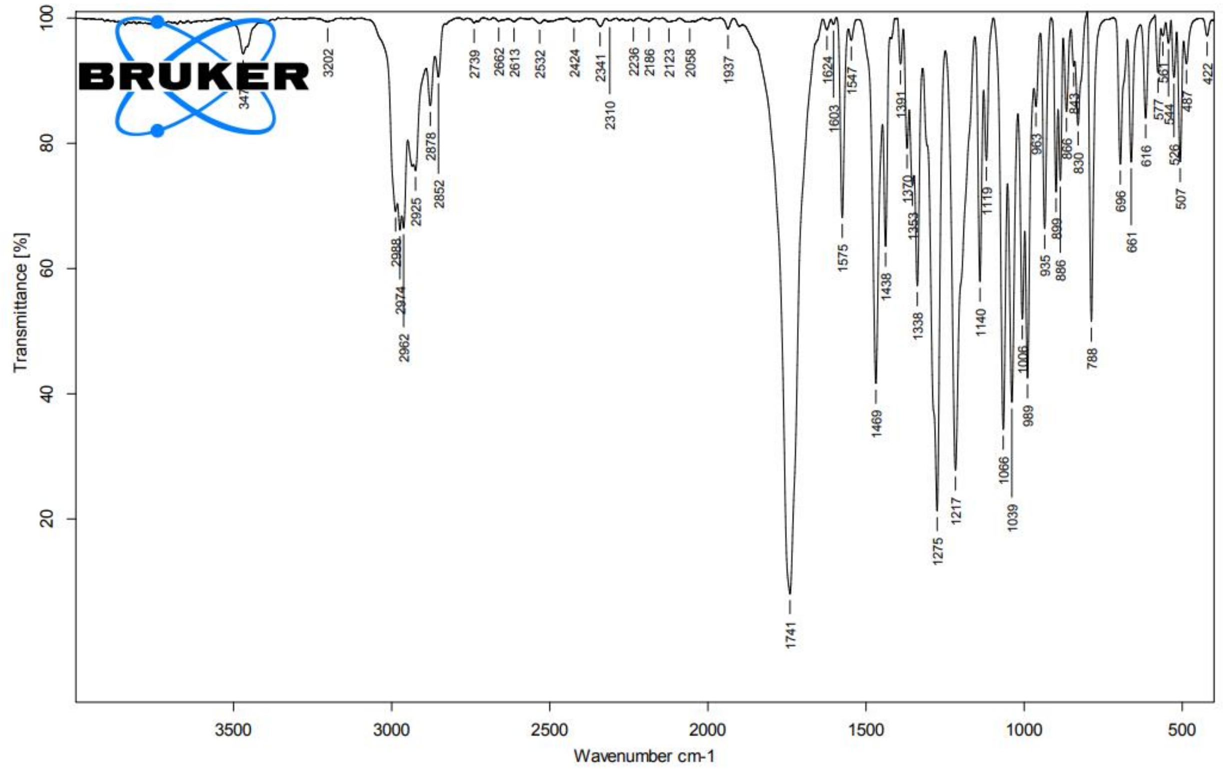

Supplement: File 1 — Experimental procedures, characterization data of all products, copies of 1H, 13C NMR, 15N spectra of new compounds, DSC curves,X-ray crystallographic data and copies of IR spectra. [file Beilstein_J_Org_Chem-20-2342-s001.pdf]
